# Maternal Smoking and the Risk of Cancer in Early Life – A Meta-Analysis

**DOI:** 10.1371/journal.pone.0165040

**Published:** 2016-11-08

**Authors:** Isabell Katharina Rumrich, Matti Viluksela, Kirsi Vähäkangas, Mika Gissler, Heljä-Marja Surcel, Otto Hänninen

**Affiliations:** 1 Department of Environmental and Biological Sciences, University of Eastern Finland, Kuopio, Finland; 2 Department of Health Protection, National Institute of Health and Welfare, Kuopio, Finland; 3 School of Pharmacy/Toxicology, Faculty of Health Sciences, University of Eastern Finland, Kuopio Finland; 4 Department of Information Services, National Institute for Health and Welfare, Helsinki, Finland and Department of Neurobiology, Care Sciences and Society, Division of Family Medicine, Karolinska Institute, Stockholm, Sweden; 5 Department of Health Protection, National Institute of Health and Welfare, Oulu, Finland; National Health Research Institutes, TAIWAN

## Abstract

**Background:**

In spite of the well-known harmful effects on the fetus, many women continue smoking during pregnancy. Smoking as an important source of toxic chemicals may contribute to the developmental origin of diseases.

**Objectives:**

The aim of this work was to pursue the possible association between maternal smoking and cancer in early life. Specifically, we wanted to identify the associated early life cancer types, and to quantify the associations.

**Methods:**

In a systematic literature search 825 articles were identified in PubMed and Web of Science, and 55 more through the reference lists. Of these 62 fulfilled the criteria for inclusion in meta-analyses. Using Mantel-Haenszel or DerSimonian and Laird method, depending on heterogeneity of the studies, pooled estimates and 95% confidence intervals for eight cancer types were calculated.

**Results:**

Smoking during pregnancy was associated with an increased risk for for brain and central nervous system tumors (OR = 1.09; 95% CI = 1.02–1.17). Although the risk for lymphoma was also associated (OR = 1.21; 95% CI = 1.05–1.34), it did not hold up in subgroup analyses. Leukemia was not found to be associated with maternal smoking. Five other cancer types (bone, soft tissue, renal, hepatic, and germ cell cancer) were also examined, but the number of studies was too limited to exclude the possibility of maternal smoking as a risk factor for cancer in offspring.

**Conclusions:**

According to our meta-analyses, maternal smoking is associated with nervous system cancers, but not with leukemia in early life. Confirming or rejecting associations of maternal smoking with lymphoma and the five other cancer types requires further studies.

## Introduction

The most common cancer types in childhood are leukemia and brain tumors which constitute over half of the malignant tumors in children [1]. The etiology of childhood cancers remains largely unknown, except for some rare genetic conditions [2]. It has long been suspected that the initiation of childhood cancer must occur during prenatal development. Yet, most childhood cancers remain unexplained and only very few exposures have been robustly associated with childhood cancer or cancer in young adulthood: maternal use of diethylstilbestrol, a synthetic estrogen, and vaginal and cervical cancer in the daughter, and x-ray exposure and leukemia [3]. Numerous other factors are suspected to be associated with increased risk of childhood cancer, but epidemiological studies are inconsistent e.g. on alcohol consumption, industrial pollutants, occupational exposures, infectious agents, maternal age, and high birth weight [4]. Special focus has been paid on maternal tobacco smoking, because of the conclusive evidence for carcinogenicity of both active and passive (second hand) cigarette smoking [5].

The prevalence of smoking in women in Western Europe ranged between 5% and 30% in 2010 [6]. The highest smoking prevalence among women was in France where about a third of women reported smoking before pregnancy. About 16% of women smoke during the first trimester in Spain and Finland, and 19% in Scotland at an unspecified point during pregnancy. Many pregnant women experience difficulties in quitting smoking. *In utero* exposures have potentially devastating effects on the unborn child due to vulnerability during the fetal development leading either to direct health effects or to a higher susceptibility to develop diseases later in life through alterations in both the genome and the epigenome [7].

Carcinogenic tobacco smoke constituents and metabolites, such as polycyclic hydrocarbons, N-nitroso compounds, their precursors, and nicotine, have been shown to cross the placenta [8, 9]. Already more than a decade ago higher concentrations of DNA adducts related to cigarette smoke carcinogens have been isolated from cord blood of smokers compared to non-smokers [1]. Furthermore, evidence for a direct association between nicotine and cancer is emerging. Both genotoxic effects and the creation of a tumor-supporting microenvironment by nicotine have been observed in vitro and in animal studies [10].

Despite this biological plausibility, epidemiological studies on maternal smoking and cancer in early life are not consistent. In the last comprehensive meta-analysis focusing on the risk for all cancers in early life, a slight increase was observed. However, the review is 15 years old, and no clear association with any specific cancer type was noted [11]. More recent reviews have focused on specific cancer types, yet they also failed to identify consistent associations. A recent meta-analysis investigating the association of different maternal factors and childhood acute lymphoblastic leukemia (ALL) found a significantly increased risk with smoking at any point in life compared to never smoking, but a more detailed analysis did not suggest a dose-response relationship [12]. Another recent meta-analysis of parental smoking and the risk of childhood brain tumors did not find any significant association between maternal smoking and childhood brain tumor risk. Subgroups analysis of different brain tumor types, study regions, study design, study sizes and publication year did not reveal any associations either [13].

No comprehensive meta-analysis looking at different childhood cancers has been published recently. By applying the same subgroup analyses to all cancers, some overarching pattern might emerge. These can hint at susceptible windows of exposure or susceptible subgroups, but as well at an underlying bias or problems in the study design. The subgroup analyses can give insight into the exposure-effect timeframe and study design considerations. As appropriate, we updated previously published meta-analyses with more recently published studies. The strength of our meta-analysis is that we applied more stringent inclusion criteria than in previous meta-analyses: in addition to a priori defined inclusion criteria, we excluded those studies, which analyzed overlapping study populations to avoid double counting. Overall, this work updates and extends previous meta-analyses by providing extensive subgroup analyses for eight childhood cancer types separately.

The aim of this work was to conduct a systematic review and meta-analysis of the possible association between maternal smoking and cancer in early life before the age of 20 years. Specifically our objective was to identify which cancer types could be most robustly associated with prenatal exposure to maternal smoking.

## Material and Methods

### Search strategy

We systematically searched PubMed and Web of Science up until June 1^st^, 2015 for original studies examining the association between maternal smoking and the risk for any types of childhood cancer. The search terms were (prenatal OR maternal) AND (smoke OR smoking OR cigarette OR tobacco) AND (cancer OR tumor OR neoplasm OR malignanc* OR leukemia OR retinoblastoma) AND (case-control OR cohort OR epidemiolog*). Reference lists of identified articles, as well as those of reviews and meta-analyses, were also searched for relevant articles. The search included any language in PubMed, but search terms were defined in English.

### Study selection

**Fig 1:**
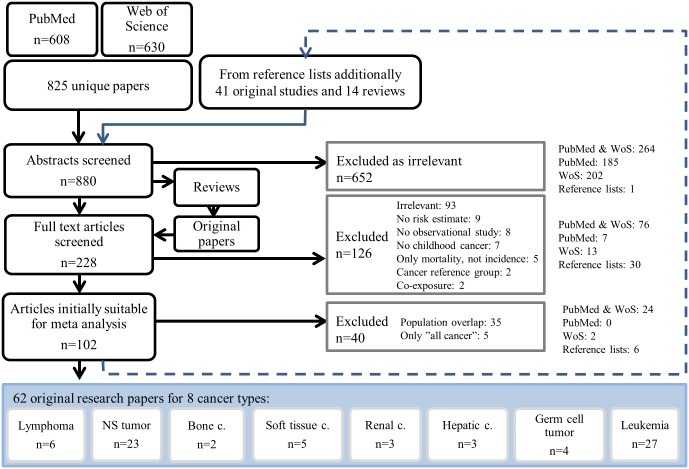
Systematic literature search. PubMed and Web of Science results and reference lists of the papers produced a total of 880 unique hits. Finally, 62 original research papers covering eight types of cancer, and 12 of their subtypes, were available for the meta-analyses (Tables A-C in S1 File). NS: nervous system; c.: cancer.

Search results were evaluated using pre-defined inclusion criteria (see below). Finally, 62 original research papers covering eight types of cancer, and 12 of their subtypes, were available for the meta-analyses (Fig 1).

Abstracts were first screened to exclude animal studies, *in vitro* studies, reviews and meta-analysis, and commentaries. The full text of the remaining citations was screened for the following inclusion criteria:

- Original epidemiological studies enrolling cancer patients younger than 20 years
- Data on maternal smoking during the index pregnancy (= pregnancy under study) were reported
- Quantitative risk estimates (odds ratio, relative risk or hazard ratio) with 95% confidence interval for each study or raw data to calculate these parameters were reported
- The reference group for calculation of risk estimates were children without cancer, whose mothers did not smoke during the index pregnancy

During the review of all relevant articles, overlaps in the study populations of the individual studies have been noted. In order to avoid double counting, the smaller studies (lower number of cases) and/or those studies with less detail, for example only qualitative smoking status assessment and no risk estimates for cigarettes smoked per day, have been excluded (Tables A and B in S1 File). The 62 original studies fulfilling the selection criteria are characterized in the Table C in S1 File.

### Data extraction

The data extracted from the articles was stored in an overview table. In case of missing information of the exposure status of cases and controls in the study, the authors were contacted to obtain these data [14, 15, 16, 17, 18, 19, 20].

### Statistical analysis

Odds ratio (OR) was used as a measure of the association between maternal smoking and the risk of childhood cancer, when case control studies (resulting in OR) and cohort studies [resulting in hazard ratio (HR) or risk ratio (RR)] were included in the same subgroup analysis. Pooled estimates and the corresponding 95% confidence intervals (CI) were calculated using either a fixed effect model or a random effect model. Some studies reported only risk estimates for categories of cigarettes smoked per day. For such studies, we calculated an estimate for the nominal smoking status (smoking vs. no smoking) by pooling all exposed cases (and controls) across the categories of cigarettes smoked per day (CPD). It was not necessary to pool unexposed cases or controls, since all studies compared the smoking categories to the same unexposed children within each study. The pooled number of exposed cases and controls was then subsequently used to calculate the OR for any smoking during pregnancy compared to no smoking during pregnancy.

The Generic Inverse Variance Method was used to assign a weight to each study included in the pooled estimate [21]. In cases where there was not enough data to construct a 2X2 table, the 95% CI was used to calculate the standard error (SE) and estimate the variance. The pooled estimates were then created using the inverse of the study variance as the weight.

The Mantel-Haenszel Method was used to calculate the pooled estimate in the fixed effect model using the above introduced weight and the logarithm of each individual risk estimate [22]. In the random effect model the DerSimonian and Laird Method was used to calculate the pooled estimate [23]. In contrast to the fixed effect model it takes into account the variance between studies.

Cochran Q statistics (p value < 0.1 as the level for significance) and Higgins et al. [24] I^2^ statistics were used to assess heterogeneity [25]. Thresholds for the interpretation of I^2^ were set according to the Cochrane Collaboration (2011a) (I^2^ < 40% no heterogeneity; I^2^ = 40–59% moderate; I^2^ = 50–79% considerable; I^2^ ≥ 80% substantial heterogeneity). Both methods produced similar results for all analyses considered here (Tables D-to K.b in S1 File). Both fixed effects and random effects model were calculated, but if there was evidence for heterogeneity, random effects model was chosen as the more reliable one.

Publication bias was assessed by funnel plots based on Egger’s regression [26].

Leave-one-out sensitivity analyses were performed, in which a pooled estimate was calculated after omission of each identified study in turn [12]. Differences between the results of sensitivity analysis were assessed with simple t-test (level of significance used was α = 0.01) (data not shown).

### Subgroup analysis

**Fig 2:**
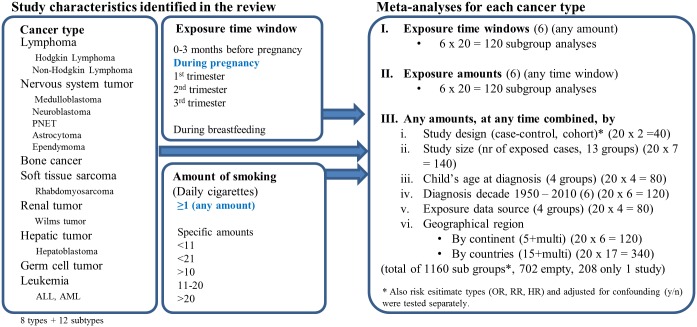
Cancer (sub)types which were analyzed according to exposure time window and exposure amount. Sensitivity analyses were conducted for nine study characteristics using studies reporting “any” smoking “during pregnancy”. Exposure amount is given in cigarettes per day (CPD). PNET = primitive neuroectodermal tumor; ALL = acute lymphoblastic leukemia; AML = acute myeloblastic leukemia.

The different cancer types were analyzed separately, but an analysis of all the cancer types grouped together was not seen to yield relevant results, due to doubts about the validity of the approach. The etiology of different cancer types differs profoundly, and it is therefore assumed that smoking has different effects on different cancer types. Overall, eight cancer types were analyzed. In addition, six of these cancer types the identified studies reported risk estimates for more specific subtypes, which were then analyzed separately in an additional subgroup analysis (Fig 2).

The level of detail of exposure information varied between the included studies. Some studies only reported “any smoking during pregnancy” with no quantification. In such a case exposure can essentially vary from occasional smoking before the knowledge of pregnancy to daily smoking throughout pregnancy.

The subgroups were pre-defined leading to many subgroups analyzing cancer subtypes, for which less than two articles were available. Mainly, the articles where grouped according to exposure time window, amount of daily smoking, study characteristics and geographical region. Each group consists of two to 17 categories (Table 1).

**Table 1:** Categories in subgroup analyses.

Subgroup	Subgroup number	Category
**Exposure time window**	1	3 months before pregnancy
	2	During pregnancy
	3	1st trimester
	4	2nd trimester
	5	3rd trimester
	6	During breastfeeding
**Amount of smoking (CPD)**	1	1 or more (“any” smoking)
	2	1–10
	3	1–20
	4	11 or more
	5	10–19
	6	21 or more
**Study characteristics**		
Study design	1	Case-control
	2	Cohort
Study size (No. exposed cases)	1	<50
	2	50–99
	3	100–149
	4	150–199
	5	200–249
	6	500–549
	7	600–469
Age at diagnosis (years)	1	0–5
	2	0–10
	3	0–14
	4	15–19
Decade of cancer diagnosis	1	1950–59
	2	1960–69
	3	1970–79
	4	1980–89
	5	1990–99
	6	2000–09
Exposure source	1	Register
	2	Interview
	3	Questionnaire
	4	Serum sample
**Geographical region**		
Continent	1	Asia
	2	Australia
	3	Europe
	4	North America
	5	South America
	6	Multiple
Country	1	Australia
	2	Brazil
	3	Canada
	4	China
	5	Colombia
	6	France
	7	Germany
	8	Greece
	9	Israel
	10	Italy
	11	Spain
	12	Sweden
	13	the Netherlands
	14	United Kingdom
	15	USA
	16	USA and Canada
	17	Multiple

For each of the eight cancer types the identified original publications were further grouped based on exposure characteristics. The criteria for the grouping included the time of exposure, such as during any point in pregnancy (reported in the original studies as “during pregnancy”), a specific trimester (first; second or third), before or after pregnancy, as well as five categories for the number of cigarettes per day. Smoking amount has been categorized into “any”, if no specific number of cigarettes per day (CPD) was reported, or into intervals (1–10 CPD; 11 and more; 10–19; 1–20; 21 and more). It was common for most articles to simply report “any” smoking “during pregnancy”, and this appeared to be the convention when discussing most cancer types. Analyses were done for each cancer type and “any smoking during pregnancy” in combination with other study characteristics. Analyses were conducted for each type of data as to smoking source (serum sample; birth register; interview or questionnaire). Aspects of study design were taken into account by categorizing data according to the type of risk estimate (OR; RR; HR) and study design (case-control; cohort). The study size was categorized based on the number of exposed cases with cut-offs in 50 intervals (<50; <100; <150; <200; and so on). The decade of cancer diagnosis was assigned based on the year of mid-point in the period in which the cancer diagnosis was given. The child age at cancer diagnosis was classified into four categories based on the upper age limit for inclusion in the original study: the category of <11 years includes all studies, which included cases diagnosed before the 11^th^ birthday, and in the age category of <15 years the diagnosis was made before the 15^th^ birthday. The category with the youngest cases had an upper limit diagnosis before the 5^th^ birthday, and the oldest age category included studies with an upper age limit for inclusion of diagnosis made between the 15^th^ and 19^th^ birthday.

**Fig 3:**
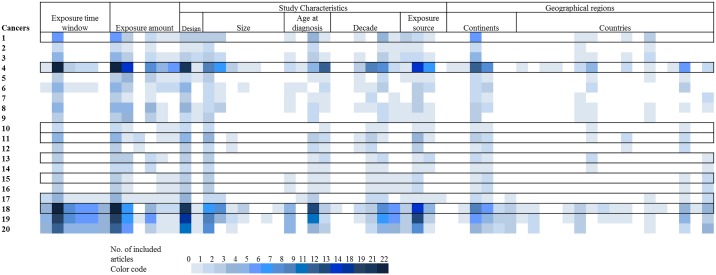
Planned subgroup analyses and number of identified articles for each analysis. Each row corresponds to one cancer (sub)type as listed in Fig 2. For column definition see Table 1.

Analyses were conducted if at least two articles were available for inclusion. Out of the total 1160 possible groups 702 were empty, and 208 contained only a single study leaving 250 groups for which the meta-analysis could be completed (Fig 3).

Overall, the studies were analyzed based on the most adjusted risk estimate, but for some studies only unadjusted risk estimates were available. These studies were categorized as adjustment of risk estimate “yes” and “no” respectively.

## Results

**Fig 4:**
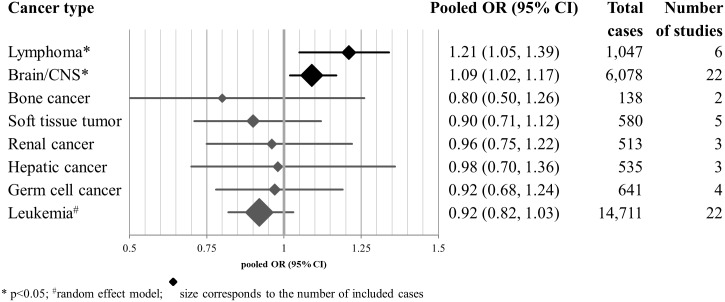
Pooled estimates of meta-analyses for eight studied cancer types. Lymphoma and brain cancers were associated with smoking during pregnancy at 95% confidence level. Random effects model was applied for leukemia only (see Tables A-K in S1 File for additional information by cancer type).

The systematic literature search identified 62 original studies covering 24 243 cancer cases published between 1982 and 2015 [27–88]. Most studies were of case-control design with only three cohort studies. More than one cancer type was analyzed in five studies, out of which three focused on lymphoma and leukemia cases, and two studies on a wider variety of cancers (Table A in S1 File). Thirty one studies reported the risk estimates based on two or more categories differentiating the amount of smoking during the pregnancy, but the other 31 studies reported risk estimates for any smoking during pregnancy. Thirteen studies reported risk estimates for smoking during specific trimester(s). The study sizes ranged from 33 to 5788 cancer cases (for total number of cases included in the meta-analysis for each cancer type, see Fig 4; for the tabulated details of all studies, see Table C in S1 File). Original studies reported cancer cases diagnosed in children younger than 15 or 19 years of age; age groups were analyzed separately as subgroups, but most analyses included all relevant studies independent from the age of diagnosis as long as it was before the age of 20 years. Information on smoking habits of the mother were mostly collected via interview or questionnaire, yet in a single study a maternal serum sample was analyzed for cotinine. Only 6 studies did not include cancer cases from a European or North American country, whereas the other 46 did.

Overall, out of the theoretically possible 1160 subgroup analyses, more than two original studies were available for 250 subgroup analyses (Fig 3). The number of articles included in each conducted subgroup analyses ranged from two to 22.

**Fig 5:**
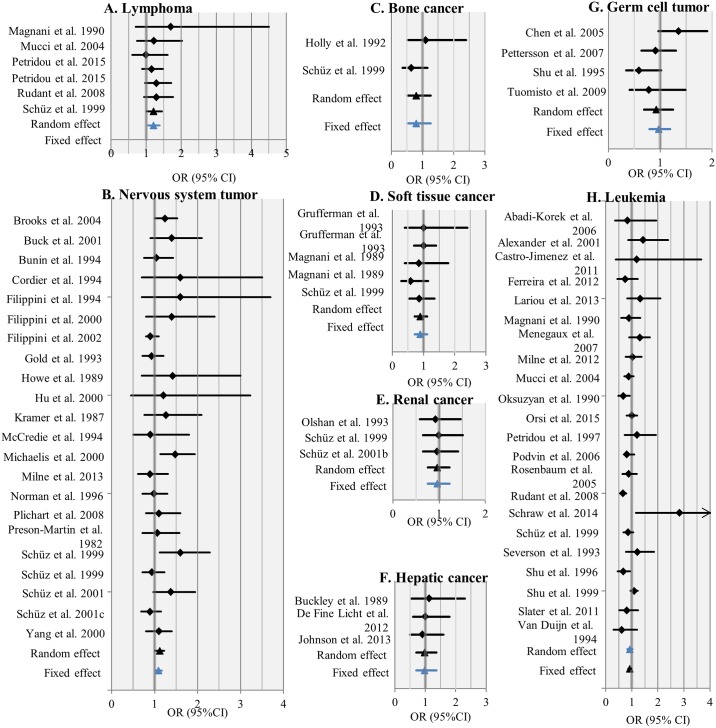
Meta-analyses based on 62 original research articles for eight cancer types and any smoking during pregnancy (yes vs no). The final model selected according to heterogeneity analyses is indicated in blue. CI = Confidence Interval.

We examined the findings on eight cancer types reported in the original studies, and we chose between fixed or random effects models based on the Cochran Q and Higgin’s I^2^ statistics. Maternal smoking during pregnancy was statistically significantly associated with early life lymphoma [OR: 1.21 (95% CI: 1.05, 1.39); 6 studies covering 269 cancer cases] and nervous system tumors [OR: 1.09 (95% CI: 1.02, 1.17); 22 studies covering 1541 cancers] (Fig 4). For the five other cancer types the associations did not reach statistical significance at the 95% confidence interval. The results for each cancer type are presented in more detail in below (Fig 5).

Sensitivity analyses using “leave one study out” method supported the robustness of the pooled estimates for both, the cancer type with statistically significant association, as well as for the seven other types with no significant association. Egger’s plot analyses indicated no significant publication bias for either the positive (lymphoma and brain cancers) nor the negative findings (Figs A-L in S1 File).

### Lymphoma

Smoking during pregnancy was associated with childhood lymphoma, including Hodgkin lymphoma and non-Hodgkin lymphoma (Table D in S1 File). Any smoking during pregnancy resulted in 21% increase in the risk [OR: 1.21 (95% CI: 1.05, 1.39)], based on the analysis of six studies (Fig 5). Case-control studies (3 studies) reported higher risk estimates reaching statistical significance in contrast to cohort studies (3 studies) with pooled ORs of 1.31 (95% CI: 1.06, 1.60) and 1.13 (95% CI: 0.92, 1.38) respectively. Due to the low number of identified studies no quantitative dose-response relationship could be assessed.

Smoking during pregnancy was associated with a 27% increase in the risk for non-Hodgkin lymphoma during childhood [OR: 1.27 (95% CI: 1.07, 1.48); 5 studies]. In general, smoking 1–10 CPD was associated with an increased risk, whereas smoking a higher number of cigarettes was not. The association was only significant in smaller studies (less than 50 exposed cases), yet not in bigger studies (50–99 exposed cases). Choice of source for smoking status of the mother seems to alter the risk estimate: pooled OR of studies using interview data was 1.53 (95% CI: 1.08, 2.14, 2 studies), whereas the pooled OR of studies using birth register data was 1.16 (95% CI: 0.93, 1.44), 2 studies) (Table D.a in S1 File).

No association between maternal smoking and risk of Hodgkin lymphoma was identified (data not shown).

### Nervous system tumors

This meta-analysis included the findings of 22 earlier studies to investigate the association between nervous system tumors and maternal smoking. In general, smoking during pregnancy was positively associated with childhood nervous system tumors [OR: 1.09 (95% CI: 1.02, 1.17); 22 studies] (Fig 5) (Table E in S1 File). The association did not follow a clear dose-response. The pooled OR for 11–20 CPD was the only exposure category to reach statistical significance. Additionally, only the smallest studies with less than 50 exposed cases showed clear association, whereas the larger studies with 50–99 or 100–149 exposed cases did not. Subgroup analyses revealed that only studies conducted in Europe found an increased risk of nervous system tumors, whereas those conducted in North America did not. Furthermore, subgroup analysis of the smoking data suggests differences based on if the pooled risk estimate of the studies was obtained via questionnaires or via interviews: in the former the pooled risk estimate was statistically significant, yet in the latter it was not. When we excluded studies not reporting adjusted risk estimates, the difference did not reach significance, whereas the pooled OR for only those studies in which the risk estimate was unadjusted did [OR: 1.15 (95% CI: 1.02, 1.29); 8 studies].

Any smoking during pregnancy was associated with a 30% increase in the risk for Neuroblastoma during early life [OR: 1.30 (95% CI: 1.10, 1.53); 5 studies]. The subgroup analyses were conducted with only two to three studies per analysis. Nevertheless, the results suggest associations with 1–10 CPD as well as 20 or more CPD, in European study populations, in studies using questionnaire data and in studies without adjustment of the original risk estimate (Table E.a in S1 File).

No associations were found with medulloblastoma, primitive neuroectodermal tumors (PNET), astrocytoma and ependymoma (data not shown).

### Leukemia

The meta-analysis of studies reporting risk estimates for smoking during pregnancy and childhood leukemia included 21 studies. The analysis suggests no association between maternal smoking during pregnancy and leukemia in early life (Fig 5) (Table K in S1 File). Maternal smoking was inversely associated with a leukemia diagnosis before the 5^th^ birthday and between the 15^th^ and 19^th^ birthday. Additionally, subgroup analysis of studies using register data for smoking information suggests a negative relationship.

The Meta-analysis of studies investigating the possible association between maternal smoking and acute lymphoblastic leukemia (ALL) were based on 19 studies (Table K.a in S1 File). Neither smoking during pregnancy nor during a specific trimester was significantly associated with ALL. Subgroup analyses of only the bigger studies (100–149 exposed cases), the age at diagnosis (0–5 years and 14–19 years), and smoking data source (birth register data) suggests an inverse relationship between maternal smoking and ALL.

Analyses of any smoking during pregnancy, as well as subgroup analyses for acute myeloblastic leukemia (AML) did not suggest an association between maternal smoking and AML. Only subgroup analysis including two studies conducted in South America yielded a significant inverse association (Table K.b in S1 File).

### Cancer types with less than 1,000 cases included in the analyses

For the remaining five cancer types the total number of cases included in the studies ranged from 138 (bone cancer) to 641 (germ cells) (Fig 4).

No clear association between maternal smoking and cancer in early life was found for the following cancer types: germ cell tumors, hepatic tumors, renal tumors, soft tissue sarcoma, and bone cancer (Tables F-J in S1 File) (Fig 5). Additionally, no clear association with maternal smoking was observed for the cancer subtypes testicular tumors, hepatoblastoma, Wilms’ tumor or rhabdomyosarcoma (data not shown).

## Discussion

In this meta-analysis, which was based on 62 studies and 24 243 cases of eight different types of cancer, statistically significant associations were identified for maternal smoking with childhood nervous system tumors and lymphoma, but not for the other cancer types included. However, the positive association for lymphoma did not hold up in the subgroup analyses including only cohort studies.

The number of lymphoma cases was 1047 and the number of nervous system tumors 6078. The number of leukemia cases was the largest (14,711), whereas cases of the five other cancer types were less frequent. The amount of cases in these cancer types ranged from 138 to 641. Thus, the positive association for nervous system tumors is supported with a larger dataset, similarly to the negative findings for leukemia.

Meta-analysis is a statistical method to pool results of different observational studies, and is therefore sensitive to uncertainties in the analysis itself, and to consequent uncertainties in the results which are used as input of the analysis. Possible sources of uncertainties are different study designs (difference in follow-up periods, exposure assessment, adjustment of risk estimate, confounding factors) of the included studies, as well as the publication bias. It has to be noted, that the included studies used various degrees of adjustment of the risk estimates. Additionally, the main focus of some studies was not maternal smoking, but it instead was only included as a confounding factor in another analysis.

Our results suggest that maternal smoking may have a protective effect especially on childhood leukemia. Although there is no clear explanation for these results, it has been hypothesized that benzo[a]pyrene, a constituent of tobacco smoke, causes generalized immunosuppression after birth. This immunosuppression caused by suppressed B-cell lymphopoiesis and induced pre-B-cell apoptosis in bone marrow cultures can lead to a lowered response to the impact of maternal smoking and with that lower risk estimates [29]. Other research suggest that the protective effect is indirect: prenatal exposure to tobacco smoke is associated with being born small-for-gestational-age. Being born small-for-gestational-age has a protective effect for childhood leukemia [78]. There is no explanation for the lack of risk (pooled OR ~ 1) for renal tumors, hepatic tumors, germ cell tumors or the statistically non-significant protective effect (pooled OR < 1) for bone cancer and soft tissue tumors. The potentially protective effect of maternal smoking on these childhood cancer types may give insight into the underlying mechanisms about the development of these cancers. These findings should not be interpreted to suggest that smoking during pregnancy would be beneficial for the unborn child. Maternal smoking is linked with several other potential health hazards/risks, such as preterm birth, fetal growth restrictions, and other birth anomalies. Also, the effects of maternal smoking on late-onset cancers in the children have only been studied in few publications, yet.

The findings in the subgroup analyses of the risk for lymphomas gives some insight into the problem of epidemiological studies of childhood cancer. Although the overall pooled risk estimate for all identified lymphoma studies was positive [OR: 1.21 (95% CI: 1.05, 1.39); 6 studies], the subgroup analyses show different results based on the study design. In the subgroup analyses of different study designs, only the subgroup with case-control design shows the same clear picture [OR: 1.30 (95% CI: 1.06, 1.60); 3 studies, 539 cases], whereas the subgroup with cohort design does not indicate a clear association between maternal smoking and childhood lymphoma [OR: 1.13 (95% CI: 0.92, 1. 38); 3 studies, 508 cases]. Both subgroups were of similar size, minimizing the chance that the differences in the pooled risk estimates are due to major differences in the statistical power. It seems likely that the differences are due to inherent differences in the study design, e.g. the risk for recall bias, identification of cases, and adjustment for confounder. This should be kept in mind when interpreting our results, or the results of any other meta-analysis. These differences and limitations are discussed below.

### Exposure misclassification

All studies included in this analysis were designed to study the association between exposure before birth and cancer in early life, before the age of twenty. Although adult cancers often have a latency time in the order of decades, the latency time for cancer in children is substantially lower with some cancers being observed already as early as at birth or at an age of less than one year. A long latency time and the use of case-control design, which was the most common design in the identified literature, can lead to exposure misclassification due to recall bias in observational studies [89]. Not only can recall bias decrease the reliability of smoking information in observational studies, but reporting bias is also an issue. Smoking during pregnancy is more and more socially unacceptable, and women tend to under-report their smoking. On the other hand, mothers of sick children tend to blame themselves for the sickness, or seek an explanation why their child suffers from cancer. Hence, they are often likely to remember and report exposures more frequently compared to e.g. control mothers. Furthermore, a change in the attitudes towards smoking, and the increasing awareness of harmful effects of smoking during pregnancy, might influence the honesty of the mother when asked about smoking during pregnancy, especially during interviews in which they have to recall their smoking habits. Prospectively collected information, as done in many cohort studies and register-based studies, reduces the reporting bias to some extent [4]. It is uncertain how much recall bias and reporting bias result in overall exposure misclassification. Nondisclosure rates to study interviewers varied between <10 and 73% across different studies. The honesty of (expecting) mothers about their smoking habits is a complicated issue, which seems to be influenced not only by the time of collecting the information, but also by the way in which the information is collected. This includes e.g. the specific phrasing of the questions in interviews and questionnaires. Overall, smoking during pregnancy is likely to continue to be generally underreported [90, 91, 92, 93]. Smoking status recorded in birth registers may be of different quality due to the recoding practices, such as level of detail or recorded only in some cases and not routinely.

### Study design

The conducted subgroup analyses hint on bias due to study design in the results. Case-control design was associated with higher risk estimates compared to a cohort design. Additionally, birth register as a source of information about maternal smoking was consistently associated with lower risk estimates compared to retrospectively collected information via interviews or questionnaires. As discussed earlier in this paper, prospectively collected information on the smoking habits of the mother are considered less biased than information collected retrospectively. It is easier to reach a sufficient number of cancer cases for statistical power in case-control setting, since the cases can be identified directly. In addition, cases and controls can be contacted directly to collect data on smoking and confounders. Cohort studies require large study populations due to the very low incidence rate of childhood cancer and a long follow-up period. It is practically impossible to collect additional data from all study participants, if the study population is too large. Hence, both study designs have their advantages and disadvantages, but it is well recognized that prospective cohort studies are more reliable than retrospective case-control designs [4]. Thus, the results of our meta-analysis may overestimate the identified risk in the light that most included studies were of case control design.

### Dose response assessment

The subgroup analyses did not suggest any clear dose-response relationship for any cancer type. Smoking 10 or less CPD was associated with a significantly increased risk of lymphoma, whereas the pooled estimate for 11 or more CPD did not reach significance. No dose response pattern emerged for nervous system tumors, either. Smoking 11–20 CPD was significantly associated with the highest risk, whereas smoking more than 20 CPD was associated with the lowest increase in risk, and did not reach statistical significance. As for leukemia, a “U” shaped dose response curve was implicated. Smoking 20 or more CPD was associated with the highest risk, which still remained below 1. The second highest risk was observed for smoking 10 or less CPD. The absence of a dose-response relationship does not support causality. It also might hint, however, at the complex molecular reactions between exposure to tobacco smoke *in utero*, changes in the DNA over years and the development of cancer. A reason for the lack of dose response might be that the exposure assessment of maternal smoking is flawed. As stated earlier, pregnant women frequently underreport the number of cigarettes per day. The resulting exposure misclassification may cover any existing dose response relationship [90, 94, 95, 96].

### Other limitations

There was no clear effect by study size on the risk estimates pattern. For lymphoma, non-Hodgkin’s lymphoma and germ cell tumors subgroup analyses for two study sizes (<50 and 50–99 participants) was possible, while for nervous system tumors, leukemia and ALL analysis of all three study size subgroups were possible (<50; 50–99; 100–149 participants). There was no consistency whether the pooled OR for the bigger studies was higher than for the smaller studies. Smaller studies usually have lower statistical power than bigger studies, which leads to higher variance in their risk estimates. However, this does not seem to heavily affect the general range of the risk estimates in our study.

As was expected, non-adjusted risk estimates were consistently higher compared to adjusted risk estimates. This pattern shows that certain choices in our study design are associated with higher risk estimates. This would in fact argue against the causal nature of the association, and instead in favor of a predominant role of bias. To minimize the effect of possible confounding in the original studies, the available most adjusted risk estimate was included in the meta-analyses. Only when no adjusted risk estimates have been reported in the original study, a non-adjusted risk estimate was used.

It has been hypothesized, that the observed increase in cancer cases in the past is partly attributed to improved diagnostics. In spite of that, sensitivity analyses of the time of the diagnosis or the publication year did not show any correlation with reported risk estimates for any cancer type. Additionally, co-exposures during pregnancy as well as exposures between birth and the development of cancer can confound the relationship. A special issue in our analysis is caused by the exposure time we focused on: we aimed at assessing the risk of maternal smoking during pregnancy and not before or after pregnancy. However, it is unlikely that a woman smokes during pregnancy and stops at birth. Hence, the observed risk is most likely confounded by exposure of the child to second hand smoke from the smoking mother, or from other household members. Most studies did not ascertain the smoking status of the mother or other household members after birth. Also, exposure of the pregnant woman to passive smoking due to other household members was not taken into account in the reviewed studies. Exposure to environmental tobacco smoke or via breastfeeding would potentially contribute to the development of cancer.

Leave-one-out sensitivity analysis was conducted omitting one study at a time, and this suggested that no specific study influenced the pooled estimate. The pooled estimates did not change significantly, regardless of which study was omitted. This supports the robustness of the pooled risk estimates. Publication bias as evaluated by Egger’s plot did not indicate significant publication bias for neither the positive nor the negative findings.

### Results in context of previous meta-analyses

This meta-analysis is an update of previously conducted meta-analyses. By carefully considering all original studies used in previous meta-analyses, we ensure that previous work is included in our analysis. The risk estimates of our analysis are directly comparable between different cancer types, since the same inclusion criteria and methods have been applied. In a first comprehensive meta-analysis Boffetta and colleagues [11] analysed all childhood cancer cases grouped no matter which cancer subtype it was. They observed an increased risk for all types of childhood cancer analyzed together [RR: 1.15 (95% CI: 1.05, 1.19)]. No association with nervous system tumors or Wilms’ tumors was found, which is partly contradicting with our results. Our analysis suggests an increases risk for brain and nervous system tumors and neuroblastoma, but this was not observed in the previous work [13, 97]. This can partly be explained by the fact that we excluded several original studies included in their work, since the study populations overlapped with bigger, epidemiological studies, which were published after Boffetta’s meta-analysis was reported. A meta-analysis focusing on childhood lymphoma reported increased risk for non-Hodgkin lymphoma, but it did not suggest any association with Hodgkin lymphoma or all lymphoma types combined [98]. In our analysis, non-Hodgkin’s lymphoma and any other type of lymphoma were associated with maternal smoking. Klimentopoulou et al. [99] reported no significant association in meta-analyses with ALL or AML [OR: 1.03 (95% CI: 0.95, 1.12) and OR: 0.99 (95% CI: 0.90, 1.09), respectively], which is in agreement with our pooled risk estimates. On the other hand, Yan et al. [11] found a slight increase in the risk for ALL due to maternal smoking [OR: 1.10 (95% CI: 1.02, 1.19)]. Again, a possible explanation can be the stricter exclusion of overlapping study populations in our work compared with Yan et al. [12].

### Implications for future studies

Reaching a sufficient study size can be a challenge, since maternal smoking does not affect the majority of children in combination with a low cancer incidence in early life. Data, which are collected routinely in health registers, enable the use of high numbers of mother-child pairs. Hence, all children born in a specific country should be included in the corresponding birth register that includes information concerning the pregnancy, including the smoking status of the mother. We believe that the use of such routinely collected health data in medical registers is the most promising approach, since the smoking information is collected during the first pregnancy checkup visit (at least in the Nordic countries). Depending on the country and the year, the smoking data are either dichotomous or categorized to smoking per day. By linking the registers, for example birth register or birth certificates with cancer registers, high numbers of data can be easily collected, without the need to contact health care professionals and mothers. This decreases the time and costs of data collection, since no interviews and questionnaires are needed. Furthermore, as the study population is the population of the country, the issue of selecting a cohort or cases and controls representing the target population is omitted. Another remaining open issue is the investigation of genetical susceptibility, especially in terms of genes modifying the effects of smoking and those which predispose to malignancies, potentially modifying the effects of carcinogenic insults. Results might give insights into the mechanisms of initiation of childhood cancers due to prenatal cigarette smoke exposure.

After our article was submitted, two prospective, population-based studies have been published. Our work is in good agreement with the results of the study by Heck and colleagues (2016) [100] for leukemia, nervous system, and neuroblastoma. For Wilms’ tumor and hepatoblastoma their estimates are substantially higher, but still do not reach significance. We do not expect that the inclusion of the study would have altered any of our results from statistical significance to non-significant or vice versa. The study by Momen et al. (2016) [101] is smaller compared to the study by Heck [99]. For leukemia the results are well comparable with the results of our meta-analysis, and therefore we do not expect that inclusion would have altered our results significantly. Their study reports no statistical significance for non-Hodgkin’s lymphoma, yet their confidence interval is very wide (95% CI:0.28–4.49), and the result is based on only 4 cases. We do not expect that this would have changed the result of our meta-analysis to a statistically non-significant risk estimate. Our pooled estimate for nervous system tumors was borderline significant (95% CI: 1.02–1.17). Momen et al [101] report a risk estimate of 0.75 (95% CI: 0.47–1.20). It is only based on 22 cancer cases. Due to the small study size and high variance in the estimate, it seems likely that it would have enough weight in our analyses to decrease our pooled risk estimate into statistical non-significance.

### Biological plausibility

Although it seems biologically plausible that *in utero* exposure to tobacco smoke is associated with an increased risk for childhood cancers, epidemiological studies have so far failed to produce strong and consistent results to establish such a causal relationship. Smoking has long been established as a cause for cancers in adults, such as cancer of the lung, urinary bladder, esophagus, oral cavity, larynx, kidney, pancreas and liver. Passive smoking has been classified as a Class I carcinogen causing lung cancer in humans by the International Agency for Research on Cancer (IARC). Cigarette smoke contains more than 5300 constituents and according to IARC, for 70 of them there is sufficient evidence for carcinogenicity in laboratory animals or humans [5]. Placental barrier with transporter proteins, as well as xenobiotic metabolism of the mother, the unborn child and the placenta determine the exposure of the offspring to different chemicals [8, 102].

There are several proposed mechanisms to how cigarette smoke contributes to cancer development after prenatal exposure. The generation of reactive oxygen species (ROS) causes oxidative damage and interferes with cell signaling pathways. Animal models using Syrian hamster showed transplacental carcinogenesis caused by 4-(methylnitrosamino)-1-(3-pyridyl)-1-butanone exposure. It is one of the IARC Class I carcinogens in cigarette smoke, and it has been detected in urine of newborns of smoking mothers, indicating a direct exposure of the unborn child to the carcinogen [102]. Another constituent of cigarette smoke, nicotine, causes the addictive characteristics of cigarettes. It crosses the blood-placenta barrier and binds to nicotine acetylcholine receptors, which are widely expressed in the fetal nervous system [9]. Evidence is emerging that nicotine can damage the genome, disrupt cellular metabolic processes, amplify oncogenes, inactivate tumor suppressor genes, and in this way promote a cancer-supporting environment [10]. The carcinogenic polycyclic aromatic hydrocarbon (PAH), benzo(a)pyrene in cigarette smoke has been shown to cross human placental barrier [103]. Evidence from animal studies supports the increased cancer risk by PAHs observed in epidemiological studies [4]. Whole body exposure of pregnant strain H mice to mainstream cigarette smoke was shown to significantly increase the incidence of cancer in the offspring compared to non-exposed controls. The cancer incidence was even higher in mice exposed both prenatally and neonatally [104].

The complexity of prenatal exposures and disease onset later in life makes it difficult to study the effects of maternal smoking on the risk of cancer development in early life. The association is modified by co-exposures and polymorphisms in genes coding for enzymes responsible for activation or detoxification of carcinogens and other chemicals. If there is only a slight increase in the risk, an inadequate number of cases or exposure misclassification can already mask the true risk. Thus far, only very few studies, with a relatively small number of cases, have analyzed drug metabolism or transporter polymorphisms in order to investigate if the effect of maternal exposure differs between genetic sub-populations. For example, genetic polymorphisms in the CYPA1 gene [105] or ABC-transporters [106] may modify the effect of smoking. Furthermore, germline variants in genes, for example CEPBA, GATA2, RUNX1 and TP53, are associated with a predisposition to hematological malignancies, potentially modifying the effect of carcinogenic insults, such as those of the tobacco smoke [107].

Additionally, it has to be taken into account that embryonic development is a tightly regulated combination of differentiation, proliferation and apoptosis of cells. This process is very strictly controlled by signaling pathways and transcription factors, which can be easily disturbed. Whichever error occurs during DNA duplication, in that time period such an error will manage to be imprinted in the DNA of a high number of subsequent cells. These errors might not directly lead to cancer, but can increase the susceptibility of cells to later insults of carcinogens [102].

## Conclusions

According to our meta-analysis, maternal smoking is associated with brain or CNS cancers and potentially lymphomas, but not with leukemia in early life. For five other cancer types the number of cancer cases in the included studies was limited, and we cannot exclude the possibility of maternal smoking as a risk factor for these cancer types in offspring. Most studies included in this meta-analysis are of case-control design relying on retrospectively collected smoking information. Thus, there is a strong likelihood of recall bias in most studies, and the results have to be interpreted with caution. Dose-response analyses did not reveal a clear relationship. Due to the heterogeneity and uncertainties in broad exposure categories a detailed analysis of specific trimesters in combination with smoking amount was not possible. Further research is needed in order to identify clear sensitive windows of exposure and possible dose-response relationships. Future studies should be included in the meta-analysis to facilitate sufficient statistical power for subgroup analysis. Additionally, effect modifiers, genetic polymorphisms and co-exposures should be similarly taken into account.

## Supporting Information

S1 File These supporting information report detailed information of studies included in this meta-analysis (S1.a. Orignal articles identified for each cancer type; S1.b. Study characterization), detailed results of subgroup analyses and publication bias assessment (S1.c. Results subgroup analyses). (DOCX)

S2 File Prisma checklist for systematic reviews and meta-analyses. (DOCX)

## Abbreviations

ALL: acute lymphoblastic leukemia
AML: acute myeloblastic leukemia
BPDE: benzo[a]pyrene diol epoxide
CI: confidence interval
CNS: central nervous system
CPD: cigarettes per day
HR: Hazard ratio
IARC: International Agency for Research on Cancer
PAH: polycyclic aromatic hydrocarbon
PNET: primitive neuroectodermal tumor
OR: odds ratio
ROS: reactive oxygen species
RR: risk ratio
SE: standard error

## References

[1] Baldwin RT, Preston-Martin S. Epidemiology of brain tumors in childhood—a review. Toxicol Appl Pharmacol 2004 9 1;199(2):118–131. 10.1016/j.taap.2003.12.029 15313584

[2] Wigle DT, Arbuckle TE, Turner MC, Berube A, Yang Q, Liu S, et al Epidemiologic evidence of relationships between reproductive and child health outcomes and environmental chemical contaminants. J Toxicol Environ Health B Crit Rev 2008 5;11(5–6):373–517. 10.1080/10937400801921320 18470797

[3] IPCS (International Programme on Chemical Safety). 2006 Principles for evaluating health risks in children associated with exposure to chemicals. Environmental Health criteria 237: 115–126.

[4] Linet MS, Wacholder S, Zahm SH. Interpreting epidemiological research: Lessons from studies of childhood cancer. Pediatrics 2003 7;112(1):218–232. 12837914

[5] IARC (International Agency for Research on Cancer). 2012 Personal habits and indoor combustions. Monogr Eval of Carcinog Risks Hum 100E: 43–211. PMC4781577 23193840

[6] Euro-Peristat project with SCPE and Eurocat. 2013. European Perinatal Health Report. The health and care of pregnant women and babies in Europe in 2010. Available: http://www.europeristat.com/reports/european-perinatal-health-report-2010.html [accessed: 3 December 2013].

[7] Pirini F, Guida E, Lawson F, Mancinelli A, Guerrero-Preston R. Nuclear and mitochondrial DNA alterations in newborns with prenatal exposure to cigarette smoke. Int J Environ Res Public Health 2015 1 22;12(2):1135–1155. 10.3390/ijerph120201135 25648174 PMC4344659

[8] Vähäkangas KH, Veid J, arttunen V, Partanen H, Sieppi E, Kummu M, Myllynen P, Loikkanen J. The significance of ABC transporters in human placenta for the exposure of fetus to xenobiotics In: Gupta Ramesh C., editor. Reproductive and developmental toxicology: Elsevier Inc; 2011 p. 1051–1065.

[9] England LJ, Bunnell RE, Pechacek TF, Tong VT, McAfee TA. Nicotine and the Developing Human: A Neglected Element in the Electronic Cigarette Debate. Am J Prev Med 2015 8;49(2):286–293. 10.1016/j.amepre.2015.01.015 25794473 PMC4594223

[10] Grando SA. Connections of nicotine to cancer. Nat Rev Cancer 2014 6;14(6):419–429. 10.1038/nrc3725 24827506

[11] Boffetta P, Tredaniel J, Greco A. Risk of childhood cancer and adult lung cancer after childhood exposure to passive smoke: A meta-analysis. Environ Health Perspect 2000 1;108(1):73–82. 10620527 10.1289/ehp.0010873 PMC1637845

[12] Yan K, Xu X, Liu X, Wang X, Hua S, Wang C, et al The associations between maternal factors during pregnancy and the risk of childhood acute lymphoblastic leukemia: A meta-analysis. Pediatr Blood Cancer 2015 7;62(7):1162–1170. 10.1002/pbc.25443 25728190

[13] Huang Y, Huang J, Lan H, Zhao G, Huang C. A Meta-Analysis of Parental Smoking and the Risk of Childhood Brain Tumors. Plos One 2014 7 24 2014;9(7):e102910 10.1371/journal.pone.0102910 25058491 PMC4109951

[14] Brondum J, Shu XO, Steinbuch M, Severson RK, Potter JD, Robison LL. Parental cigarette smoking and the risk of acute leukemia in children. Cancer 1999 3 15;85(6):1380–1388. 10189146

[15] Klebanoff MA, Clemens JD, Read JS. Maternal smoking during pregnancy and childhood cancer. Am J Epidemiol 1996 12 1;144(11):1028–1033. 8942433 10.1093/oxfordjournals.aje.a008874

[16] Kramer S, Ward E, Meadows AT, Malone KE. Medical and drug risk factors associated with neuroblastoma: a case-control study. J Natl Cancer Inst 1987 5;78(5):797–804. 3471992

[17] Castro-Jimenez MA, Orozco-Vargas LC. Parental exposure to carcinogens and risk for childhood acute lymphoblastic leukemia, Colombia, 2000–2005. Prev Chronic Dis 2011 9;8(5):A106 21843409 PMC3181179

[18] Shu XO, Linet MS, Steinbuch M, Wen WQ, Buckley JD, Neglia JP, et al Breast-feeding and risk of childhood acute leukemia. J Natl Cancer Inst 1999 10 20;91(20):1765–1772. 10528028 10.1093/jnci/91.20.1765

[19] Shu XO, Nesbit ME, Buckley JD, Krailo MD, Robinson LL. An exploratory analysis of risk factors for childhood malignant germ-cell tumors: report from the Childrens Cancer Group (Canada, United States). Cancer Causes Control 1995 5;6(3):187–198. 7612798 10.1007/BF00051790

[20] Shu XO, Ross JA, Pendergrass TW, Reaman GH, Lampkin B, Robison LL. Parental alcohol consumption, cigarette smoking, and risk of infant leukemia: a Childrens Cancer Group study. J Natl Cancer Inst 1996 1 3;88(1):24–31. 8847721 10.1093/jnci/88.1.24

[21] Cochrane Collaboration. 2011a.9.4.3 A generic inverse-variance approach to meta-analysis. In: Cochrane handbook for systematic reviews of interventions. (Higgins JPT and Green S, eds). Available: http://handbook.cochrane.org/chapter_9/9_5_2_identifying_and_measuring_heterogeneity.htm [accessed 3 December 2015].

[22] Mantel N, Haenszel W. Statistical aspects of the analysis of data from retrospective studies of disease. J Natl Cancer Inst 1959 4;22(4):719–748. 13655060

[23] DerSimonian R, Laird N. Meta-analysis in clinical trials. Control Clin Trials 1986 9;7(3):177–188. 3802833 10.1016/0197-2456(86)90046-2

[24] Higgins J, Thompson S, Deeks J, Altman D, 2003 Measuring inconsistency in meta-analyses. BMJ. 2003 9 6; 327(7414): 557–560. 10.1136/bmj.327.7414.557 12958120 PMC192859

[25] Cochrane Collaboration. 2011b.9.5.2 Identifying and measuring heterogeneity. In: Cochrane handbook for systematic reviews of interventions. (Higgins JPT and Green S, eds). Available: http://handbook.cochrane.org/chapter_9/9_5_2_identifying_and_measuring_heterogeneity.htm [accessed 3 December 2015].

[26] Egger M, Davey Smith G, Schneider M, Minder C. Bias in meta-analysis detected by a simple, graphical test. BMJ 1997 9 13;315(7109):629–634. 9310563 10.1136/bmj.315.7109.629 PMC2127453

[27] John EM, Savitz DA, Sandler DP. 1991 Prenatal exposure to parents' smoking and childhood cancer. Am J Epidemiol 133(2):123–132. 1822074 10.1093/oxfordjournals.aje.a115851

[28] Magnani C, Pastore G, Luzzatto L, Terracini B. 1990 Parental occupation and other environmental factors in the etiology of leukemias and non-Hodgkin's lymphomas in childhood: A case-control study. Tumori 76(5):413–419. 2256184 10.1177/030089169007600501

[29] Mucci LA, Granath F, Cnattingius S. 2004 Maternal smoking and childhood leukemia and lymphoma risk among 1,440,542 Swedish children. Cancer Epidemiol Biomarkers Prev 13(9):1528–1533. 15342456

[30] Petridou ET, Sergentanis TN, Skalkidou A, Antonopoulos CN, Dessypris N, Svensson T et al 2015 Maternal and birth anthropometric characteristics in relation to the risk of childhood lymphomas: A Swedish nationwide cohort study. Eur J Cancer Prev; 10.1097/CEJ.0000000000000122 25569452

[31] Rudant J, Menegaux F, Leverger G, Baruchel A, Lambilliotte A, Bertrand Y et al 2008 Childhood hematopoietic malignancies and parental use of tobacco and alcohol: The ESCALE study (SFCE). Cancer Causes Control 19(10):1277–1290. 10.1007/s10552-008-9199-5 18618277

[32] Schuz J, Kaatsch P, Kaletsch U, Meinert R, Michaelis J. 1999 Association of childhood cancer with factors related to pregnancy and birth. Int J Epidemiol 28(4):631–639. 10480689 10.1093/ije/28.4.631

[33] Brooks DR, Mucci LA, Hatch EE, Cnattingius S. 2004 Maternal smoking during pregnancy and risk of brain tumors in the offspring. A prospective study of 1.4 million swedish births. Cancer Causes Control 15(10):997–1005. 15801484 10.1007/s10552-004-1123-z

[34] Buck GM, Michalek AM, Chen CJ, Nasca PC, Baptiste MS. 2001 Perinatal factors and risk of neuroblastoma. Paediatr Perinat Epidemiol 15(1):47–53. 11237115 10.1046/j.1365-3016.2001.00307.x

[35] Bunin GR, Buckley JD, Boesel CP, Rorke LB, Meadows AT. 1994 Risk factors for astrocytic glioma and primitive neuroectodermal tumor of the brain in young children: A report from the children's cancer group. Cancer Epidemiol Biomarkers Prev 3(3):197–204. 8019366

[36] Cordier S, Iglesias MJ, Le Goaster C, Guyot MM, Mandereau L, Hemon D. 1994 Incidence and risk factors for childhood brain tumors in the ile de france. Int J Cancer 59(6):776–782. 7989118 10.1002/ijc.2910590612

[37] Filippini G, Farinotti M, Ferrarini M. 2000 Active and passive smoking during pregnancy and risk of central nervous system tumours in children. Paediatr Perinat Epidemiol 14(1):78–84. 10703038 10.1046/j.1365-3016.2000.00230.x

[38] Filippini G, Farinotti M, Lovicu G, Maisonneuve P, Boyle P. 1994 Mothers' active and passive smoking during pregnancy and risk of brain tumours in children. Int J Cancer 57(6):769–774. 8206670 10.1002/ijc.2910570602

[39] Filippini G, Maisonneuve P, McCredie M, Peris-Bonet R, Modan B, Preston-Martin S et al 2002 Relation of childhood brain tumors to exposure of parents and children to tobacco smoke: The SEARCH international case-control study. surveillance of environmental aspects related to cancer in humans. Int J Cancer 100(2):206–213. 10.1002/ijc.10465 12115571

[40] Gold EB, Leviton A, Lopez R, Gilles FH, Hedley-Whyte ET, Kolonel LN et al 1993 Parental smoking and risk of childhood brain tumors. Am J Epidemiol 137(6):620–628. 8470663 10.1093/oxfordjournals.aje.a116719

[41] Howe GR, Burch JD, Chiarelli AM, Risch HA, Choi BC. 1989 An exploratory case-control study of brain tumors in children. Cancer Res 49(15):4349–4352. 2743324

[42] Hu J, Mao Y, Ugnat AM. 2000 Parental cigarette smoking, hard liquor consumption and the risk of childhood brain tumors—a case-control study in northeast china. Acta Oncol 39(8):979–984. 11207006 10.1080/02841860050215972

[43] Kramer S, Ward E, Meadows AT, Malone KE. 1987 Medical and drug risk factors associated with neuroblastoma: A case-control study. J Natl Cancer Inst 78(5):797–804. 3471992

[44] Linet MS, Gridley G, Cnattingius S, Nicholson HS, Martinsson U, Glimelius B et al 1996 Maternal and perinatal risk factors for childhood brain tumors (sweden). Cancer Causes Control 7(4):437–448. 8813432 10.1007/BF00052670

[45] McCredie M, Maisonneuve P, Boyle P. 1994 Antenatal risk factors for malignant brain tumours in new south wales children. Int J Cancer 56(1):6–10. 8262678 10.1002/ijc.2910560103

[46] Michaelis J, Kaletsch U, Kaatsch P. 2000 Epidemiology of childhood brain tumors. Zentralbl Neurochir 61(2):80–87. 10986756 10.1055/s-2000-8264

[47] Milne E, Greenop KR, Scott RJ, Ashton LJ, Cohn RJ, de Klerk NH et al 2013 Parental smoking and risk of childhood brain tumors. Int J Cancer 133(1):253–259; 10.1002/ijc.28004 23280760

[48] Norman MA, Holly EA, Ahn DK, PrestonMartin S, Mueller BA, Bracci PM. 1996 Prenatal exposure to tobacco smoke and childhood brain tumors: Results from the united states west coast childhood brain tumor study. Cancer Epidemiology Biomarkers & Prevention 5(2):127–133. 8850274

[49] Plichart M, Menegaux F, Lacour B, Hartmann O, Frappaz D, Doz F et al 2008 Parental smoking, maternal alcohol, coffee and tea consumption during pregnancy and childhood malignant central nervous system tumours: The ESCALE study (SFCE). Eur J Cancer Prev 17(4):376–383. 10.1097/CEJ.0b013e3282f75e6f 18562965 PMC2746823

[50] Preston-Martin S, Yu MC, Benton B, Henderson BE. 1982 N-nitroso compounds and childhood brain tumors: A case-control study. Cancer Res 42(12):5240–5245. 7139628

[51] Schuz J, Kaletsch U, Kaatsch P, Meinert R, Michaelis J. 2001a Risk factors for pediatric tumors of the central nervous system: Results from a German population-based case-control study. Med Pediatr Oncol 36(2):274–282. 11452935 10.1002/1096-911X(20010201)36:2<274::AID-MPO1065>3.0.CO;2-D

[52] Schuz J, Kaletsch U, Meinert R, Kaatsch P, Spix C, Michaelis J. 2001c Risk factors for neuroblastoma at different stages of disease. results from a population-based case-control study in Germany. J Clin Epidemiol 54(7):702–709. 11438411 10.1016/s0895-4356(00)00339-5

[53] Yang Q, Olshan AF, Bondy ML, Shah NR, Pollock BH, Seeger RC et al 2000 Parental smoking and alcohol consumption and risk of neuroblastoma. Cancer Epidemiol Biomarkers Prev 9(9):967–972. 11008916

[54] Holly EA, Aston DA, Ahn DK, Kristiansen JJ. 1992 Ewing's bone sarcoma, paternal occupational exposure, and other factors. Am J Epidemiol 135(2):122–129. 1311140 10.1093/oxfordjournals.aje.a116265

[55] Grufferman S, Schwartz AG, Ruymann FB, Maurer HM. 1993 Parents' use of cocaine and marijuana and increased risk of rhabdomyosarcoma in their children. Cancer Causes Control 4(3):217–224. 8318638 10.1007/BF00051316

[56] Grufferman S, Wang HH, DeLong ER, Kimm SY, Delzell ES, Falletta JM. 1982 Environmental factors in the etiology of rhabdomyosarcoma in childhood. J Natl Cancer Inst 68(1):107–113. 6948120

[57] Magnani C, Pastore G, Luzzatto L, Carli M, Lubrano P, Terracini B. 1989 Risk factors for soft tissue sarcomas in childhood: A case-control study. Tumori 75(4):396–400. 2815346 10.1177/030089168907500418

[58] Olshan AF, Breslow NE, Falletta JM, Grufferman S, Pendergrass T, Robison LL et al 1993 Risk factors for Wilms’ tumor. report from the national Wilms’ tumor study. Cancer 72(3):938–944. 8392906 10.1002/1097-0142(19930801)72:3<938::aid-cncr2820720345>3.0.co;2-c

[59] Schuz J, Kaletsch U, Meinert R, Kaatsch P, Michaelis J. 2001b High birth weight and other risk factors for Wilms’ tumour: Results of a population-based case-central study. Eur J Pediatr 160(6):333–338. 11421411 10.1007/pl00008443

[60] Buckley JD, Sather H, Ruccione K, Rogers PC, Haas JE, Henderson BE et al 1989 A case-control study of risk factors for hepatoblastoma. A report from the children’s cancer study group. Cancer 64(5):1169–1176. 2547509 10.1002/1097-0142(19890901)64:5<1169::aid-cncr2820640534>3.0.co;2-i

[61] de Fine Licht S, Schmidt LS, Rod NH, Schmiegelow K, Lahteenmaki PM, Kogner P et al 2012 Hepatoblastoma in the Nordic countries. Int J Cancer 131(4):E555–61. 10.1002/ijc.27351 22095187

[62] Johnson KJ, Williams KS, Ross JA, Krailo MD, Tomlinson GE, Malogolowkin MH et al 2013 Parental tobacco and alcohol use and risk of hepatoblastoma in offspring: A report from the children's oncology group. Cancer Epidemiol Biomarkers Prev 22(10):1837–1843. 10.1158/1055-9965.EPI-13-0432 23950215 PMC4188411

[63] Chen Z, Robison L, Giller R, Krailo M, Davis M, Gardner K et al 2005 Risk of childhood germ cell tumors in association with parental smoking and drinking. Cancer 103(5):1064–1071. 10.1002/cncr.20894 15685619

[64] Pettersson A, Akre O, Richiardi L, Ekbom A, Kaijser M. 2007 Maternal smoking and the epidemic of testicular cancer—a nested case-control study. Int J Cancer 120(9):2044–2046. 10.1002/ijc.22523 17278105

[65] Shu XO, Nesbit ME, Buckley JD, Krailo MD, Robinson LL. 1995 An exploratory analysis of risk factors for childhood malignant germ-cell tumors: Report from the children’s cancer group (Canada, United States). Cancer Causes Control 6(3):187–198. 7612798 10.1007/BF00051790

[66] Tuomisto J, Holl K, Rantakokko P, Koskela P, Hallmans G, Wadell G et al 2009 Maternal smoking during pregnancy and testicular cancer in the sons: A nested case-control study and a meta-analysis. Eur J Cancer 45(9):1640–1648;. 10.1016/j.ejca.2009.01.017 19231156

[67] Abadi-Korek I, Stark B, Zaizov R, Shaham J. Parental occupational exposure and the risk of acute lymphoblastic leukemia in offspring in israel.J Occup Environ Med.2006 2;48(2):165–74. 10.1097/01.jom.0000183343.81485.7c 16474265

[68] Alexander FE, Patheal SL, Biondi A, Brandalise S, Cabrera ME, Chan LC et al 2001 Transplacental chemical exposure and risk of infant leukemia with MLL gene fusion. Cancer Res 61(6):2542–2546. 11289128

[69] Brondum J, Shu XO, Steinbuch M, Severson RK, Potter JD, Robison LL. 1999 Parental cigarette smoking and the risk of acute leukemia in children. Cancer 85(6):1380–1388. 10189146

[70] Castro-Jimenez MA, Orozco-Vargas LC. 2011 Parental exposure to carcinogens and risk for childhood acute lymphoblastic leukemia, Colombia, 2000–2005. Prev Chronic Dis 8(5):A106 21843409 PMC3181179

[71] Farioli A, Legittimo P, Mattioli S, Miligi L, Benvenuti A, Ranucci A et al 2014 Tobacco smoke and risk of childhood acute lymphoblastic leukemia: Findings from the SETIL case-control study. Cancer Causes Control 25(6):683–692. 10.1007/s10552-014-0371-9 24699944

[72] Ferreira JD, Couto AC, Pombo-de-Oliveira MS, Koifman S, Brazilian Collaborative Study Group of Infant Acute Leukemia. 2012 Pregnancy, maternal tobacco smoking, and early age leukemia in brazil. Front Oncol 2:151 10.3389/fonc.2012.00151 23162789 PMC3494108

[73] Infante-Rivard C, Krajinovic M, Labuda D, Sinnett D. 2000 Parental smoking, CYP1A1 genetic polymorphisms and childhood leukemia (quebec, canada). Cancer Causes Control 11(6):547–553. 10880037 10.1023/a:1008976116512

[74] Lariou MS, Dikalioti SK, Dessypris N, Baka M, Polychronopoulou S, Athanasiadou-Piperopoulou F et al 2013 Allergy and risk of acute lymphoblastic leukemia among children: A nationwide case control study in greece. Cancer Epidemiol 37(2):146–151. 10.1016/j.canep.2012.10.012 23182223

[75] Mattioli S, Farioli A, Legittimo P, Miligi L, Benvenuti A, Ranucci A et al 2014 Tobacco smoke and risk of childhood acute non-lymphocytic leukemia: Findings from the SETIL study. PLoS One 9(11):e111028 10.1371/journal.pone.0111028 25401754 PMC4234298

[76] Menegaux F, Ripert M, Hemon D, Clavel J. 2007 Maternal alcohol and coffee drinking, parental smoking and childhood leukaemia: A French population-based case-control study. Paediatr Perinat Epidemiol 21(4):293–299. 10.1111/j.1365-3016.2007.00824.x 17564585

[77] Milne E, Greenop KR, Scott RJ, Bailey HD, Attia J, Dalla-Pozza L et al 2012 Parental prenatal smoking and risk of childhood acute lymphoblastic leukemia. Am J Epidemiol 175(1):43–53. 10.1093/aje/kwr275 22143821

[78] Oksuzyan S, Crespi CM, Cockburn M, Mezei G, Kheifets L. 2012 Birth weight and other perinatal characteristics and childhood leukemia in California. Cancer Epidemiol 36(6):e359–65. 10.1016/j.canep.2012.08.002 22926338 PMC4034745

[79] Orsi L, Rudant J, Ajrouche R, Leverger G, Baruchel A, Nelken B et al 2015 Parental smoking, maternal alcohol, coffee and tea consumption during pregnancy, and childhood acute leukemia: The ESTELLE study. Cancer Causes Control; 10.1007/s10552-015-0593-5 25956268

[80] Petridou E, Trichopoulos D, Kalapothaki V, Pourtsidis A, Kogevinas M, Kalmanti M et al 1997 The risk profile of childhood leukaemia in greece: A nationwide case-control study. Br J Cancer 76(9):1241–1247. 9365177 10.1038/bjc.1997.541 PMC2228112

[81] Podvin D, Kuehn CM, Mueller BA, Williams M. 2006 Maternal and birth characteristics in relation to childhood leukaemia. Paediatr Perinat Epidemiol 20(4):312–322. 10.1111/j.1365-3016.2006.00731.x 16879503

[82] Rosenbaum PF, Buck GM, Brecher ML. 2005 Allergy and infectious disease histories and the risk of childhood acute lymphoblastic leukaemia. Paediatr Perinat Epidemiol 19(2):152–164. 10.1111/j.1365-3016.2005.00634.x 15787890

[83] Schraw JM, Dong YQ, Okcu MF, Scheurer ME, Forman MR. 2014 Do longer formula feeding and later introduction of solids increase risk for pediatric acute lymphoblastic leukemia? Cancer Causes Control 25(1):73–80. 10.1007/s10552-013-0309-7 24154567

[84] Severson RK, Buckley JD, Woods WG, Benjamin D, Robison LL. 1993 Cigarette smoking and alcohol consumption by parents of children with acute myeloid leukemia: An analysis within morphological subgroups—a report from the childrens cancer group. Cancer Epidemiol Biomarkers Prev 2(5):433–439. 8220087

[85] Shu XO, Ross JA, Pendergrass TW, Reaman GH, Lampkin B, Robison LL. 1996 Parental alcohol consumption, cigarette smoking, and risk of infant leukemia: A children’s cancer group study. J Natl Cancer Inst 88(1):24–31. 8847721 10.1093/jnci/88.1.24

[86] Shu XO, Linet MS, Steinbuch M, Wen WQ, Buckley JD, Neglia JP et al 1999 Breast-feeding and risk of childhood acute leukemia. J Natl Cancer Inst 91(20):1765–1772. 10528028 10.1093/jnci/91.20.1765

[87] Slater ME, Linabery AM, Blair CK, Spector LG, Heerema NA, Robison LL et al 2011 Maternal prenatal cigarette, alcohol and illicit drug use and risk of infant leukaemia: A report from the children's oncology group. Paediatr Perinat Epidemiol 25(6):559–565. 10.1111/j.1365-3016.2011.01229.x 21980945 PMC3614405

[88] van Duijn CM, van Steensel-Moll HA, Coebergh JW, van Zanen GE. 1994 Risk factors for childhood acute non-lymphocytic leukemia: An association with maternal alcohol consumption during pregnancy? Cancer Epidemiol Biomarkers Prev 3(6):457–460. 8000294

[89] Grimes DA, Schulz KF. 2002 Bias and causal association in observational research. Lancet 359: 248–252. 10.1016/S0140-6736(02)07451-2 11812579

[90] Picket KE, Rathouz PJ, Kasza K, Wakschlag L, Wright R. 2005 Self-reported smoking cotinine levels, and patterns of smoking in pregnancy. Paediatr Perinat Epidemiol 19(5):368–376. 10.1111/j.1365-3016.2005.00660.x 16115289

[91] Walsh AR, Redman S, Adamson L. 1996 The accuracy of self-report of smoking status in pregnant women. Addict Behav 21(5):675–679. 8876767 10.1016/0306-4603(95)00097-6

[92] Britton GRA, Brinthaupt J, Stehle JM, James GD. 2004 Comparison of self-reported smoking and urinary cotinine levels in a rural pregnant population. JOGNN 33(3):306–311. 15180193 10.1177/0884217504264866

[93] Web DA, Boyd NR, Messina D, Windsor RA. 2003 The discrepancy between self-reported smoking status and urine cotinine levels among women enrolled in prenatal care at four publicly funded clinical sites. J Public Health Manag Pract 9(4):322–325. 12836515 10.1097/00124784-200307000-00011

[94] Lindqvist R, Lendhals L, Tollbom Ö, Åberg H, Håkansson A. 2002 Smoking during pregnancy: comparison of self-reports and cotinine levels in 496 women. Acta Obstet Gynecol Scand 81(3):240–244. 11966481 10.1034/j.1600-0412.2002.810309.x

[95] Swamy G, Reddick KLB, Brouwer RJN, Pollak KI Myers ER. 2011 Smoking prevalence in early pregnancy: comparison of self-report and anonymous urine cotinine testing. J Maternal Fetal Neonatal Med 24(1):86–90. 10.3109/14767051003758887 PMC3624613 20384470

[96] Ford RPK, Tappin DM, Schluter PJ, Wild CJ. 1997 Smoking during pregnancy: how reliable are maternal self reports in New Zealand? J Epidemiol Community Health 51(3):246–251. 9229052 10.1136/jech.51.3.246 PMC1060468

[97] Huncharek M, Kupelnick B, Klassen H. Maternal smoking during pregnancy and the risk of childhood brain tumors: a meta-analysis of 6566 subjects from twelve epidemiological studies. J Neurooncol 2002 3;57(1):51–57. 12125967 10.1023/a:1015734921470

[98] Antonopoulos CN, Sergentanis TN, Papadopoulou C, Andrie E, Dessypris N, Panagopoulou P, et al Maternal smoking during pregnancy and childhood lymphoma: a meta-analysis. Int J Cancer 2011 12 1;129(11):2694–2703. 10.1002/ijc.25929 21225624

[99] Klimentopoulou A, Antonopoulos CN, Papadopoulou C, Kanavidis P, Tourvas AD, Polychronopoulou S, et al Maternal smoking during pregnancy and risk for childhood leukemia: a nationwide case-control study in Greece and meta-analysis. Pediatr Blood Cancer 2012 3;58(3):344–351. 10.1002/pbc.23347 21990018

[100] Heck JE, Contreras ZA, Park AS, Davidson TB, Cockburn M, Ritz B. 2016 Smoking in pregnancy and risk of cancer among young children: A population-based stuy. Int J Cancer 139(3):613–616. 10.1002/ijc.30111 27016137 PMC4896308

[101] Momen NC, Olsen J, Gissler M, Li J. 2015 Exposure to maternal smoking during pregnancy and risk of childhood cancer: a study using Danish national registers. Cancer Causes Control 27(3):341–349. 10.1007/s10552-015-0707-0 26689564

[102] Wan J, Winn LM. In utero-initiated cancer: the role of reactive oxygen species. Birth Defects Res C Embryo Today 2006 12;78(4):326–332. 10.1002/bdrc.20080 17315246

[103] Karttunen V, Myllynen P, Prochazka G, Pelkonen O, Segerback D, Vahakangas K. Placental transfer and DNA binding of benzo(a)pyrene in human placental perfusion. Toxicol Lett 2010 8 16;197(2):75–81. 10.1016/j.toxlet.2010.04.028 20466050

[104] Balansky R, Ganchev G, Iltcheva M, Nikolov M, Steele VE, De Flora S. Differential carcinogenicity of cigarette smoke in mice exposed either transplacentally, early in life or in adulthood. Int J Cancer 2012 3 1;130(5):1001–1010. 10.1002/ijc.26103 21484788

[105] Infante-Rivard C, Krajinovic M, Labuda D, Sinnett D. Parental smoking, CYP1A1 genetic polymorphisms and childhood leukemia (Quebec, Canada). Cancer Causes Control 2000 7;11(6):547–553. 10880037 10.1023/a:1008976116512

[106] Vähäkangas K, Myllynen P. 2009 Drug transporters in the human blood-placental barrier. Br J Pharmacol 158(3):665–78. 10.1111/j.1476-5381.2009.00336.x 19788499 PMC2765588

[107] Haferlach T. 2015 Why germline variations in ALL can matter. Lancet 16:1577–1578. 10.1016/S1470-2045(15)00440-4 26522333

