# Supplementary material for: Maternal Smoking and the Risk of Cancer in Early Life – A Meta-Analysis
Source: PLoS One. 2016 Nov 8;11(11):e0165040. doi: 10.1371/journal.pone.0165040 (PMC5100920; doi:10.1371/journal.pone.0165040)
Supplement: S1 File — (DOCX) [file pone.0165040.s001.docx]

***S1 File***

*Supplemental Material*

**Maternal smoking and the risk of cancer in early life– A meta-analysis**

Isabell Rumrich*^1,2^, Matti Viluksela^1,2^, Kirsi Vähäkangas^3^, Mika Gissler^4^, Heljä-Marja Surcel^4^, Otto Hänninen^2^

* Corresponding author

^1^ Department of Environmental and Biological Sciences, University of Eastern Finland, Kuopio, Finland

^2^ Department of Health Protection, National Institute of Health and Welfare, Kuopio, Finland

^3^ School of Pharmacy, University of Eastern Finland, Kuopio Finland

^4^ Department of Health Protection, National Institute of Health and Welfare, Oulu, Finland

^5^ Department of Information Services, National Institute for Health and Welfare, Helsinki, Finland and Department of Neurobiology, Care Science and Society, Division of Family Medicine, Karolinska Institute, Stockholm Sweden

Table of Contents

[S1.a.Original articles identified for each cancer type 3](#_Toc465233740)

[Table A. Included articles in analyses of each cancer type 3](#_Toc465233741)

[Table B. Excluded studies due to overlaps in the study population with other studies. 5](#_Toc465233742)

[S1.b. Study characterization 6](#_Toc465233743)

[Table C. In the meta-analysis included original studies maternal smoking during pregnancy and childhood cancer. 7](#_Toc465233744)

[S1.c. Results subgroup analyses 25](#_Toc465233745)

[Table D. Subgroup analyses of risk for lymphoma in early life due to maternal smoking 26](#_Toc465233746)

[Table D.a. Subgroup analyses of risk for non-Hodgkin’s lymphoma in early life due to maternal smoking 27](#_Toc465233747)

[Table E. Subgroup analyses of risk for nervous system tumors in early life due to maternal smoking 28](#_Toc465233748)

[Table E.a. Subgroup analyses of risk for neuroblastoma in early life due to maternal smoking 30](#_Toc465233749)

[Table F. Subgroup analyses of risk for bone cancer in early life due to maternal smoking 31](#_Toc465233750)

[Table G. Subgroup analyses of risk for soft tissue tumors in early life due to maternal smoking 32](#_Toc465233751)

[Table H. Subgroup analyses of risk for renal tumors in early life due to maternal smoking 33](#_Toc465233752)

[Table I. Subgroup analyses of risk for hepatic tumors in early life due to maternal smoking 34](#_Toc465233753)

[Table J. Subgroup analyses of risk for germ cell tumors in early life due to maternal smoking 35](#_Toc465233754)

[Table K. Subgroup analyses of risk for leukemia in early life due to maternal smoking 36](#_Toc465233755)

[Table K.a. Subgroup analyses of risk for acute lymphoblastic leukemia in early life due to maternal smoking 38](#_Toc465233756)

[Table K.b. Subgroup analyses of risk for acute myeloblastic leukemia in early life due to maternal smoking 40](#_Toc465233757)

[Risk of Publication bias 42](#_Toc465233758)

[S1.d. References 47](#_Toc465233759)

# S1.a.Original articles identified for each cancer type

Overall 62 original research articles on maternal smoking and cancer in early life have been identified for eight cancer subtypes in a systematic literature search in PubMed and Web of Science. Out of the 62 articles only five reported risk estimates for more than one cancer type and are therefore listed multiple times in the table below. The given study ID in the table below corresponds to the study ID in the review table (Table S3).

#### Table A. Included articles in analyses of each cancer type

| **Cancer type** | **Included articles [Reference]** | **Study ID^*^** |
| --- | --- | --- |
| Lymphoma | John et al. 1991 [1]  Magnani et al. 1990 [2]  Mucci et al. 2004 [3]  Petridou et al. 2015 [4]  Rudant et al. 2008 [5]  Schüz et al. 1999 [6] | 24 ^  29 ^  37 ^†  43 †  49  51 |
| Nervous system | Brooks et al. 2004 [7]  Buck et al. 2001 [8]  Bunin et al. 1994 [9]  Cordier et al. 1994 [10]  Filippini et al. 1994 [11]  Filippini et al. 2000 [12]  Filippini et al. 2002 [13]  Gold et al. 1993 [14]  Howe et al. 1989 [15]  Hu et al. 2000 [16]  John et al. 1991 [1]  Kramer et al. 1987 [17]  Linet et al. 1996 [18]  McCredie et al. 1994 [19]  Michaelis et al. 2000 [20]  Milne et al. 2013 [21]  Norman et al. 1996 [22]  Plichart et al. 2008 [23]  Preston-Martin et al. 1982 [24]  Schüz et al. 1999 [6]  Schüz et al. 2001a [25]  Schüz et al. 2001c [26]  Yang et al. 2000 [27] | 4 †  5  7  10  14  15  16  17  21  22  24 ^  26  28  32  34  36  38  45  47  51  52  54  62 |
| Bone cancer | Holly et al. 1992 [28]  Schüz et al. 1999 [6] | 20  51 |
| Soft tissue cancer | Grufferman et al. 1982 [29]  Grufferman et al. 1993 [30]  John et al. 1991 [1]  Magnani et al. 1989 [31]  Schüz et al. 1999 [6] | 18  19  24 ^  30  51 |
| Renal cancer | Olshan et al. 1993 [32]  Schüz et al. 1999 [6]  Schüz et al. 2001b [33] | 40  51  53 |
| Hepatic cancer | Buckley et al. 1989 [34]  de Fine Licht et al. 2012 [35]  Johnson et al. 2013 [36] | 6  11  25 |
| Germ cell tumor | Chen et al. 2005 [37]  Pettersson et al. 2007 [38]  Shu et al. 1995 [39]  Tuomisto et al. 2009 [40] | 9  44  56  60 |
| Leukemia | Abadi-Korek et al. 2006 [41]  Alexander et al. 2001 [42]  Brondum et al. 1999 [43]  Castro-Jimenez & Orozco-Vargas, 2011 [44]  Farioli et al. 2014 [45]  Ferreira et al. 2012 [46]  Infante-Rivard et al. 2000 [47]  John et al. 1991 [1]  Lariou et al. 2013 [48]  Magnani et al. 1990 [2]  Mattioli et al. 2014 [49]  Menegaux et al. 2007 [50]  Milne et al. 2012 [51]  Mucci et al. 2004 [3]  Oksuzyan et al. 2012 [52]  Orsi et al. 2015 [53]  Petridou et al. 1997 [54]  Podvin et al. 2006 [55]  Rosenbaum et al. 2005 [56]  Rudant et al. 2008 [5]  Schraw et al. 2014 [57]  Schüz et al. 1999 [6]  Severson et al. 1993 [58]  Shu et al. 1996 [59]  Shu et al. 1999 [60]  Slater et al. 2011 [61]  van Dujin et al. 1994 [62] | 1  2  3  8  12  13  23  24  27  29  31  33  35  37 ^†  39  41  42  46  48  49  50  51  55  57  58  59  61 |

* corresponds to Study ID in the review table (Table S3); ^ Study reports risk estimates for more than one cancer type; † cohort study

Many studies have been excluded from the analysis due to overlaps in the study populations (Table S2). Including the same cases several times into one meta-analysis would lead to “double counting” if two studies are based on the same population but different details analyzed for example. Therefore it was deemed necessary to exclude those studies, which are based on mostly overlapping study populations. Only the study with a bigger study population and/or more detailed analyses was included.

#### Table B. Excluded studies due to overlaps in the study population with other studies.

| Study [reference] | Prefered study [reference] |
| --- | --- |
| Greenop et al. 2014 [62] | Milne et al. 2013 [21] |
| Milne et al. 2009 [63] | Milne et al. 2012 [51] |
| Milne et al. 2011 [64] | Milne et al. 2012 [51] |
| Reid et al. 2011 [65] | Milne et al. 2012 [51] |
| Bonaventure et al. 2012 [66] | Rudant et al. 2008 [5] |
| Bonaventure et al. 2013 [67] | Menegaux et al. 2007 [50] |
| Clavel et al. 2005 [68] | Rudant et al. 2008 [5] |
| Menegaux et al. 2005 [69] | Menegaux et al. 2007 [50] |
| Metayer et al. 2013 [70] | Oksuyan et al. 2006 [52] |
| Chang et al. 2006 [71] | Oksuyan et al. 2006 [52] |
| Kwan et al. 2009 [72] | Oksuyan et al. 2006 [52] |
| Barrington-Trimis et al. 2013 [73] | Norman et al. 1996 [22] |
| Winn et al. 1992 [74] | Holly et al. 1992 [28] |
| McLaughlin et al. 2006 [75] | Johnson et al. 2013 [36] |
| MacArthur et al. 2008 [76] | Infante-Rivard et al. 2000 [47] |
| Infante-Rivard et al. 2002 [77] | Infante-Rivard et al. 2000 [47] |
| Cnattingius et al. 1995 [78] | Mucci et al. 2004 [3] |
| Adami et al. 1996 [79] | Petridou et al. 2015 [4] |
| Schüz and Forman 2007 [80] | Schüz et al. 1999 [6] |
| Kaatsch et al. 1998 [81] | Schüz et al. 1999 [6] |
| Petridou et al. 2005 [82] | Lariou et al. 2013 [48] |
| Diamantaras et al. 2013 [83] | Lariou et al. 2013 [48] |
| Van Steensel-Moll et al. 1985 [84] | Van Duijn et al. 1994 [62] |
| Stavrou et al. 2009 [85] | McCredie et al. 1994; Milne et al. 2012 [19, 51] |
| Pershagen et al. 1992 [86] | Mucci et al. 2004; Petridou et al. 2015; Brooks et al. 2004; Linet et al. 1996 [3, 4, 7, 18] |

# S1.b. Study characterization

Overall, 432 datasets from 62 publications were included in this meta-analysis. To allow for biggest possible transparency in the conducted analysis detailed, technical details are presented in this supplemental material. The review table (see below), which lists all included studies, is presented. The datasets are numbered, so that each single line, which corresponds to one specific risk estimate from one specific study, can clearly be identified (column ERF ID). The identification numbers of the included datasets are listed in the results tables (Tables S5-S12.b).

#### Table C. In the meta-analysis included original studies maternal smoking during pregnancy and childhood cancer. All studies have case-control design as long as indicated otherwise (see definitions of acronyms and abbreviations at the end of the table).

| Study ID | ERF ID | Reference | Cancer type | Exposure time | Exposure amount (CPD) | OR (95% CI) | Cases (exposed/ nonexposed) | Controls (exposed/ nonexposed) | Cancer diagnosis year | Study country | Age at diagnoses (upper) [years] | Smoking data source |
| --- | --- | --- | --- | --- | --- | --- | --- | --- | --- | --- | --- | --- |
| 1 | 1.1 | Abadi-Korek et al. 2006 [41] | ALL | pregnancy | Any | 0.83 (0.35, 1.94) | 11/101 | 13/99 | 1984-2002 | Israel | not reported | Interview |
| 2 | 2.1 | Alexander et al. 2001 [42] | Acute Leukemia | pregnancy | Any | 1.43 (0.86, 2.39) | 36/100 | 53/213 | not reported | Multi | 0-1,5 y | Interview |
|  | 2.2 |  | ALL |  |  | 1.59 (0.82, 3.07) |  |  |  |  |  |  |
|  | 2.3 |  | AML |  |  | 1.33 (0.63, 2.8) |  |  |  |  |  |  |
| 3 | 3.1 | Brondum et al. 1999 [43] | ALL | 1 month bef pregnancy. | Any | 0.95 (0.74, 1.21) | not reported | not reported | 1989-1993 | US | 0-14 y | Interview |
|  | 3.2 |  |  | 1. trimester |  | 0.95 (0.74, 1.21) |  |  |  |  |  |  |
|  | 3.3 |  |  | 2. trimester |  | 0.95 (0.74, 1.21) |  |  |  |  |  |  |
|  | 3.4 |  |  | 3. trimester |  | 0.95 (0.74, 1.21) |  |  |  |  |  |  |
|  | 3.5 |  |  | breast feeding |  | 0.89 (0.66, 1.21) |  |  |  |  |  |  |
|  | 3.6 |  | AML | 1 month bef pregnancy | Any | 1.02 (0.87, 1.2) |  |  |  |  |  |  |
|  | 3.7 |  |  | 1. trimester |  | 0.96 (0.81, 1.14) |  |  |  |  |  |  |
|  | 3.8 |  |  | 2. trimester |  | 1.00 (0.83, 1.2) |  |  |  |  |  |  |
|  | 3.9 |  |  | 3. trimester |  | 0.99 (0.82, 1.19) |  |  |  |  |  |  |
|  | 3.10 |  |  | breast feeding |  | 0.96 (0.74, 1.25) |  |  |  |  |  |  |
| 4 | 4.1 | Brooks et al. 2004^†^ [7] | Nervous system tumor | pregnancy | Any | 1.24 (1.01, 1.53)^ | 144/336 | 347,392/693,343 | 1983-1997 | Sweden | not reported | Register |
|  | 4.2 |  |  |  | 1-9 | 1.22 (0.96, 1.56)^ | 88/336 | 214,778/693,343 |  |  |  |  |
|  | 4.3 |  |  |  | ≥ 10 | 1.29 (0.96, 1.73)^ | 56/336 | 131,173/693,343 |  |  |  |  |
|  | 4.4 |  | Malignant brain tumor |  | Any | 1.23 (0.98, 1.55)^ | 116/266 | 347,392/693,343 |  |  |  |  |
|  | 4.5 |  |  |  | 1-9 | 1.19 (0.91, 1.57)^ | 70/266 | 214,778/693,343 |  |  |  |  |
|  | 4.6 |  |  |  | ≥ 10 | 1.29 (0.93, 1.79)^ | 46/266 | 131,173/693,343 |  |  |  |  |
|  | 4.7 |  | Benign brain tumor |  | Any | 1.25 (0.73, 2.16)^ | 20/52 | 347,392/693,343 |  |  |  |  |
|  | 4.8 |  |  |  | 1-9 | 1.2 (0.63, 2.29)^ | 12/52 | 214,778/693,343 |  |  |  |  |
|  | 4.9 |  |  |  | ≥ 10 | 1.34 (0.62, 2.92)^ | 8/52 | 131,173/693,343 |  |  |  |  |
|  | 4.10 |  | Astrocytoma |  | Any | 1.37 (1.02, 1.85)^ | 72/148 | 347,392/693,343 |  |  |  |  |
|  | 4.11 |  |  |  | 1-9 | 1.34 (0.94, 1.9)^ | 44/148 | 214,778/693,343 |  |  |  |  |
|  | 4.12 |  |  |  | ≥ 10 | 1.44 (0.94, 2.19)^ | 28/148 | 131,173/693,343 |  |  |  |  |
|  | 4.13 |  | Medulloblastoma |  | Any | 1.17 (0.66, 2.09)^ | 18/44 | 347,392/693,343 |  |  |  |  |
|  | 4.14 |  |  |  | 1-9 | 1.16 (0.59, 2.29)^ | 11/44 | 214,778/693,343 |  |  |  |  |
|  | 4.15 |  |  |  | ≥ 10 | 1.19 (0.52, 2.73)^ | 7/44 | 131,173/693,343 |  |  |  |  |
|  | 4.16 |  | Ependymoma |  | Any | 0.94 (0.49, 1.81)^ | 14/37 | 347,392/693,343 |  |  |  |  |
|  | 4.17 |  |  |  | 1-9 | 0.68 (0.28, 1.63)^ | 6/37 | 214,778/693,343 |  |  |  |  |
|  | 4.18 |  |  |  | ≥ 10 | 1.37 (0.61, 3.07)^ | 8/37 | 131,173/693,343 |  |  |  |  |
| 5 | 5.1 | Buck et al. 2001 [8] | Neuroblastoma | pregnancy | Any | 1.4 (0.9, 2.1) | 50/61 | 85/140 | 1976-1987 | US | <6 y | Interview |
|  | 5.2 |  |  |  | 1-9 | 1.3 (0.9, 2) | 47/105 | 75/226 |  |  |  |  |
|  | 5.3 |  |  |  | ≥ 10 | 0.7 (0.2, 2.7) | 3/105 | 9/226 |  |  |  |  |
| 6 | 6.1 | Buckley et al. 1989 [34] | Hepatoblastoma | early pregnancy | 1-9 | 2.86 (0.71, 11.49) | 8/42 | 3/45 | 1980-1983 | US & Canada | 0-15 y | Interview |
|  | 6.2 |  |  |  | 10-19 | 0.75 (0.26, 2.15) | 7/42 | 10/45 |  |  |  |  |
|  | 6.3 |  |  |  | >20 | 0.95 (0.34, 2.69) | 8/42 | 9/45 |  |  |  |  |
|  | 6.4 |  |  |  | Any | 1.12 (0.54, 2.30) | 23/42 | 22/45 |  |  |  |  |
| 7 | 7.1 | Bunin et al. 1994 [9] | Astrocytoma | pregnancy | Any | 1.0 (0.6, 1.7) | 64/91 | 63/92 | 1986-1989 | US & Canada | 5 y | Interview |
|  | 7.2 |  | PNET |  |  | 1.0 (0.6, 1.7) | 60/106 | 58/108 |  |  |  |  |
|  | 7.3 |  | Nervous ystem tumor |  |  | 1.04 (0.76, 1.43) | 124/197 | 121/200 |  |  |  |  |
| 8 | 8.1 | Castro-Jiminez & Orozco-Vargas, 2011 [44] | ALL | pregnancy | Any | 1.18 (0.38, 3.67) | 7/78 | 6/79 | 2000-2005 | Colombia | 0-14 y | Interview |
| 9 | 9.1 | Chen et al. 2005 [37] | Malignant germ cell tumor | 1 month before pregnancy | Any | 1.0 (0.8, 1.4) | 96/192 | 126/320 | 1993-2001 | US | <15 y | Interview |
|  | 9.2 |  |  | 1. trimester |  | 1.1 (0.8, 1.5) | 84/192 | 104/320 |  |  |  |  |
|  | 9.3 |  |  | 2. trimester |  | 1.1 (0.7, 1.5) | 67/192 | 85/320 |  |  |  |  |
|  | 9.4 |  |  | 3. trimester |  | 1.0 (0.7, 1.5) | 61/192 | 80/320 |  |  |  |  |
|  | 9.5 |  |  | breast feeding |  | 0.8 (0.5, 1.3) | 49/192 | 96/320 |  |  |  |  |
|  | 9.6 |  |  | pregnancy | 1-9 | 1.1 (0.7, 1.5) | 47/192 | 58/320 |  |  |  |  |
|  | 9.7 |  |  |  | 10-19 | 1.2 (0.7, 1.9) | 20/192 | 24/320 |  |  |  |  |
|  | 9.8 |  |  |  | >20 | 1.1 (0.6, 2.1) | 15/192 | 19/320 |  |  |  |  |
|  | 9.9 |  |  |  | Any | 1.35 (0.96, 1.90) | 82/192 | 101/320 |  |  |  |  |
| 10 | 10.1 | Cordier et al. 1994 [10] | Nervous system tumor | pregnancy | Any | 1.6 (0.7, 3.5) | 19/56 | 23/90 | 1985-1987 | France | 15 y | Interview |
| 11 | 11.1 | de Fine Licht et al. 2012 [35] | Hepatoblastoma | pregnancy | Any | 1.0 (0.6, 1.8) | 17/75 | 95/414 | 1985-2006 | Multi | 0-14 y | Register |
| 12 | 12.1 | Farioli et al. 2014 [45] | ALL | 1. trimester | 1-10 | 1.05 (0.66, 1.65) | 55/330 | 84/530 | 1998-2003 | Italy | <11 y | Interview |
|  | 12.2 |  |  |  | >10 | 1.37 (0.53, 3.51) | 12/330 | 11/530 |  |  |  |  |
|  | 12.3 |  |  |  | Any | 1.13 (0.80, 1.59) | 67/330 | 95/530 |  |  |  |  |
| 13 | 13.1 | Ferreira et al. 2012 [46] | ALL | pregnancy | Any | 0.65 (0.31, 1.38) | 16/72 | 59/196 | 1999-2007 | Brazil | 0-11 months | Interview |
|  | 13.2 |  |  | 3 months before pregnancy |  | 1.23 (0.68, 2.22) | 29/59 | 79/176 |  |  |  |  |
|  | 13.3 |  |  | 1. trimester |  | 1.03 (0.49, 2.18) | 17/71 | 46/209 |  |  |  |  |
|  | 13.4 |  |  | 2. trimester |  | 0.93 (0.43, 2.02) | 12/76 | 45/210 |  |  |  |  |
|  | 13.5 |  |  | 3. trimester |  | 0.97 (0.45, 2.11) | 12/76 | 44/211 |  |  |  |  |
|  | 13.6 |  |  | breast feeding |  | 0.9 (0.42, 1.96) | 11/77 | 46/209 |  |  |  |  |
|  | 13.7 |  |  | pregnancy |  | 0.41 (0.11, 1.59) | 5/23 | 59/196 |  |  |  |  |
|  | 13.8 |  |  | 3 months before pregnancy |  | 0.82 (0.3, 2.25) | 8/20 | 79/176 |  |  |  |  |
|  | 13.9 |  |  | 1. trimester |  | 0.62 (0.16, 2.34) | 4/24 | 46/209 |  |  |  |  |
|  | 13.10 |  |  | 2. trimester |  | 0.38 (0.08, 1.83) | 3/25 | 45/210 |  |  |  |  |
|  | 13.11 |  |  | 3. trimester |  | 0.4 (0.08, 1.91) | 3/25 | 44/211 |  |  |  |  |
|  | 13.12 |  |  | breast feeding |  | 0.39 (0.08, 1.89) | 2/26 | 46/209 |  |  |  |  |
|  | 13.13 |  |  | pregnancy |  | 1.38 (0.65, 2.93) | 21/84 | 28/140 |  |  | 12-23 months |  |
|  | 13.14 |  |  | 3 months before pregnancy |  | 0.98 (0.5, 1.92) | 26/79 | 40/128 |  |  |  |  |
|  | 13.15 |  |  | 1. trimester |  | 1.61 (0.74, 3.51) | 21/84 | 23/145 |  |  |  |  |
|  | 13.16 |  |  | 2. trimester |  | 1.37 (0.61, 3.07) | 16/89 | 22/146 |  |  |  |  |
|  | 13.17 |  |  | 3. trimester |  | 1.5 (0.66, 3.41) | 16/89 | 22/146 |  |  |  |  |
|  | 13.18 |  |  | breast feeding |  | 1.86 (0.83, 4.2) | 17/88 | 20/148 |  |  |  |  |
|  | 13.19 |  |  | pregnancy |  | 0.46 (0.1, 2.24) | 2/29 | 28/140 |  |  |  |  |
|  | 13.20 |  |  | 3 months before pregnancy |  | 0.42 (0.13, 1.39) | 4/27 | 40/128 |  |  |  |  |
|  | 13.21 |  |  | 1. trimester |  | 0.41 (0.08, 2.09) | 2/29 | 23/145 |  |  |  |  |
|  | 13.22 |  |  | 2. trimester |  | 0.44 (0.09, 2.29) | 2/29 | 22/146 |  |  |  |  |
|  | 13.23 |  |  | 3. trimester |  | 0.49 (0.09, 2.57) | 2/29 | 22/146 |  |  |  |  |
|  | 13.24 |  |  | breast feeding |  | 0.69 (0.14, 3.53) | 2/29 | 20/148 |  |  |  |  |
|  | 13.25 |  |  | 3 months before pregnancy | 1-19 | 0.93 (0.59, 1.45) | 45/137 | 109/304 |  |  | <2 y |  |
|  | 13.26 |  |  |  | ≥20 | 2.52 (0.95, 6.72) | 11/137 | 10/304 |  |  |  |  |
|  | 13.27 |  |  | 1. trimester | 1-19 | 1.1 (0.65, 1.82) | 33/154 | 65/354 |  |  |  |  |
|  | 13.28 |  |  |  | ≥20 | 3.35 (0.75, 15) | 6/154 | 4/354 |  |  |  |  |
|  | 13.29 |  |  | 2. trimester | 1-19 | 0.47 (0.02, 0.92) | 23/164 | 65/356 |  |  |  |  |
|  | 13.30 |  |  |  | ≥20 | 6.82 (1.09, 4.3) | 6/164 | 2/356 |  |  |  |  |
|  | 13.31 |  |  | 3. trimester | 1-19 | 0.96 (0.54, 1.69) | 22/165 | 64/357 |  |  |  |  |
|  | 13.32 |  |  |  | ≥20 | 6.81 (1.09, 42.7) | 6/165 | 2/357 |  |  |  |  |
|  | 13.33 |  |  | breast feeding | 1-19 | 0.94 (0.53, 1.66) | 22/164 | 64/357 |  |  |  |  |
|  | 13.34 |  |  |  | ≥20 | 7.78 (1.33, 45.5) | 7/164 | 2/357 |  |  |  |  |
|  | 13.35 |  | AML | 3 months before pregnancy | 1-19 | 0.59 (0.27, 1.28) | 11/47 | 109/304 |  |  | <2 y |  |
|  | 13.36 |  |  |  | ≥20 | 0.8 (0.93, 6.9) | 1/47 | 10/304 |  |  |  |  |
|  | 13.37 |  |  | 1. trimester | 1-19 | 0.44 (0.15, 1.31) | 5/53 | 65/354 |  |  |  |  |
|  | 13.38 |  |  |  | ≥20 | 2.7 (0.27, 26.5) | 1/53 | 4/354 |  |  |  |  |
|  | 13.39 |  |  | 2. trimester | 1-19 | 0.17 (0.01, 2.14) | 4/54 | 65/356 |  |  |  |  |
|  | 13.40 |  |  |  | ≥20 | 5.97 (0.47, 76) | 1/54 | 2/356 |  |  |  |  |
|  | 13.41 |  |  | 3. trimester | 1-19 | 0.43 (0.1, 1.18) | 4/54 | 64/357 |  |  |  |  |
|  | 13.42 |  |  |  | ≥20 | 6.03 (0.47, 77) | 1/54 | 2/357 |  |  |  |  |
|  | 13.43 |  |  | breast feeding | 1-19 | 0.37 (0.11, 1.28) | 3/55 | 64/357 |  |  |  |  |
|  | 13.44 |  |  |  | ≥20 | 6.05 (0.47, 77) | 1/55 | 2/357 |  |  |  |  |
|  | 13.45 |  | Acute Leukemia | pregnancy | Any | 0.73 (0.44, 1.23) | 21/95 | 118/392 |  |  | 0-11 months |  |
|  | 13.46 |  |  | 3 months before pregnancy |  | 1.04 (0.68, 1.61) | 37/79 | 158/352 |  |  |  |  |
|  | 13.47 |  |  | 1. trimester |  | 1.00 (0.59, 1.69) | 21/95 | 92/418 |  |  |  |  |
|  | 13.48 |  |  | 2. trimester |  | 0.69 (0.385, 1.25) | 15/101 | 90/420 |  |  |  |  |
|  | 13.49 |  |  | 3. trimester |  | 0.71 (0.39, 1.28) | 15/101 | 88/422 |  |  |  |  |
|  | 13.50 |  |  | breast feeding |  | 0.57 (0.31, 1.07) | 13/103 | 92/418 |  |  |  |  |
|  | 13.51 |  |  | 3 months before pregnancy | 1-19 | 0.85 (0.61, 1.19) | 56/184 | 218/608 |  |  | <2 y |  |
|  | 13.52 |  |  |  | ≥20 | 1.98 (0.95, 4.13) | 12/184 | 20/608 |  |  |  |  |
|  | 13.53 |  |  | 1. trimester | 1-19 | 0.99 (0.67, 1.48) | 38/207 | 130/708 |  |  |  |  |
|  | 13.54 |  |  |  | ≥20 | 2.99 (1.07, 8.35) | 7/207 | 8/708 |  |  |  |  |
|  | 13.55 |  |  | 2. trimester | 1-19 | 0.68 (0.43, 1.05) | 27/218 | 130/712 |  |  |  |  |
|  | 13.56 |  |  |  | ≥20 | 5.72 (1.66, 19.71) | 7/218 | 4/712 |  |  |  |  |
|  | 13.57 |  |  | 3. trimester | 1-19 | 0.66 (0.42, 1.04) | 26/219 | 128/714 |  |  |  |  |
|  | 13.58 |  |  |  | ≥20 | 5.71 (1.65, 19.67) | 7/219 | 4/714 |  |  |  |  |
|  | 13.59 |  |  | breast feeding | 1-19 | 0.64 (0.40, 1.00) | 25/219 | 128/714 |  |  |  |  |
|  | 13.60 |  |  |  | ≥20 | 6.52 (1.94, 21.87) | 8/219 | 4/714 |  |  |  |  |
| 14 | 14.1 | Filippini et al. 1994 [11] | Nervous system tumor | pregnancy | Any | 1.6 (0.7, 3.7) | 18/73 | 59/262 | 1985-1988 | Italy | 15 y | Question-naire |
|  | 14.2 |  |  |  | 1-10 | 1.6 (0.7, 3.8) | 14/73 | 48/262 |  |  |  |  |
|  | 14.3 |  |  |  | >10 | 1.7 (0.4, 6.6) | 4/73 | 11/262 |  |  |  |  |
| 15 | 15.1 | Filippini et al. 2000 [12] | Nervous system tumor | before knowledge (1st 5 weeks) | Any | 1.5 (1.0, 2.3) | 83/161 | 160/342 | 1988-1993 | Italy | 15 y | Interview |
|  | 15.2 |  |  |  | 1-10 | 1.5 (1.0, 2.32) | 57/161 | 114/342 |  |  |  |  |
|  | 15.3 |  |  |  | >10 | 1.7 (0.9, 2.9) | 26/161 | 46/342 |  |  |  |  |
|  | 15.4 |  |  | after knowledge (>5 weeks) | Any | 1.4 (0.8, 2.4) | 31/140 | 63/465 |  |  |  |  |
|  | 15.5 |  |  |  | 1-10 | 1.4 (0.8, 2.5) | 27/140 | 56/465 |  |  |  |  |
|  | 15.6 |  |  |  | >10 | 1.7 (0.5, 6) | 4/140 | 7/465 |  |  |  |  |
| 16 | 16.1 | Filippini et al. 2002 [13] | Nervous system tumor | pregnancy | Any | 0.9 (0.8, 1.1) | 210/1006 | 422/1800 | 1980-1992 | Multi | <15/16/19 y | Interview |
|  | 16.2 |  | Astroglial tumor |  |  | 1.0 (0.8, 1.2) | 113/510 | 422/1800 |  |  |  |  |
|  | 16.3 |  | PNET |  |  | 0.8 (0.6, 1.2) | 41/217 | 422/1800 |  |  |  |  |
|  | 16.4 |  | other glial tumor |  |  | 1.2 (0.8, 1.8) | 31/116 | 422/1800 |  |  |  |  |
|  | 16.5 |  | Other brain tumor (not glial) |  |  | 0.7 (0.4, 1.1) | 24/155 | 422/1800 |  |  |  |  |
| 17 | 17.1 | Gold et al. 1993 [14] | Astrocytoma | pregnancy | 1-9 | 0.71 (0.36, 1.39) | 20/93 | 63/279 | 1977-1981 | US | 0-17 y | Interview |
|  | 17.2 |  |  |  | ≥ 10 | 1.08 (0.65, 1.79) | 35/93 | 111/279 |  |  |  |  |
|  | 17.3 |  |  |  | Any | 0.95 (0.65, 1.39) | 55/93 | 174/279 |  |  |  |  |
|  | 17.4 |  | Medulloblastoma |  | 1-9 | 1.04 (0.4, 2.71) | 9/36 | 22/103 |  |  |  |  |
|  | 17.5 |  |  |  | ≥ 10 | 1.08 (0.46, 2.56) | 11/36 | 34/103 |  |  |  |  |
|  | 17.6 |  |  |  | Any | 1.02 (0.54, 1.93) | 20/36 | 56/103 |  |  |  |  |
|  | 17.7 |  | Nervous system tumor |  | 1-9 | 0.97 (0.673, 1.39) | 49/204 | 145/583 |  |  |  |  |
|  | 17.8 |  |  |  | ≥ 10 | 0.91 (0.66, 1.24) | 69/204 | 217/583 |  |  |  |  |
|  | 17.9 |  |  |  | Any | 0.93 (0.72, 1.21) | 118/204 | 362/583 |  |  |  |  |
| 18 | 18.1 | Grufferman et al. 1982 [29] | Rhabdomyo- sarcoma | pregnancy | Any | 1.0 (0.4, 2.4)* | 9/24 | 27/72 | 1967-1976 | US | 0-14 y | Interview |
| 19 | 19.1 | Grufferman et al. 1993 [30] | Soft tissue sarcoma | pregnancy | Any | 1.0 (0.7, 1.4) | 117/205 | 116/206 | 1982-1988 | US | 0-20 y | Interview |
| 20 | 20.1 | Holly et al. 1992 [28] | Ewing's sarcoma | pregnancy | Any | 1.1 (0.5, 2.4)* | 13/30 | 47/146 | 1978-1986 | US | unclear | Interview |
| 21 | 21.1 | Howe et al. 1989 [15] | Nervous system tumor | pregnancy | Any | 1.42 (0.7, 3)* | 18/56 | 32/106 | 1977-1983 | Canada | 19 y | Interview |
| 22 | 22.1 | Hu et al. 2000 [16] | Nervous system tumor | pregnancy | Any | 1.2 (0.45, 3.23) | 9/73 | 18/228 | 1991-1996 | China | 0-18 y | Interview |
| 23 | 23.1 | Infante-Rivard et al. 2000 [47] | ALL | 1. trimester | 1-20 | 1.1 (0.8, 1.6) | 136/303 | 123/316 | 1980-1993 | Canada | <11 y | Question-naire |
|  | 23.2 |  |  |  | >20 | 1.0 (0.7, 1.6) | 52/303 | 52/316 |  |  |  |  |
|  | 23.3 |  |  |  | Any | 1.12 (0.86, 1.45) | 188/303 | 175/316 |  |  |  |  |
|  | 23.4 |  |  | 2. trimester | 1-20 | 1.2 (0.8, 1.6) | 128/320 | 116/336 |  |  |  |  |
|  | 23.5 |  |  |  | >20 | 1.2 (0.7, 1.9) | 43/320 | 38/336 |  |  |  |  |
|  | 23.6 |  |  |  | Any | 1.17 (0.89, 1.52) | 171/320 | 154/336 |  |  |  |  |
|  | 23.7 |  |  | 3. trimester | 1-20 | 1.2 (0.8, 1.6) | 127/321 | 115/339 |  |  |  |  |
|  | 23.8 |  |  |  | >20 | 1.2 (0.8, 2) | 43/321 | 36/339 |  |  |  |  |
|  | 23.9 |  |  |  | Any | 1.19 (0.91, 1.55) | 170/321 | 151/339 |  |  |  |  |
| 24 | 24.1 | John et al. 1991 [1] | All cancer combined | 3 months before pregnancy | Any | 1.3 (0.8, 2.0) | 80/82 | 65/86 | 1976-1983 | US | 0-14 y | Interview |
|  | 24.2 |  |  |  | 1-10 | 1.3 (0.7, 2.4) | 32/82 | 65/86 |  |  |  |  |
|  | 24.3 |  |  |  | >10 | 1.3 (0.8, 2.2) | 48/82 | 65/86 |  |  |  |  |
|  | 24.4 |  | ALL |  | Any | 2.1 (1.0, 4.3) | 24/15 | 65/86 |  |  |  |  |
|  | 24.5 |  |  |  | 1-10 | 2.0 (0.8, 5.0) | 9/15 | 65/86 |  |  |  |  |
|  | 24.6 |  |  |  | >10 | 2.2 (1.4. 2.9) | 15/15 | 65/86 |  |  |  |  |
|  | 24.7 |  | Lymphoma |  | Any | 1.9 (0.7, 5.2) | 10/7 | 65/86 |  |  |  |  |
|  | 24.8 |  | Nervous system tumor |  | Any | 0.9 (0.4, 2.1) | 11/16 | 65/86 |  |  |  |  |
|  | 24.9 |  | Soft tissue sarcoma |  | Any | 1.2 (0.5, 3.0) | 10/11 | 65/86 |  |  |  |  |
|  | 24.10 |  | All cancer combined | 1. trimester | Any | 1.5 (1.0, 2.5) | 65/82 | 44/86 |  |  |  |  |
|  | 24.11 |  |  |  | 1-10 | 1.3 (0.7, 2.7) | 23/82 | 44/86 |  |  |  |  |
|  | 24.12 |  |  |  | >10 | 1.7 (1, 3) | 42/82 | 44/86 |  |  |  |  |
|  | 24.13 |  | ALL |  | Any | 2.3 (1.1, 5.0) | 18/15 | 44/86 |  |  |  |  |
|  | 24.14 |  |  |  | 1-10 | 1.9 (0.7, 5.6) | 6/15 | 44/86 |  |  |  |  |
|  | 24.15 |  |  |  | >10 | 2.6 (1.1, 6.3) | 12/15 | 44/86 |  |  |  |  |
|  | 24.16 |  | Lymphoma |  | Any | 2.5 (0.9, 7) | 9/7 | 44/86 |  |  |  |  |
|  | 24.17 |  | Nervous system tumor |  | Any | 1.0 (0.4, 2.5) | 8/16 | 44/86 |  |  |  |  |
|  | 24.18 |  | Soft tissue sarcoma |  | Any | 1.2 (0.5, 3.4) | 7/11 | 44/86 |  |  |  |  |
|  | 24.19 |  | All cancer combined | pregnancy | Any | 1.4 (0.9, 2.4) | 56/82 | 41/86 |  |  |  |  |
|  | 24.20 |  |  |  | 1-10 | 1.4 (0.7, 2.7) | 22/82 | 41/86 |  |  |  |  |
|  | 24.21 |  |  |  | >10 | 1.5 (0.8, 2.7) | 34/82 | 41/86 |  |  |  |  |
|  | 24.22 |  | ALL |  | Any | 2.5 (1.2, 5.4) | 18/15 | 41/86 |  |  |  |  |
|  | 24.23 |  |  |  | 1-10 | 2.0 (0.7, 5.9) | 6/15 | 41/86 |  |  |  |  |
|  | 24.24 |  |  |  | >10 | 2.9 (1.2, 6.8) | 12/15 | 41/86 |  |  |  |  |
|  | 24.25 |  | Lymphoma |  | Any | 2.7 (1.0, 7.6) | 9/7 | 41/86 |  |  |  |  |
|  | 24.26 |  | Nervous system tumor |  | Any | 0.8 (0.3, 2.2) | 6/16 | 41/86 |  |  |  |  |
|  | 24.27 |  | Soft tissue sarcoma |  | Any | 1.3 (0.5, 3.7) | 7/11 | 41/86 |  |  |  |  |
| 25 | 25.1 | Johnson et al. 2013 [36] | Hepatoblastoma | pregnancy | Any | 0.9 (0.5, 1.6) | 34/344 | 34/344 | 2000-2008 | US | <6 y | Interview |
| 26 | 26.1 | Kramer et al. 1987 [17] | Neuroblastoma | pregnancy | Any | 1.26 (0.76, 2.09) | / | / | 1970-1979 | US |  | Interview |
| 27 | 27.1 | Lariou et al. 2013 [48] | ALL | pregnancy | Any | 1.32 (0.83, 2.1) | 49/203 | 45/249 | 1999-2003 | Greece | <15 y | Interview |
| 28 | 28.1 | Linet et al. 1996 [18] | Astrocytoma | pregnancy | 1-9 | 1.21 (0.51, 2.87) | 8/31 | 33/155 | 1982-1989 | Sweden | 0-14 y | Register |
|  | 28.2 |  |  |  | ≥ 10 | 0.48 (0.15, 2.14) | 2/31 | 21/155 |  |  |  |  |
|  | 28.3 |  |  |  | Any | 0.93(0.43, 2.01) | 10/31 | 54/155 |  |  |  |  |
|  | 28.4 |  | Medulloblastoma |  | 1-9 | 0.8 (0.2, 3.3) | 3/11 | 18/54 |  |  |  |  |
|  | 28.5 |  |  |  | ≥ 10 | 1.7 (0.4, 7.2) | 3/11 | 9/54 |  |  |  |  |
|  | 28.6 |  |  |  | Any | 1.09 (0.36, 3.267) | 6/11 | 27/54 |  |  |  |  |
|  | 28.7 |  | Ependymoma |  | 1-9 | 1.2 (0.3, 5.1) | 3/9 | 14/48 |  |  |  |  |
|  | 28.8 |  |  |  | ≥ 10 | 1.5 (0.3, 8.2) | 2/9 | 7/48 |  |  |  |  |
|  | 28.9 |  |  |  | Any | 1.27 (0.38, 4.25) | 5/9 | 21/48 |  |  |  |  |
|  | 28.10 |  | Nervous system tumor |  | 1-9 | 1.3 (0.7, 2.2) | 19/67 | 80/353 |  |  |  |  |
|  | 28.11 |  |  |  | ≥ 10 | 1.0 (0.5, 2.1) | 10/67 | 51/353 |  |  |  |  |
|  | 28.12 |  |  |  | Any | 1.17 (0.72, 1.88) | 29/67 | 131/353 |  |  |  |  |
| 29 | 29.1 | Magnani et al. 1990 [2] | ALL | pregnancy | Any | 0.7 (0.5, 1.1) | 40/102 | 102/205 | 1974-1984 | Italy | 0-14 y | Interview |
|  | 29.2 |  |  |  | 1-15 | 0.6 (0.4, 1.0) | 30/102 | 87/205 |  |  |  |  |
|  | 29.3 |  |  |  | >15 | 1.0 (0.4, 2.7) | 8/102 | 13/205 |  |  |  |  |
|  | 29.4 |  | Acute non-lymphoid leukemia |  | Any | 2.0 (0.8, 4.8) | 10/12 | 102/205 |  |  |  |  |
|  | 29.5 |  | Acute Leukemia |  |  | 0.88 (0.59, 1.32) | 50/114 | 102/205 |  |  |  |  |
|  | 29.6 |  | Non-Hodgkin Lymphoma |  |  | 1.7 (0.7, 4.5) | 9/10 | 102/205 |  |  |  |  |
| 30 | 30.1 | Magnani et al. 1989 [31] | Soft tissue sarcoma | pregnancy | 1-15 | 0.7 (0.3, 1.4) | 12/40 | 94/215 | 1983-1984 | Italy | 0-14 y | Interview |
|  | 30.2 |  |  |  | Any | 0.58 (0.29, 1.15) | 12/40 | 111/215 |  |  |  |  |
|  | 30.3 |  | Rhabdomyo- sarcoma |  | 1-15 | 1.0 (0.4, 2.3) | 11/25 | 94/215 |  |  |  |  |
|  | 30.4 |  |  |  | Any | 0.85 (0.40, 1.79) | 11/25 | 111/215 |  |  |  |  |
| 31 | 31.1 | Mattioli et al. 2014 [49] | Acute non-lymphoid leukemia | 1. trimester | Any | 1.35 (0.68, 2.66) | 11/71 | 14/115 | 1998-2003 | Italy | 0-10 y | Interview |
| 32 | 32.1 | McCredie et al. 1994 [19] | Nervous system tumor | pregnancy | Any | 0.9 (0.5, 1.8) | 22/54 | 34/108 | 1987-1987 | UK | 0-14 y | Interview |
| 33 | 33.1 | Menegaux et al. 2007 [50] | Acute Leukemia | pregnancy | Any | 1.3 (0.9, 1.7) | 98/372 | 97/463 | 1995-1998 | France | <15 y | Question-naire |
|  | 33.2 |  |  |  | ≤5 | 1.1 (0.7, 1.7) | 50/372 | 58/463 |  |  |  |  |
|  | 33.3 |  |  |  | >5 | 1.4 (0.9, 2.2) | 44/372 | 37/463 |  |  |  |  |
|  | 33.4 |  | ALL |  | Any | 1.4 (39814) | 90/315 | 97/463 |  |  |  |  |
|  | 33.5 |  |  |  | ≤5 | 1.3 (0.9, 2) | 48/315 | 58/463 |  |  |  |  |
|  | 33.6 |  |  |  | >5 | 1.5 (0.9, 2.5) | 41/315 | 37/463 |  |  |  |  |
|  | 33.7 |  | AML |  | Any | 0.6 (0.3, 1.5) | 8/54 | 97/463 |  |  |  |  |
|  | 33.8 |  |  |  | ≤5 | 0.2 (0.04, 1) | 2/54 | 58/463 |  |  |  |  |
|  | 33.9 |  |  |  | >5 | 0.7 (0.2, 2.6) | 3/54 | 37/463 |  |  |  |  |
| 34 | 34.1 | Michaelis et al. 2000 [20] | Nervous system tumor | pregnancy | 1-10 | 0.77 (0.57, 1.04) | 66/376 | 416/1944 | 1980-1997 | Germany | 0-14 y | Question-naire |
|  | 34.2 |  |  |  | 11-20 | 1.45 (0.83, 2.55) | 17/376 | 66/1944 |  |  |  |  |
|  | 34.3 |  |  |  | >20 | 0.82 (0.18, 3.76) | 2/376 | 12/1944 |  |  |  |  |
|  | 34.5 |  |  |  | Any | 1.48 (1.13, 1.94) | 85/226 | 494/1944 |  |  |  |  |
| 35 | 35.1 | Milne et al. 2012 [51] | ALL | pregnancy | Any | 1.02 (0.76, 1.37) | 98/290 | 199/669 | 2003-2006 | Australia | <15 y | Question-naire |
|  | 35.2 |  |  |  | 1-14 | 1.07 (0.73, 1.58) | 49/290 | 92/669 |  |  |  |  |
|  | 35.3 |  |  |  | >14 | 0.96 (0.67, 1.44) | 49/290 | 107/669 |  |  |  |  |
| 36 | 36.1 | Milne et al. 2013 [21] | Nervous system tumor | pregnancy | Any | 0.89 (0.61, 1.31) | 72/228 | 206/731 | 2005-2010 | Australia | <15 y | Question-naire |
|  | 36.2 |  |  |  | 1-14 | 0.91 (0.56, 1.47) | 41/228 | 101/731 |  |  |  |  |
|  | 36.3 |  |  |  | >14 | 0.88 (0.52, 1.49) | 31/228 | 105/731 |  |  |  |  |
| 37 | 37.1 | Mucci et al. 2004^†^ [3] | ALL | pregnancy | Any | 0.75 (0.6, 0.93)^ | 105/400 | 345,735/ 1,094,112 | 1983-1997 | Sweden | <6 y | Register |
|  | 37.2 |  |  |  | 1-9 | 0.69 (0.52, 0.91)^ | 61/400 | 345,735/ 1,094,112 |  |  |  |  |
|  | 37.3 |  |  |  | >10 | 0.84 (0.61, 1.15)^ | 44/400 | 345,735/ 1,094,112 |  |  |  |  |
|  | 37.4 |  | AML |  | Any | 1.28 (0.65, 2.49)^ | 15/33 | 345,735/ 1,094,112 |  |  |  |  |
|  | 37.5 |  |  |  | 1-9 | 0.75 (0.29, 1.96)^ | 6/33 | 345,735/ 1,094,112 |  |  |  |  |
|  | 37.6 |  |  |  | >10 | 2.2 (1.0, 4.83)^ | 9/33 | 345,735/ 1,094,112 |  |  |  |  |
|  | 37.7 |  | Acute Leukemia |  | Any | 0.88 (0.72, 1.07^) | 120/433 | 345,735/ 1,094,112 |  |  |  |  |
|  | 37.8 |  |  |  | 1-9 | 0.49 (0.38, 0.63)^ | 67/433 | 345,735/ 1,094,112 |  |  |  |  |
|  | 37.9 |  |  |  | >10 | 0.39 (0.29, 0.52)^ | 53/433 | 345,735/ 1,094,112 |  |  |  |  |
|  | 37.10 |  | Non-Hodgkin Lymphoma |  | Any | 1.22 (0.74, 2.02)^ | 25/56 | 345,735/ 1,094,112 |  |  |  |  |
|  | 37.11 |  |  |  | 1-9 | 1.15 (0.63, 2.11)^ | 15/56 | 345,735/ 1,094,112 |  |  |  |  |
|  | 37.12 |  |  |  | >10 | 1.33 (0.66, 2.68)^ | 10/56 | 345,735/ 1,094,112 |  |  |  |  |
|  | 37.13 |  | Reticulosis |  | Any | 1.12 (0.61, 2.05)^ | 17/44 | 345,735/ 1,094,112 |  |  |  |  |
|  | 37.14 |  |  |  | 1-9 | 1.47 (0.77, 2.79)^ | 14/44 | 345,735/ 1,094,112 |  |  |  |  |
|  | 37.15 |  |  |  | >10 | 0.54 (0.16, 1.77)^ | 13/44 | 345,735/ 1,094,112 |  |  |  |  |
| 38 | 38.1 | Norman et al. 1996 [22] | Nervous system tumor | 1. trimester | Any | 0.98 (0.72, 1.3) | 87/241 | 144/387 | 1984-1991 | US | 19 y | Interview |
|  | 38.2 |  |  | 2. trimester | Any | 0.92 (0.66, 1.3) | 72/241 | 128/387 |  |  |  |  |
|  | 38.3 |  |  | 3. trimester | Any | 0.97 (0.69, 1.4) | 73/241 | 125/387 |  |  |  |  |
|  | 38.4 |  |  | pregnancy | 1-10 | 0.98 (0.67, 1.4) | 54/241 | 89/387 |  |  |  |  |
|  | 38.5 |  |  |  | ≥ 10 | 0.92 (0.58, 1.4) | 35/241 | 62/387 |  |  |  |  |
|  | 38.6 |  |  |  | Any | 0.98 (0.72, 1.3) | 92/241 | 152/387 |  |  |  |  |
| 39 | 39.1 | Oksuzyan et al. 2012 [52] | ALL | pregnancy | Any | 0.62 (0.44, 0.89) | 49/5725 | 78/5694 | 1988-2008 | US | 0-16 y | Register |
|  | 39.2 |  | AML |  |  | 0.87 (0.42, 1.78) | 14/5725 | 16/5694 |  |  |  |  |
|  | 39.3 |  | Acute Leukemia |  |  | 0.67 (0.48, 0.92) | 63/5725 | 94/5694 |  |  |  |  |
| 40 | 40.1 | Olshan et al. 1993 [32] | Wilms’ tumor | pregnancy | 1-9 | 0.79 (0.35, 1.81) | 12/154 | 15/180 | 1984-1986 | US | 0-15 y | Interview |
|  | 40.2 |  |  |  | > 9 | 0.73 (0.4, 1.34) | 29/154 | 37/180 |  |  |  |  |
|  | 40.3 |  |  |  | Any | 0.92 (0.58, 1.46) | 41/154 | 52/180 |  |  |  |  |
| 41 | 41.1 | Orsi et al. 2015 [53] | Acute Leukemia | pregnancy | Any | 1.0 (0.8, 1.2) | 158/588 | 290/1129 | 2010-2011 | France | <15 y | Interview |
|  | 41.2 |  |  |  | <5 | 1.0 (0.7, 1.3) | 79/588 | 146/1129 |  |  |  |  |
|  | 41.3 |  |  |  | 5-10 | 1.0 (0.7, 1.4) | 64/588 | 115/1129 |  |  |  |  |
|  | 41.4 |  |  |  | 1-10 | 1.05 (0.84, 1.32) | 143/588 | 261/1129 |  |  |  |  |
|  | 41.5 |  |  |  | ≥11 | 1.0 (0.5, 1.9) | 15/588 | 29/1129 |  |  |  |  |
|  | 41.6 |  |  | 1. trimester | Any | 1.0 (0.8, 1.3) | 154/588 | 279/1129 |  |  |  |  |
|  | 41.7 |  |  | 2. trimester |  | 1.0 (0.8, 1.2) | 129/588 | 244/1129 |  |  |  |  |
|  | 41.8 |  |  | 3. trimester |  | 1.0 (0.8, 1.3) | 130/588 | 239/1129 |  |  |  |  |
|  | 41.9 |  | ALL | pregnancy | Any | 1.0 (0.8, 1.3) | 136/499 | 290/1129 |  |  |  |  |
|  | 41.10 |  |  |  | <5 | 1.0 (0.7, 1.3) | 68/499 | 146/1129 |  |  |  |  |
|  | 41.11 |  |  |  | 5-10 | 1.0 (0.7, 1.5) | 55/499 | 115/1129 |  |  |  |  |
|  | 41.12 |  |  |  | 1-10 | 1.07 (0.84, 1.35) | 123/499 | 261/1129 |  |  |  |  |
|  | 41.13 |  |  |  | ≥11 | 1.0 (0.5, 2.0) | 13/499 | 29/1129 |  |  |  |  |
|  | 41.14 |  |  | 1. trimester | Any | 1.0 (0.8, 1.3) | 132/499 | 279/1129 |  |  |  |  |
|  | 41.15 |  |  | 2. trimester |  | 1.0 (0.8, 1.3) | 112/499 | 244/1129 |  |  |  |  |
|  | 41.16 |  |  | 3. trimester |  | 1.0 (0.8, 1.3) | 113/499 | 239/1129 |  |  |  |  |
|  | 41.17 |  | AML | pregnancy | Any | 0.9 (0.5, 1.5) | 18/82 | 290/1129 |  |  |  |  |
|  | 41.18 |  |  |  | <5 | 1.0 (0.5, 1.9) | 10/82 | 146/1129 |  |  |  |  |
|  | 41.19 |  |  |  | 5-10 | 0.7 (0.3, 1.7) | 6/82 | 115/1129 |  |  |  |  |
|  | 41.20 |  |  |  | 1-10 | 0.84 (0.49, 1.47) | 16/82 | 261/1129 |  |  |  |  |
|  | 41.21 |  |  |  | ≥11 | 1.0 (0.2, 4.1) | 2/82 | 29/1129 |  |  |  |  |
|  | 41.22 |  |  | 1. trimester | Any | 0.9 (0.5, 1.6) | 18/82 | 279/1129 |  |  |  |  |
|  | 41.23 |  |  | 2. trimester |  | 0.8 (0.4, 1.5) | 14/82 | 244/1129 |  |  |  |  |
|  | 41.24 |  |  | 3. trimester |  | 0.8 (0.5, 1.5) | 14/82 | 239/1129 |  |  |  |  |
| 42 | 42.1 | Petridou et al. 1997 [54] | Leukemia | pregnancy | Any | 1.19 (0.73, 1.93) | 77/76 | 146/154 | 1953-1955 | Greece | <15 y | Interview |
| 43 | 43.1 | Petridou et al. 2015^†^ [4] | Non-Hodgkin Lymphoma | early pregnancy | Any | 1.15 (0.89, 1.48)^ | 85/255 | 44,9974/ 1,914,254 | 1983-2007 | Sweden | <15 y | Register |
|  | 43.2 |  | Hodgkin Lymphoma |  |  | 0.99 (0.6, 1.61)^ | 23/64 | 450,036/ 1,914,445 |  |  |  |  |
| 44 | 44.1 | Pettersson et al. 2007 [38] | Germ cell tumor | pregnancy | Any | 0.91 (0.64, 1.3) | 81/111 | 221/273 | 1973-2002 | Sweden | <15 y | Register |
|  | 44.2 |  | Semimoma |  | 1-9 | 0.29 (0.09, 0.99) | 4/34 | 24/69 |  |  |  |  |
|  | 44.3 |  |  |  | >10 | 0.49 (0.17, 1.43) | 6/34 | 23/69 |  |  |  |  |
|  | 44.4 |  | Nonsemimoma |  | 1-9 | 1.0 (0.6, 1.66) | 30/77 | 78/204 |  |  |  |  |
|  | 44.5 |  |  |  | >10 | 1.18 (0.73, 1.91) | 41/77 | 96/204 |  |  |  |  |
| 45 | 45.1 | Plichart et al. 2008 [23] | Nervous system tumor | pregnancy | Any | 1.1 (0.8, 1.6) | 44/165 | 325/1356 | 2003-2004 | France | <15 y | Interview |
|  | 45.2 |  |  |  | <11 | 1.1 (0.7, 1.6) | 29/165 | 220/1356 |  |  |  |  |
|  | 45.3 |  |  |  | ≥10 | 1.1 (0.6, 2) | 13/165 | 99/1356 |  |  |  |  |
|  | 45.4 |  | PNET |  | Any | 1.1 (0.7, 1.8) | 21/79 | 325/1356 |  |  |  |  |
|  | 45.5 |  |  |  | <11 | 1.2 (0.7, 2.1) | 16/79 | 220/1356 |  |  |  |  |
|  | 45.6 |  |  |  | ≥10 | 0.9 (0.3, 2.2) | 5/79 | 99/1356 |  |  |  |  |
|  | 45.7 |  | Ependymoma |  | Any | 1.0 (0.4, 2.4) | 6/27 | 325/1356 |  |  |  |  |
|  | 45.8 |  |  |  | <11 | 0.9 (0.3, 2.7) | 4/27 | 220/1356 |  |  |  |  |
|  | 45.9 |  |  |  | ≥10 | 0.6 (0.1, 4.3) | 1/27 | 99/1356 |  |  |  |  |
|  | 45.10 |  | Astrocytoma |  | Any | 1.3 (0.5, 3.2) | 6/20 | 325/1356 |  |  |  |  |
|  | 45.11 |  |  |  | <11 | 1.3 (0.4, 3.8) | 4/20 | 220/1356 |  |  |  |  |
|  | 45.12 |  |  |  | ≥10 | 1.4 (0.3, 5.9) | 2/20 | 99/1356 |  |  |  |  |
|  | 45.13 |  | Other glial tumor |  | Any | 1.2 (0.6, 2.4) | 10/35 | 325/1356 |  |  |  |  |
|  | 45.14 |  |  |  | <11 | 0.9 (0.3, 2.3) | 5/35 | 220/1356 |  |  |  |  |
|  | 45.15 |  |  |  | ≥10 | 1.6 (0.5, 4.5) | 4/35 | 99/1356 |  |  |  |  |
| 46 | 46.1 | Podvin et al. 2006 [55] | Leukemia | pregnancy | Any | 0.8 (0.7, 1.1) | 70/525 | 5127/823 | 1984-2002 | US | <21 y | Register |
|  | 46.2 |  | ALL |  |  | 0.8 (0.6, 1.2) | 65/311 | 981/3999 |  |  |  |  |
|  | 46.3 |  | AML |  |  | 0.8 (0.4, 1.6) | 13/72 | 981/3999 |  |  |  |  |
| 47 | 47.1 | Preston-Martin et al. 1982 [24] | Nervous system tumor | pregnancy | Any | 1.06 (0.72, 1.58) | 82/127 | 79/130 | 1972-1977 | US | 0-24 y | Interview |
| 48 | 48.1 | Rosenbaum et al. 2005 [56] | ALL | pregnancy | Any | 0.87 (0.65, 1.19) | 69/186 | 227/533 | 1980-1991 | US | <15 y | Question-naire |
| 49 | 49.1 | Rudant et al. 2008 [5] | ALL | pregnancy | Any | 1.2 (0.9, 1.5) | 153/494 | 325/1356 | 2003-2004 | France | <15 y | Interview |
|  | 49.2 |  |  |  | 1-10 | 1.3 (38718) | 111/494 | 220/1356 |  |  |  |  |
|  | 49.3 |  |  |  | >10 | 1.0 (0.6, 1.4) | 38/494 | 99/1356 |  |  |  |  |
|  | 49.4 |  | AML |  | Any | 1.3 (0.8, 2.1) | 25/77 | 325/1356 |  |  |  |  |
|  | 49.5 |  |  |  | 1-10 | 1.5 (0.9, 2.5) | 19/77 | 220/1356 |  |  |  |  |
|  | 49.6 |  |  |  | >10 | 0.9 (0.4, 2.2) | 6/77 | 99/1356 |  |  |  |  |
|  | 49.7 |  | Acute Leukemia |  | Any | 0.65 (0.542, 0.79) | 178/571 | 650/1356 |  |  |  |  |
|  | 49.8 |  |  |  | 1-10 | 0.70 (0.56, 0.83) | 130/571 | 440/1356 |  |  |  |  |
|  | 49.9 |  |  |  | >10 | 0.53 (0.38, 0.74) | 44/571 | 198/1356 |  |  |  |  |
|  | 49.10 |  | Hodgkin Lymphoma |  | Any | 0.9 (0.6, 1.4) | 27/101 | 172/676 |  |  | 5-14 y |  |
|  | 49.11 |  |  |  | 1-10 | 0.9 (0.5, 1.6) | 16/101 | 112/676 |  |  |  |  |
|  | 49.12 |  |  |  | >10 | 0.8 (0.4, 1.8) | 10/101 | 56/676 |  |  |  |  |
|  | 49.13 |  | Non-Hodgkin Lymphoma |  | Any | 1.5 (1.0, 1.8) | 45/119 | 265/1047 |  |  | 1-15 y |  |
|  | 49.14 |  |  |  | 1-10 | 1.6 (1.1, 2.5) | 33/119 | 177/1047 |  |  |  |  |
|  | 49.15 |  |  |  | >10 | 1.0 (0.5, 1.9) | 10/119 | 82/1047 |  |  |  |  |
|  | 49.16 |  | Lymphoma |  | Any | 1.29 (0.7, 1.72) | 72/220 | 437/1723 |  |  |  |  |
|  | 49.17 |  |  |  | 1-10 | 1.33 (0.95, 1.8) | 49/220 | 289/1723 |  |  |  |  |
|  | 49.18 |  |  |  | >10 | 1.14 (0.69, 1.85) | 20/220 | 138/1723 |  |  |  |  |
| 50 | 50.1 | Schraw et al. 2014 [57] | ALL | pregnancy | Any | 2.82 (1.59, 6.86) | 12/130 | 9/275 | 1997-2001 | US | 0-14 y | Interview |
| 51 | 51.1 | Schüz et al. 1999 [6] | Acute Leukemia | pregnancy | 1-10 | 0.8 (0.6, 1.1) | 159/792 | 174/765 | 1992-1994 | Germany | < 15 y | Question-naire |
|  | 51.2 |  |  |  | 11-20 | 0.5 (0.3, 0.9) | 25/792 | 39/765 |  |  |  |  |
|  | 51.3 |  |  |  | ≥ 20 | 1.3 (0.4, 4.7) | 6/792 | 4/765 |  |  |  |  |
|  | 51.4 |  |  |  | Any | 0.85 (0.68, 1.05) | 190/792 | 217/765 |  |  |  |  |
|  | 51.5 |  | Non-Hodgkin Lymphoma |  | 1-10 | 1.3 (0.9, 1.9) | 46/173 | 426/2062 |  |  | 15 y |  |
|  | 51.6 |  |  |  | 11-20 | 1.0 (0.4, 2.5) | 6/173 | 72/2062 |  |  |  |  |
|  | 51.7 |  |  |  | ≥ 20 | 5.2 (1.2, 22.4) | 3/173 | 11/2062 |  |  |  |  |
|  | 51.8 |  |  |  | Any | 1.29 (0.94, 1.77) | 55/173 | 509/2062 |  |  |  |  |
|  | 51.9 |  | Nervous system tumor |  | 1-10 | 0.8 (0.6, 1.1) | 55/320 | 426/2062 | 1980-1994 |  |  |  |
|  | 51.10 |  |  |  | 11-20 | 1.6 (0.9, 2.8) | 17/320 | 72/2062 |  |  |  |  |
|  | 51.11 |  |  |  | ≥ 20 | 0.8 (0.2, 3.9) | 2/320 | 11/2062 |  |  |  |  |
|  | 51.12 |  |  |  | Any | 0.94 (0.71, 1.23) | 74/320 | 509/2062 | 1992-1994 |  |  |  |
|  | 51.13 |  | Neuroblastoma |  | 1-10 | 1.5 (37288) | 39/114 | 426/2062 | 1980-1994 |  |  |  |
|  | 51.14 |  |  |  | 11-20 | 0.6 (0.2, 2) | 3/114 | 72/2062 |  |  |  |  |
|  | 51.15 |  |  |  | ≥ 20 | 2.5 (0.6, 10.4) | 3/114 | 11/2062 |  |  |  |  |
|  | 51.16 |  |  |  | Any | 1.59 (1.12, 2.29) | 45/114 | 509/2062 | 1992-1994 |  |  |  |
|  | 51.17 |  | Nephroblastoma |  | 1-10 | 0.9 (0.5, 1.4) | 22/117 | 426/2062 | 1980-1994 |  |  |  |
|  | 51.18 |  |  |  | 11-20 | 1.2 (0.5, 3) | 6/117 | 72/2062 |  |  |  |  |
|  | 51.19 |  |  |  | Any | 0.99 (0.65, 1.51) | 28/117 | 498/2062 | 1992-1994 |  |  |  |
|  | 51.20 |  | Bone cancer |  | 1-10 | 0.7 (0.3, 1.4) | 10/82 | 426/2062 | 1980-1994 |  |  |  |
|  | 51.21 |  |  |  | 11-20 | 0.9 (0.2, 3.9) | 2/82 | 72/2062 |  |  |  |  |
|  | 51.22 |  |  |  | ≥ 20 | 2.5 (0.3, 22.4) | 1/82 | 11/2062 |  |  |  |  |
|  | 51.23 |  |  |  | Any | 0.64 (0.35, 1.16) | 13/82 | 509/2062 | 1992-1994 |  |  |  |
|  | 51.24 |  | Soft tissue sarcoma |  | 1-10 | 0.9 (0.5, 1.4) | 20/113 | 426/2062 | 1980-1994 |  |  |  |
|  | 51.25 |  |  |  | 11-20 | 0.8 (0.2, 2.6) | 3/113 | 72/2062 |  |  |  |  |
|  | 51.26 |  |  |  | ≥ 20 | 1.6 (0.2, 13.3) | 1/113 | 11/2062 |  |  |  |  |
|  | 51.27 |  |  |  | Any | 0.86 (0.55, 1.35) | 24/113 | 509/2062 | 1992-1994 |  |  |  |
| 52 | 52.1 | Schüz et al. 2001a [25] | Nervous system tumor | pregnancy | 1-10 | 0.77 (0.57, 1.04) | 66/376 | 416/1944 | 1993-1997 | Germany | <15 y | Question-naire |
|  | 52.2 |  |  |  | 11-20 | 1.45 (0.83, 2.55) | 17/376 | 66/1944 |  |  |  |  |
|  | 52.3 |  |  |  | >21 | 0.82 (0.18, 3.76) | 2/376 | 12/1944 |  |  |  |  |
|  | 52.4 |  |  |  | Any | 0.89 (0.69, 1.15) | 85/376 | 494/1944 |  |  |  |  |
|  | 52.5 |  | Astrocytoma |  | 1-10 | 0.87 (0.51, 1.5) | 18/97 | 416/1944 |  |  |  |  |
|  | 52.6 |  |  |  | 11-20 | 0.96 (0.28, 3.29) | 3/97 | 66/1944 |  |  |  |  |
|  | 52.7 |  |  |  | Any | 0.87 (0.54, 1.41) | 21/97 | 482/1944 |  |  |  |  |
|  | 52.8 |  | Ependymoma |  | 1-10 | 0.94 (0.4, 2.18) | 7/36 | 416/1944 |  |  |  |  |
|  | 52.9 |  |  |  | 11-20 | 5.66 (2.01, 15.9) | 6/36 | 66/1944 |  |  |  |  |
|  | 52.10 |  |  |  | Any | 1.46 (0.767, 2.77) | 13/36 | 482/1944 |  |  |  |  |
|  | 52.11 |  | Glioma |  | 1-10 | 0.88 (0.57, 1.36) | 25/133 | 416/1944 |  |  |  |  |
|  | 52.12 |  |  |  | 11-20 | 1.99 (0.97, 4.09) | 9/133 | 66/1944 |  |  |  |  |
|  | 52.13 |  |  |  | Any | 1.03 (0.69, 1.52) | 34/133 | 482/1944 |  |  |  |  |
|  | 52.14 |  | Medulloblastoma |  | 1-10 | 0.9 (0.52, 1.53) | 21/87 | 416/1944 |  |  |  |  |
|  | 52.15 |  |  |  | 11-20 | 0.34 (0.05, 2.54) | 1/87 | 66/1944 |  |  |  |  |
|  | 52.16 |  |  |  | >21 | 4.7 (0.84, 26.3) | 2/87 | 12/1944 |  |  |  |  |
|  | 52.17 |  |  |  | Any | 1.09 (0.68, 1.72) | 24/87 | 494/1944 |  |  |  |  |
| 53 | 53.1 | Schüz et al. 2001b [33] | Wilms’ tumor | pregnancy | 1-10 | 0.86 (0.55, 1.36) | 26/138 | 347/1574 | 1993-1997 | Germany | <15 y | Question-naire |
|  | 53.2 |  |  |  | 11+ | 1.19 (0.54, 2.62) | 9/138 | 69/1574 |  |  |  |  |
|  | 53.3 |  |  |  | Any | 0.96 (0.65, 1.41) | 35/138 | 416/1574 |  |  |  |  |
| 54 | 54.1 | Schüz et al. 2001c [26] | Neuroblastoma | pregnancy | 1-10 | 1.39 (0.93, 2.09) | 41/133 | 312/1396 | 1988-1994 | Germany | <8 y | Question-naire |
|  | 54.2 |  |  |  | 11-20 | 0.96 (0.35, 2.61) | 5/133 | 53/1396 |  |  |  |  |
|  | 54.3 |  |  |  | >20 | 2.57 (0.61, 10.8) | 3/133 | 9/1396 |  |  |  |  |
|  | 54.4 |  |  |  | Any | 1.38 (0.97, 1.95) | 49/133 | 374/1396 |  |  |  |  |
| 55 | 55.1 | Severson et al. 1993 [58] | AML | pregnancy | Any | 1.2 (0.77, 1.86) |  |  | 1980-1984 | US & Canada | 0-17 y | Interview |
| 56 | 56.1 | Shu et al. 1995 [39] | Germ cell tumor | 3 months before pregnancy | Any | 0.6 (0.3, 1) | 23/82 | 215/424 | 1982-1989 | US & Canada | 0-14 y | Question-naire |
|  | 56.2 |  |  | pregnancy | 1-15 | 0.7 (0.1, 1.5) | 12/88 | 86/481 |  |  |  |  |
|  | 56.3 |  |  |  | >15 | 0.3 (0.1, 0.8) | 5/88 | 72/481 |  |  |  |  |
|  | 56.4 |  |  |  | Any | 0.59 (0.34, 1.02) | 17/88 | 158/481 |  |  |  |  |
| 57 | 57.1 | Shu et al. 1996 [59] | Leukemia | 1 month bef pregnancy | Any | 0.71 (0.51, 1.01) | /302 | not reported | 1983-1988 | Multi | 0-1,5 y | Interview |
|  | 57.2 |  |  | pregnancy |  | 0.66 (0.46, 0.94) | /302 |  |  |  |  |  |
|  | 57.3 |  |  | 1. trimester |  | 0.62 (0.43, 0.9) | /302 |  |  |  |  |  |
|  | 57.4 |  |  | 2. trimester |  | 0.66 (0.45, 0.98) | /302 |  |  |  |  |  |
|  | 57.5 |  |  | 3. trimester |  | 0.69 (0.47, 1.02) | /302 |  |  |  |  |  |
|  | 57.6 |  |  | Breast feeding |  | 0.49 (0.29, 0.83) | /302 |  |  |  |  |  |
|  | 57.7 |  |  | pregnancy | 1-10 | 0.66 (0.41, 1.04) | /302 |  |  |  |  |  |
|  | 57.8 |  |  |  | 11-20 | 0.64 (0.39, 1.06) | /302 |  |  |  |  |  |
|  | 57.9 |  |  |  | >20 | 0.62 (0.22, 1.79) | /302 |  |  |  |  |  |
|  | 57.10 |  | ALL | 1 month bef pregnancy | Any | 0.84 (0.51, 1.28) | /203 |  |  |  |  |  |
|  | 57.11 |  |  | pregnancy |  | 0.78 (0.51, 1.18) | /203 |  |  |  |  |  |
|  | 57.12 |  |  | 1. trimester |  | 0.72 (0.47, 1.11) | /203 |  |  |  |  |  |
|  | 57.13 |  |  | 2. trimester |  | 0.73 (0.46, 1.17) | /203 |  |  |  |  |  |
|  | 57.14 |  |  | 3. trimester |  | 0.81 (0.51, 1.28) | /203 |  |  |  |  |  |
|  | 57.15 |  |  | breast feeding |  | 0.49 (0.26, 0.93) | /203 |  |  |  |  |  |
|  | 57.16 |  |  | pregnancy | 1-10 | 0.78 (0.45, 1.32) | /203 |  |  |  |  |  |
|  | 57.17 |  |  |  | 11-20 | 0.79 (0.44, 1.42) | /203 |  |  |  |  |  |
|  | 57.18 |  |  |  | >20 | 0.48 (0.12, 1.9) | /203 |  |  |  |  |  |
|  | 57.19 |  | AML | 1 month bef pregnancy | Any | 0.48 (0.22, 1.05) | /88 |  |  |  |  |  |
|  | 57.20 |  |  | pregnancy |  | 0.45 (0.21, 0.96) | /88 |  |  |  |  |  |
|  | 57.21 |  |  | 1. trimester |  | 0.45 (0.21, 0.96) | /88 |  |  |  |  |  |
|  | 57.22 |  |  | 2. trimester |  | 0.6 (0.28, 1.3) | /88 |  |  |  |  |  |
|  | 57.23 |  |  | 3. trimester |  | 0.59 (0.27, 1.25) | /88 |  |  |  |  |  |
|  | 57.24 |  |  | breast feeding |  | 0.61 (0.23, 1.57) | /88 |  |  |  |  |  |
|  | 57.25 |  |  | pregnancy | 1-10 | 0.46 (0.16, 1.31) | /88 |  |  |  |  |  |
|  | 57.26 |  |  |  | 11-20 | 0.41 (0.15, 1.13) | /88 |  |  |  |  |  |
|  | 57.27 |  |  |  | >20 | 0.69 (0.08, 5.78) | /88 |  |  |  |  |  |
| 58 | 58.1 | Shu et al. 1999 [60] | AML | pregnancy | Any | 1.05 (0.78, 1.41) | 130/326 | 147/392 | 1989-1993 | Multi | 14 y | Interview |
|  | 58.2 |  | ALL |  |  | 1.09 (0.96, 1.27) | 512/1232 | 522/1357 |  |  |  |  |
|  | 58.3 |  | Acute Leukemia |  |  | 1.08 (0.95, 1.22) | 642/1558 | 669/1749 |  |  |  |  |
| 59 | 59.1 | Slater et al. 2011 [61] | Acute Leukemia | pregnancy | Any | 0.8 (0.52, 1.24) | 74/369 | 65/259 | 1996-2006 | US & Canada | < 1 y | Interview |
|  | 59.2 |  | ALL |  |  | 0.87 (0.54, 1.4) | 50/214 | 65/259 |  |  |  |  |
|  | 59.3 |  | AML |  |  | 0.74 (0.4, 1.35) | 23/149 | 65/259 |  |  |  |  |
| 60 | 60.1 | Tuomisto et al. 2009 [40] | Testicular cancer | pregnancy | Any | 0.78 (0.41, 1.49) | 16/54 | 124/395 | 1976-2006 | Multi | <9 y | Serum sample |
| 61 | 61.1 | van Dujin et al. 1994 [62] | Non-lymphocytic leukemia | pregnancy | Any | 0.6 (0.3, 1.2) | 22/58 | 75/165 | 1973-1980 | the Netherlands | <14 y | Question-naire |
| 62 | 62.1 | Yang et al. 2000 [27] | Neuroblastoma | around pregnancy (1 month bef, pregnancy to breast feeding) | Any | 1.1 (0.8, 1.4) | 150/351 | 132/371 | 1992-1994 | US | <19 y | Interview |
|  | 62.2 |  |  | 1 month bef pregnancy |  | 1.1 (0.8, 1.4) | 147/354 | 132/371 |  |  |  |  |
|  | 62.3 |  |  | 1. trimester |  | 1.0 (0.7, 1.3) | 111/390 | 102/401 |  |  |  |  |
|  | 62.4 |  |  | 2. trimester |  | 0.9 (0.7, 1.3) | 87/414 | 82/421 |  |  |  |  |
|  | 62.5 |  |  | 3. trimester |  | 1.0 (0.7, 1.4) | 91/410 | 82/421 |  |  |  |  |
|  | 62.6 |  |  | breast feeding |  | 0.9 (0.5, 1.5) | 31/364 | 38/377 |  |  |  |  |
|  | 62.7 |  |  | 1. trimester | ≤5 | 0.8 (0.5, 1.5) | 22/390 | 25/401 |  |  |  |  |
|  | 62.8 |  |  |  | 5-10 | 1.0 (0.6, 1.6) | 39/390 | 34/401 |  |  |  |  |
|  | 62.9 |  |  |  | 1-10 | 1.06 (0.72, 1.56) | 61/390 | 59/401 |  |  |  |  |
|  | 62.10 |  |  |  | 10-15 | 1.2 (0.5, 3.0) | 12/390 | 9/401 |  |  |  |  |
|  | 62.11 |  |  |  | >15 | 0.9 (0.6, 1.6) | 37/390 | 33/401 |  |  |  |  |
|  | 62.12 |  |  | 2. trimester | ≤5 | 1.0 (0.5, 2.0) | 17/414 | 16/421 |  |  |  |  |
|  | 62.13 |  |  |  | 5-10 | 1.0 (0.6, 1.6) | 34/414 | 30/421 |  |  |  |  |
|  | 62.14 |  |  |  | 1-10 | 1.13 (0.74, 1.72) | 51/414 | 46/421 |  |  |  |  |
|  | 62.15 |  |  |  | 10-15 | 1.3 (0.5, 3.5) | 11/414 | 7/421 |  |  |  |  |
|  | 62.16 |  |  |  | >15 | 0.8 (0.4, 1.4) | 24/414 | 27/421 |  |  |  |  |

ALL: acute lymphoblastic leukemia; AML: acute myeloblastic leukemia; PNET: primitive neuroectodermal tumor, HL: Hodgkin lymphoma; NHL: non-Hodgkin lymphoma; CPD: cigarettes per day; OR: odds ratio; ^: Hazard ratio; * Risk ratio; CI: confidence interval; ^†^: cohort study

# S1.c. Results subgroup analyses

Meta-analyses were conducted for eight cancer types and their association with maternal smoking. In addition to the main results presented in the main article (see main Article, Figure 4) the results of all subgroup analyses conducted in the context of this meta-analysis are presented here (Tables S4-S11.b). Due to the lack of available original articles, not all planned subgroup analyses for each cancer type (see Table S3) could be conducted. “Included datasets” refer to the second column in the review table (Table S2). Please note, that for cohort studies the reported risk estimate was not OR but HR. Please refer to Table S2 for more details.

Cochran Q statistics (p value < 0.1 as level for significance) [88] and Higgins et al. [89] I^2^ statistics were used for assessment of heterogeneity. Thresholds for the interpretation of I^2^ were set according to the Cochrane Collaboration (2011) (I^2^<40% no heterogeneity; I^2^=40-59% moderate; I^2^=50-79% considerable; I^2^≥80% substantial heterogeneity) [88].

#### Table D. Subgroup analyses of risk for lymphoma in early life due to maternal smoking

|  | Number of studies | Included cases | Fixed effect model  (95%CI) | | Random effect model  (95%CI) | | Heterogeneity | | | | Included datasets |
| --- | --- | --- | --- | --- | --- | --- | --- | --- | --- | --- | --- |
|  |  |  |  |  |  |  | Q (p-value) | Inter- pretation | I^2^ | Inter- pretation |  |
| Time of exposure | |  |  |  |  |  |  |  |  |  |  |
| During pregnancy | 6 | 1047 | 1.21 (1.05, 1.39) | | 1.21 (1.01, 1.45) | | 0.9 | no | <0 | no | 49.16; 37.10; 51.8; 43.1; 43.2; 29.6 |
| Amount of smoking |  |  |  |  |  |  |  |  |  |  |  |
| <11 CPD | 3 | 559 | 1.28 (1.03, 1.60) | | 1.28 (0.96, 1.69) | | 0.9 | no | <0 | no | 49.17; 37.11; 51.5 |
| >10 CPD | 2 | 306 | 1.19 (0.80, 1.78) | | 1.19 (0.80, 1.78) | | 0.7 | no | <0 | no | 49.18 37.12 |
| Risk estimate |  |  |  |  |  |  |  |  |  |  |  |
| HR | 3 | 508 | 1.13 (0.92, 1.38) | | 1.13 (0.92, 1.38) | | 0.8 | no | <0 | no | 37.10; 43.1; 43.2 |
| OR | 3 | 539 | 1.30 (1.06, 1.60) | | 1.32 (0.95, 1.85) | | 0.9 | no | <0 | no | 49.16; 51.8; 29.6 |
| Study design |  |  |  |  |  |  |  |  |  |  |  |
| case-control | 3 | 539 | 1.30 (1.06, 1.60) | | 1.32 (0.95, 1.85) | | 0.9 | no | <0 | no | 49.16; 51.8; 29.6 |
| cohort | 3 | 508 | 1.13 (0.92, 1.38) | | 1.13 (0.92, 1.38) | | 0.8 | no | <0 | no | 37.10; 43.1; 43.2 |
| Number of exposed cases | |  |  |  |  |  |  |  |  |  |  |
| <50 | 3 | 187 | 1.15 (0.84, 1.58) | | 1.15 (0.84, 1.58) | | 0.6 | no | <0 | no | 37.10; 43.2; 29.6 |
| <100 | 3 | 860 | 1.22 (1.04, 1.44) | | 1.23 (0.96, 1.58) | | 0.8 | no | <0 | no | 49.16; 51.8; 43.1 |
| Decade of cancer diagnosis | | |  |  |  |  |  |  |  |  |  |
| 90-99 | 4 | 736 | 1.17 (0.99, 1.38) | | 1.17 (0.99, 1.38) | | 0.8 | no | <0 | no | 37.10; 51.8; 43.1; 43.2 |
| Study region |  |  |  |  |  |  |  |  |  |  |  |
| Europe | 6 | 1047 | 1.21 (1.05, 1.39) | | 1.21 (1.01, 1.45) | | 0.9 | no | <0 | no | 49.16; 37.10; 51.8; 43.1; 43.2; 29.6 |
| Study country |  |  |  |  |  |  |  |  |  |  |  |
| Sweden | 3 | 508 | 1.13 (0.92, 1.38) | | 1.13 (0.92, 1.38) | | 0.8 | no | <0 | no | 37.10; 43.1; 43.2 |
| Age at cancer diagnosis | | |  |  |  |  |  |  |  |  |  |
| <15 | 4 | 738 | 1.19 (1.00, 1.41) | | 1.19 (0.96, 1.46) | | 0.7 | no | <0 | no | 49.16; 43.1; 43.2; 29.6 |
| Exposure assessment | | |  |  |  |  |  |  |  |  |  |
| birth register | 3 | 508 | 1.13 (0.92, 1.38) | | 1.13 (0.92, 1.38) | | 0.8 | no | <0 | no | 37.10; 43.1; 43.2 |
| interview | 2 | 311 | 1.32 (1.00, 1.73) | | 1.36 (0.85, 2.17) | | 0.6 | no | <0 | no | 49.16; 29.6 |
| Adjustment of risk estimate | | | |  |  |  |  |  |  |  |  |
| yes | 5 | 819 | 1.19 (1.01, 1.40) | | 1.19 (0.99, 1.43) | | 0.8 | no | <0 | no | 49.16; 37.10; 43.1; 43.2; 29.6 |

#### Table D.a. Subgroup analyses of risk for non-Hodgkin’s lymphoma in early life due to maternal smoking

|  | Number of studies | | Included cases | | Fixed effect model  (95%CI) | | | Random effect model  (95%CI) | | Heterogeneity | | | | | Included datasets |
| --- | --- | --- | --- | --- | --- | --- | --- | --- | --- | --- | --- | --- | --- | --- | --- |
|  |  |  |  |  |  |  |  |  |  | Q (p-value) | | Inter- pretation | I^2^ | Inter- pretation |  |
| Time of exposure | |  |  |  | |  |  | |  | |  |  |  |  |  |
| During pregnancy | | 5 | 832 | 1.26 (1.07, 1.48) | | | 1.29 (1.01, 1.66) | | | | 0.8 | no | <0 | no | 49.13; 37.10; 51.8; 43.1; 29.6 |
| Amount of smoking | |  |  |  | |  |  | |  | |  |  |  |  |  |
| <11 CPD | | 3 | 442 | 1.36 (1.07, 1.73) | | | 1.35 (0.92, 1.97) | | | | 0.6 | no | <0 | no | 49.14; 37.11; 51.5 |
| >10 CPD | | 2 | 195 | 1.15 (0.71, 1.86) | | | 1.15 (0.71, 1.86) | | | | 0.6 | no | <0 | no | 49.15; 37.12 |
| Risk estimate | |  |  |  | |  |  | |  | |  |  |  |  |  |
| HR | | 2 | 421 | 1.16 (0.93, 1.44) | | | 1.16 (0.93, 1.44) | | | | 0.8 | no | <0 | no | 49.15; 37.12 |
| OR | | 3 | 411 | 1.39 (1.10, 1.75) | | | 1.42 (0.93, 2.18) | | | | 0.8 | no | <0 | no | 49.13; 51.8; 29.6 |
| Study design | |  |  |  | |  |  | |  | |  |  |  |  |  |
| case-control | | 3 | 411 | 1.39 (1.10, 1.75) | | | 1.42 (0.93, 2.18) | | | | 0.8 | no | <0 | no | 49.13; 51.8; 29.6 |
| cohort | | 2 | 421 | 1.16 (0.93, 1.44) | | | 1.16 (0.93, 1.44) | | | | 0.8 | no | <0 | no | 49.15; 37.12 |
| Number of exposed cases | | | |  | |  |  | |  | |  |  |  |  |  |
| <50 | | 3 | 264 | 1.41 (1.07, 1.86) | | | 1.41 (0.93, 2.13) | | | | 0.7 | no | <0 | no | 49.13; 37.10; 29.6 |
| <100 | | 2 | 568 | 1.19 (0.98, 1.45) | | | 1.20 (0.91, 1.60) | | | | 0.6 | no | <0 | no | 51.8; 43.1 |
| Decade of cancer diagnosis | | | |  | |  |  | |  | |  |  |  |  |  |
| 90-99 | | 3 | 649 | 1.20 (1.00, 1.43) | | | 1.20 (0.96, 1.51) | | | | 0.9 | no | <0 | no | 51.8; 43.1 |
| Study region | |  |  |  | |  |  | |  | |  |  |  |  |  |
| Europe | | 5 | 832 | 1.26 (1.07, 1.48) | | | 1.29 (1.01, 1.66) | | | | 0.8 | no | <0 | no | 49.13; 37.10; 51.8; 43.1; 29.6 |
| Study country | |  |  |  | |  |  | |  | |  |  |  |  |  |
| Sweden | | 2 | 421 | 1.16 (0.93, 1.44) | | | 1.16 (0.93, 1.44) | | | | 0.8 | no | <0 | no | 49.15; 37.12 |
| Age at cancer diagnosis | | | |  | |  |  | |  | |  |  |  |  |  |
| <15 | | 3 | 523 | 1.26 (1.03, 1.54) | | | 1.34 (0.91, 1.96) | | | | 0.4 | no | <0 | no | 49.13; 43.1; 29.6 |
| Exposure assessment | |  |  |  | |  |  | |  | |  |  |  |  |  |
| birth register | | 2 | 421 | 1.16 (0.93, 1.44) | | | 1.16 (0.93, 1.44) | | | | 0.8 | no | <0 | no | 49.15; 37.12 |
| interview | | 2 | 183 | 1.52 (1.08, 2.14) | | | 1.55 (0.86, 2.80) | | | | 0.8 | no | <0 | no | 49.13; 29.6 |
| Adjustment of risk estimate | | | |  | |  |  | |  | |  |  |  |  |  |
| yes | | 4 | 604 | 1.25 (1.04, 1.51) | | | 1.30 (0.97, 1.74) | | | | 0.6 | no | <0 | no | 49.13; 37.10; 43.1; 29.6 |

#### Table E. Subgroup analyses of risk for nervous system tumors in early life due to maternal smoking

|  | Number of studies | | Included cases | Fixed effect model  (95%CI) | | Random effect model  (95%CI) | | Heterogeneity | | | | | Included datasets |
| --- | --- | --- | --- | --- | --- | --- | --- | --- | --- | --- | --- | --- | --- |
|  |  |  |  |  |  |  |  | Q (p-value) | | Inter- pretation | I^2^ | Inter- pretation |  |
| Time of exposure | | |  |  |  |  |  | |  |  |  |  |  |
| During pregnancy | 22 | | 6078 | 1.09 (1.02, 1.17) | | 1.11 (1.01, 1.22) | | 0.1 | | Yes | 0.3 | no | 36.1; 16.1; 52.4; 7.3; 10.1; 14.1; 21.1; 38.6; 51.12; 51.16; 45.1; 15.4; 5.1; 62.1; 54.4; 22.1; 32.1; 17.9; 4.1; 47.1; 26.1; 34.4 |
| 1st trimester | 3 | | 1073 | 1.12 (0.93, 1.34) | | 1.13 (0.87, 1.46) | | 0.1 | | yes | 0.5 | moderate | 38.1; 15.1; 62.3 |
| 2nd trimester | 2 | | 814 | 0.90 (0.72, 1.14) | | 0.90 (0.72, 1.14) | | 0.9 | | no | <0 | no | 38.2; 62.4 |
| 3rd trimester | 2 | | 815 | 0.98 (0.78, 1.24) | | 0.98 (0.78, 1.24) | | 0.9 | | no | <0 | no | 38.3; 62.5 |
| Amount of smoking | | | |  |  |  |  | |  |  |  |  |  |
| <11 CPD | | 14 | 3513 | 1.03 (0.94, 1.14) | | 1.06 (0.92, 1.21) | | 0.02 | | yes | 0.4 | moderate | 36.2; 52.1; 14.2; 38.4; 51.9; 51.13; 45.2; 15.5; 5.2; 54.1; 17.7; 4.2; 34.1; 28.10 |
| 11-20 CPD | | 5 | 1378 | 1.35 (1.01, 1.81) | | 1.32 (0.93, 1.87) | | 0.6 | | no | <0 | no | 52.2; 51.10; 51.14; 54.2; 34.2 |
| >10 CPD | | 8 | 1525 | 1.07 (0.90, 1.27) | | 1.07 (0.90, 1.27) | | 0.7 | | no | <0 | no | 14.3; 38.5; 45.3; 15.6; 5.3; 17.8; 4.3; 28.11 |
| >20 CPD | | 6 | 1590 | 1.01 (0.71, 1.44) | | 1.01 (0.71, 1.44) | | 0.5 | | no | <0 | no | 36.3; 52.3; 51.11; 51.15; 54.3; 34.3 |
| Risk estimate | |  |  |  |  |  |  | |  |  |  |  |  |
| OR | | 20 | 5524 | 1.07 (0.99, 1.15) | | 1.10 (0.99, 1.21) | | 0.1 | | yes | 0.3 | no | 36.1; 16.1; 52.4; 7.3; 10.1; 14.1; 38.6; 51.12; 51.16; 45.1; 15.4; 5.1; 62.1; 54.4; 22.1; 32.1; 17.9; 47.1; 26.1; 34.4 |
| Study design | |  |  |  |  |  |  | |  |  |  |  |  |
| case-control | | 21 | 5598 | 1.07 (0.99, 1.15) | | 1.10 (1.00, 1.21) | | 0.1 | | yes | 0.3 | no | 36.1; 16.1; 52.4; 7.3; 10.1; 14.1; 21.1; 38.6; 51.12; 51.16; 45.1; 15.4; 5.1; 62.1; 54.4; 22.1; 32.1; 17.9; 47.1; 26.1; 34.4 |
| Number of exposed cases | | | |  |  |  |  | |  |  |  |  |  |
| <50 | | 9 | 1119 | 1.34 (1.14, 1.57) | | 1.34 (1.06, 1.68) | | 0.8 | | no | <0 | no | 10.1; 14.1; 21.1; 51.16; 45.1; 15.4; 54.4; 22.1; 32.1 |
| <100 | | 7 | 2119 | 1.04 (0.93, 1.17) | | 1.05 (0.90, 1.22) | | 0.1 | | yes | 0.4 | moderate | 36.1; 52.4; 38.6; 51.12; 5.1; 47.1; 34.4 |
| <150 | | 3 | 1123 | 1.10 (0.95, 1.27) | | 1.09 (0.93, 1.28) | | 0.2 | | no | 0.3 | no | 7.3; 17.9; 4.1 |
| Decade of cancer diagnosis | | | |  |  |  |  | |  |  |  |  |  |
| 70-79 | | 3 | 531 | 1.01 (0.82, 1.23) | | 1.01 (0.82, 1.23) | | 0.6 | | no | <0 | no | 17.9; 47.1; 26.1 |
| 80-89 | | 9 | 2608 | 1.09 (0.97, 1.22) | | 1.15 (0.97, 1.37) | | 0.1 | | yes | 0.4 | moderate | 16.1; 7.3; 10.1; 14.1; 21.1; 38.6; 5.1; 32.1; 34.4 |
| 90-99 | | 8 | 2430 | 1.14 (1.03, 1.27) | | 1.16 (0.98, 1.38) | | 0.1 | | yes | 0.4 | no | 52.4; 51.12; 51.16; 15.4; 62.1; 54.4; 22.1; 4.1 |
| 00-09 | | 2 | 509 | 0.97 (0.77, 1.22) | | 0.97 (0.77, 1.22) | | 0.4 | | no | <0 | no | 36.1; 45.1 |
| Study region | |  |  |  |  |  |  | |  |  |  |  |  |
| Europe | | 12 | 3625 | 1.12 (1.03, 1.22) | | 1.17 (1.01, 1.37) | | 0.01 | | yes | 0.5 | moderate | 16.1; 52.4; 10.1; 14.1; 51.12; 51.16; 45.1; 15.4; 54.4; 32.1; 4.1; 34.4 |
| North America | | 8 | 1871 | 1.06 (0.94, 1.20) | | 1.06 (0.94, 1.20) | | 0.8 | | no | <0 | no | 7.3; 21.1; 38.6; 5.1; 62.1; 17.9; 47.1; 26.1 |
| Study country | |  |  |  |  |  |  | |  |  |  |  |  |
| France | | 2 | 284 | 1.18 (0.86, 1.62) | | 1.23 (0.80, 1.89) | | 0.3 | | no | <0 | no | 10.1; 45.1 |
| Germany | | 5 | 1507 | 1.16 (1.02, 1.32) | | 1.20 (0.92, 1.56) | | 0.01 | | yes | 0.7 | moderate | 52.4; 51.12; 51.16; 54.4; 34.4 |
| Italy | | 2 | 262 | 1.47 (1.02, 2.12) | | 1.48 (0.84, 2.61) | | 0.7 | | no | <0 | no | 14.1; 15.4 |
| US | | 6 | 1476 | 1.05 (0.92, 1.20) | | 1.05 (0.92, 1.20) | | 0.7 | | no | <0 | no | 38.6; 5.1; 62.1; 17.9; 47.1; 26.1 |
| Multi | | 2 | 1537 | 0.93 (0.79, 1.09) | | 0.93 (0.79, 1.09) | | 0.4 | | no | <0 | no | 16.1; 7.3 |
| Age at cancer diagnosis | | | |  |  |  |  | |  |  |  |  |  |
| <6 | | 2 | 432 | 1.14 (0.88, 1.48) | | 1.16 (0.84, 1.60) | | 0.3 | | no | 0.0 | no | 7.3; 5.1 |
| <15 | | 5 | 1357 | 1.05 (0.92, 1.21) | | 1.05 (0.86, 1.28) | | 0.1 | | yes | 0.5 | moderate | 36.1; 52.4; 45.1; 32.1; 34.4 |
| >14 | | 12 | 3627 | 1.04 (0.95, 1.15) | | 1.08 (0.95, 1.22) | | 0.2 | | no | 0.2 | no | 16.1; 10.1; 14.1; 21.1; 38.6; 51.12; 51.16; 15.4; 62.1; 22.1; 17.9; 47.1 |
| Exposure assessment | | | |  |  |  |  | |  |  |  |  |  |
| interview | | 14 | 3700 | 1.03 (0.94, 1.13) | | 1.03 (0.94, 1.13) | | 0.7 | | no | <0 | no | 16.1; 7.3; 10.1; 21.1; 38.6; 45.1; 15.4; 5.1; 62.1; 22.1; 32.1; 17.9; 47.1; 26.1 |
| questionnaire | | 7 | 1989 | 1.13 (1.00, 1.27) | | 1.17 (0.94, 1.46) | | 0.01 | | yes | 0.6 | moderate | 36.1; 52.4; 14.1; 51.12 |
| Adjustment of risk estimate | | | |  |  |  |  | |  |  |  |  |  |
| yes | | 14 | 4251 | 1.06 (0.97, 1.15) | | 1.07 (0.97, 1.19) | | 0.2 | | no | 0.2 | no | 36.1; 16.1; 52.4; 10.1 |
| no | | 8 | 1827 | 1.14 (1.02, 1.29) | | 1.17 (0.97, 1.40) | | 0.1 | | yes | 0.4 | moderate | 7.3; 51.12; 51.16; 5.1; 17.9; 47.1; 26.1; 34.4 |

#### Table E.a. Subgroup analyses of risk for neuroblastoma in early life due to maternal smoking

|  | Number of studies | Included cases | | Fixed effect model  (95%CI) | | Random effect model  (95%CI) | Heterogeneity | | | | | Included datasets |
| --- | --- | --- | --- | --- | --- | --- | --- | --- | --- | --- | --- | --- |
|  |  |  |  |  |  |  | Q (p-value) | | Inter- pretation | I^2^ | Inter- pretation |  |
| Time of exposure | |  |  | |  |  |  |  |  |  |  |  |
| During pregnancy | 5 | 953 | 1.30 (1.10, 1.53) | | | 1.32 (1.01, 1.74) | 0.6 | | no | <0 | no | 51.16; 5.1; 62.1; 54.4; 26.1 |
| Amount of smoking |  |  |  | |  |  |  |  |  |  |  |  |
| <11 CPD | 3 | 479 | 1.40 (1.11, 1.75) | | | 1.39 (0.95-2.05) | 0.9 | | no | <0 | no | 51.13; 5.2; 52.1 |
| 11-20 CPD | 2 | 372 | 0.79 (0.38, 1.65) | | | 0.79 (0.38, 1.65) | 0.5 | | no | <0 | no | 51.14; 54.2 |
| >20 CPD | 2 | 267 | 2.53 (1.00, 6.37) | | | 2.53 (0.69, 9.19) | 0.9 | | no | <0 | no | 51.15; 54.3 |
| Risk estimate |  |  |  | |  |  |  |  |  |  |  |  |
| OR | 5 | 953 | 1.30 (1.10, 1.53) | | | 1.32 (1.01, 1.74) | 0.6 | | no | <0 | no | 51.16; 5.1; 62.1; 54.4; 26.1 |
| Study design |  |  |  | |  |  |  |  |  |  |  |  |
| case-control | 5 | 953 | 1.30 (1.10, 1.53) | | | 1.32 (1.01, 1.74) | 0.6 | | no | <0 | no | 51.16; 5.1; 62.1; 54.4; 26.1 |
| Number of exposed cases |  |  |  | |  |  |  |  |  |  |  |  |
| <50 | 2 | 341 | 1.47 (1.15, 1.89) | | | 1.48 (0.85, 2.58) | 0.6 | | no | <0 | no | 51.16; 54.4 |
| Decade of cancer diagnosis | | |  | |  |  |  |  |  |  |  |  |
| 90-99 | 3 | 842 | 1.29 (1.07, 1.55) | | | 1.33 (0.92, 1.91) | 0.2 | | no | <0 | no | 51.16; 62.1; 54.4 |
| Study region |  |  |  | |  |  |  |  |  |  |  |  |
| Europe | 2 | 341 | 1.47 (1.15, 1.89) | | | 1.48 (0.85, 2.58) | 0.6 | | no | <0 | no | 51.16; 54.4 |
| North America | 3 | 612 | 1.18 (0.95, 1.47) | | | 1.20 (0.93, 1.53) | 0.7 | | no | <0 | no |  |
| Study country |  |  |  | |  |  |  |  |  |  |  |  |
| Germany | 2 | 341 | 1.47 (1.15, 1.89) | | | 1.48 (0.85, 2.58) | 0.6 | | no | <0 | no | 51.16; 54.4 |
| US | 3 | 612 | 1.18 (0.95, 1.47) | | | 1.20 (0.93, 1.53) | 0.7 | | no | <0 | no | 5.1; 62.1; 26.1 |
| Age at cancer diagnosis | | |  | |  |  |  |  |  |  |  |  |
| >14 | 2 | 660 | 1.26 (1.01, 1.57) | | | 1.31 (0.82, 2.09) | 0.1 | | yes | 0.6 | substantial | 51.16; 62.1 |
| Exposure assessment | | |  | |  |  |  |  |  |  |  |  |
| interview | 3 | 612 | 1.18 (0.95, 1.47) | | | 1.20 (0.93, 1.53) | 0.7 | | no | <0 | no | 5.1; 62.1; 26.1 |
| questionnaire | 2 | 341 | 1.47 (1.15, 1.89) | | | 1.48 (0.85, 2.58) | 0.6 | | no | <0 | no | 51.16; 54.4 |
| Adjustment of risk estimate | | |  | |  |  |  |  |  |  |  |  |
| yes | 2 | 683 | 1.19 (0.96, 1.48) | | | 1.21 (0.87, 1.68) | 0.3 | | no | <0 | no | 62.1; 54.4 |
| no | 3 | 270 | 1.45 (1.13, 1.86) | | | 1.42 (0.93, 2.17) | 0.7 | | no | <0 | no | 51.16; 5.1; 26.1 |

#### Table F. Subgroup analyses of risk for bone cancer in early life due to maternal smoking

|  | Number of studies | | Included cases | | Fixed effect model  (95%CI) | | | Random effect model  (95%CI) | | Heterogeneity | | | | | Included datasets |
| --- | --- | --- | --- | --- | --- | --- | --- | --- | --- | --- | --- | --- | --- | --- | --- |
|  |  |  |  |  |  |  |  |  |  | Q (p-value) | Inter- pretation | I^2^ | | Inter- pretation |  |
| Time of exposure | |  | |  | |  |  |  |  |  |  |  |  | |  |
| During pregnancy | | 2 | | 138 | | 0.79 (0.50, 1.26) | | 0.79 (0.50, 1.26) | | 0.3 | no | 0.2 | no | | 51.23; 20.1 |
| Study design | |  | |  | |  |  |  |  |  |  |  |  | |  |
| case-control | | 2 | | 138 | | 0.79 (0.50, 1.26) | | 0.79 (0.50, 1.26) | | 0.3 | no | 0.2 | no | | 51.23; 20.1 |
| Number of exposed cases | | | | | |  |  |  |  |  |  |  |  | |  |
| <50 | | 2 | | 138 | | 0.79 (0.50, 1.26) | | 0.79 (0.50, 1.26) | | 0.3 | no | 0.2 | no | | 51.23; 20.1 |
| Adjustment of risk estimate | | | | | |  |  |  |  |  |  |  |  | |  |
| no | | 2 | | 138 | | 0.79 (0.50, 1.26) | | 0.79 (0.50, 1.26) | | 0.3 | no | 0.2 | no | | 51.23; 20.1 |

#### Table G. Subgroup analyses of risk for soft tissue tumors in early life due to maternal smoking

|  | Number of studies | Included cases | | Fixed effect model  (95%CI) | | | Random effect model  (95%CI) | | | Heterogeneity | | | | | Included datasets |
| --- | --- | --- | --- | --- | --- | --- | --- | --- | --- | --- | --- | --- | --- | --- | --- |
|  |  |  |  |  |  |  |  |  |  | Q (p-value) | Inter- pretation | | I^2^ | Inter- pretation |  |
| Time of exposure |  |  |  | |  |  | |  |  | | |  |  |  |  |
| During pregnancy | 5 | 580 | 0.89 (0.71, 1.12) | | | 0.89 (0.71, 1.12) | | | 0.7 | | | no | <0 | no | 51.27; 19.1; 18.1; 30.2; 30.4 |
| Amount of smoking |  |  |  | |  |  | |  |  | | |  |  |  |  |
| <21 CPD | 2 | 88 | 0.82 (0.49, 1.36) | | | 0.82 (0.49, 1.36) | | | 0.5 | | | no | <0 | no | 30.1; 30.3 |
| Risk estimate |  |  |  | |  |  | |  |  | | |  |  |  |  |
| OR | 4 | 547 | 0.88 (0.70, 1.12) | | | 0.88 (0.70, 1.12) | | | 0.6 | | | no | <0 | no | 51.27; 19.1; 30.2; 30.4 |
| Study design |  |  |  | |  |  | |  |  | | |  |  |  |  |
| case-control | 5 | 580 | 0.89 (0.71, 1.12) | | | 0.89 (0.71, 1.12) | | | 0.7 | | | no | <0 | no | 51.27; 19.1; 18.1; 30.2; 30.4 |
| Number of exposed cases | | |  | |  |  | |  |  | | |  |  |  |  |
| <50 | 4 | 258 | 0.80 (0.58, 1.10) | | | 0.80 (0.58, 1.10) | | | 0.8 | | | no | <0 | no | 51.27; 18.1; 30.2; 30.4 |
| Decade of cancer diagnosis | | |  | |  |  | |  |  | | |  |  |  |  |
| 80-89 | 3 | 410 | 0.89 (0.68, 1.17) | | | 0.88 (0.65, 1.19) | | | 0.4 | | | no | <0 | no | 19.1; 30.2; 30.4 |
| Study region |  |  |  | |  |  | |  |  | | |  |  |  |  |
| Europe | 3 | 225 | 0.78 (0.55, 1.09) | | | 0.77 (0.54, 1.10) | | | 0.6 | | | no | <0 | no | 51.27; 30.2; 30.4 |
| North America | 2 | 355 | 1.0 (0.73, 1.35) | | | 1.0 (0.73, 1.35) | | | 1 | | | no | / | no | 19.1; 18.1 |
| Study country |  |  |  | |  |  | |  |  | | |  |  |  |  |
| Italy | 2 | 88 | 0.69 (0.41, 1.14) | | | 0.69 (0.39, 1.20) | | | 0.5 | | | no | <0 | no | 30.2; 30.4 |
| US | 2 | 355 | 1.0 (0.73, 1.35) | | | 1.0 (0.73, 1.35) | | | 1 | | | no | / | no | 19.1; 18.1 |
| Age at cancer diagnosis | | |  | |  |  | |  |  | | |  |  |  |  |
| <15 | 3 | 121 | 0.75 (0.48, 1.17) | | | 0.75 (0.48, 1.17) | | | 0.6 | | | no | <0 | no | 18.1; 30.2; 30.4 |
| >14 | 2 | 459 | 0.95 (0.73, 1.23) | | | 0.95 (0.73, 1.23) | | | 0.6 | | | no | <0 | no | 51.27; 19.1 |
| Exposure assessment | | |  | |  |  | |  |  | | |  |  |  |  |
| interview | 4 | 443 | 0.90 (0.70, 1.17) | | | 0.90 (0.70, 1.17) | | | 0.6 | | | no | <0 | no | 19.1; 18.1; 30.2; 30.4 |
| Adjustment of risk estimate | | |  | |  |  | |  |  | | |  |  |  |  |
| yes | 3 | 410 | 0.89 (0.68, 1.17) | | | 0.88 (0.65, 1.19) | | | 0.4 | | | no | 0.0 | no | 19.1; 30.2; 30.4 |
| no | 2 | 170 | 0.88 (0.59, 1.32) | | | 0.88 (0.59, 1.32) | | | 0.8 | | | no | <0 | no | 51.27; 18.1 |

#### Table H. Subgroup analyses of risk for renal tumors in early life due to maternal smoking

|  | Number of studies | | Included cases | | | Fixed effect model  (95%CI) | | | Random effect model  (95%CI) | | Heterogeneity | | | | | | Included datasets |
| --- | --- | --- | --- | --- | --- | --- | --- | --- | --- | --- | --- | --- | --- | --- | --- | --- | --- |
|  |  |  |  |  |  |  |  |  |  |  | Q (p-value) | Inter- pretation | | I^2^ | Inter- pretation | |  |
| Time of exposure | |  | |  |  | |  |  | |  |  | |  |  |  |  | |
| During pregnancy | | 3 | | 513 | 0.95 (0.75, 1.22) | | | 0.95 (0.75, 1.22) | | | 0.9 | | no | <0 | no | 51.19; 53.3; 40.3 | |
| Amount of smoking | |  | |  |  | |  |  | |  |  | |  |  |  |  | |
| <11 CPD | | 3 | | 469 | 0.86 (0.64, 1.16) | | | 0.86 (0.64, 1.16) | | | 0.9 | | no | <0 | no | 51.17; 53.1; 40.1 | |
| >10 CPD | | 2 | | 330 | 0.86 (0.56, 1.33) | | | 0.86 (0.56, 1.33) | | | 0.3 | | no | 0.1 | no | 53.2; 40.2 | |
| Risk estimate | |  | |  |  | |  |  | |  |  | |  |  |  |  | |
| OR | | 3 | | 513 | 0.95 (0.75, 1.22) | | | 0.95 (0.75, 1.22) | | | 0.9 | | no | <0 | no | 51.19; 53.3; 40.3 | |
| Study design | |  | |  |  | |  |  | |  |  | |  |  |  |  | |
| case-control | | 3 | | 513 | 0.95 (0.75, 1.22) | | | 0.95 (0.75, 1.22) | | | 0.9 | | no | <0 | no | 51.19; 53.3; 40.3 | |
| Number of exposed cases | | | | |  | |  |  | |  |  | |  |  |  |  | |
| <50 | | 3 | | 513 | 0.95 (0.75, 1.22) | | | 0.95 (0.75, 1.22) | | | 0.9 | | no | <0 | no | 51.19; 53.3; 40.3 | |
| Decade of cancer diagnosis | | | | |  | |  |  | |  |  | |  |  |  |  | |
| 90-99 | | 2 | | 318 | 0.97 (0.73, 1.29) | | | 0.97 (0.73, 1.29) | | | 0.9 | | no | <0 | no | 51.19; 53.3 | |
| Study region | |  | |  |  | |  |  | |  |  | |  |  |  |  | |
| Europe | | 2 | | 318 | 0.97 (0.73, 1.29) | | | 0.97 (0.73, 1.29) | | | 0.9 | | no | <0 | no | 51.19; 53.3 | |
| Study country | |  | |  |  | |  |  | |  |  | |  |  |  |  | |
| Germany | | 2 | | 318 | 0.97 (0.73, 1.29) | | | 0.97 (0.73, 1.29) | | | 0.9 | | no | <0 | no | 51.19; 53.3 | |
| Age at cancer diagnosis | | | | |  | |  |  | |  |  | |  |  |  |  | |
| >14 | | 2 | | 340 | 0.95 (0.70, 1.31) | | | 0.95 (0.70, 1.31) | | | 0.8 | | no | <0 | no | 51.19; 40.3 | |
| Exposure assessment | |  | |  |  | |  |  | |  |  | |  |  |  |  | |
| questionnaire | | 2 | | 318 | 0.97 (0.73, 1.29) | | | 0.97 (0.73, 1.29) | | | 0.9 | | no | <0 | no | 51.19; 53.3 | |
| Adjustment of risk estimate | | | | |  | |  |  | |  |  | |  |  |  |  | |
| yes | | 2 | | 386 | 0.94 (0.70, 1.26) | | | 0.94 (0.70, 1.26) | | | 0.9 | | no | <0 | no | 53.3; 40.3 | |

#### Table I. Subgroup analyses of risk for hepatic tumors in early life due to maternal smoking

|  | | Number of studies | Included cases | | Fixed effect model  (95%CI) | | | Random effect model  (95%CI) | | Heterogeneity | | | | | Included datasets |
| --- | --- | --- | --- | --- | --- | --- | --- | --- | --- | --- | --- | --- | --- | --- | --- |
|  |  |  |  |  |  |  |  |  |  | Q (p-value) | Inter- pretation | | I^2^ | Inter- pretation |  |
| Time of exposure |  | |  |  | |  |  | |  |  | |  |  |  |  |
| During pregnancy | 3 | | 535 | 0.97 (0.70, 1.36) | | | 0.97 (0.70, 1.36) | | | 0.9 | | no | <0 | no | 25.1; 11.1; 6.4 |
| Risk estimate |  | |  |  | |  |  | |  |  | |  |  |  |  |
| OR | 3 | | 535 | 0.97 (0.70, 1.36) | | | 0.97 (0.70, 1.36) | | | 0.9 | | no | <0 | no | 25.1; 11.1; 6.4 |
| Study design |  | |  |  | |  |  | |  |  | |  |  |  |  |
| case-control | 3 | | 535 | 0.97 (0.70, 1.36) | | | 0.97 (0.70, 1.36) | | | 0.9 | | no | <0 | no | 25.1; 11.1; 6.4 |
| Number of exposed cases | | | |  | |  |  | |  |  | |  |  |  |  |
| <50 | 3 | | 535 | 0.97 (0.70, 1.36) | | | 0.97 (0.70, 1.36) | | | 0.9 | | no | <0 | no | 25.1; 11.1; 6.4 |
| Study region |  | |  |  | |  |  | |  |  | |  |  |  |  |
| North America | 2 | | 443 | 0.96 (0.64, 1.45) | | | 0.96 (0.64, 1.45) | | | 0.6 | | no | <0 | no | 25.1; 6.4 |
| Study country |  | |  |  | |  |  | |  |  | |  |  |  |  |
| Multi | 2 | | 157 | 1.04 (0.66, 1.63) | | | 1.04 (0.66, 1.63) | | | 0.8 | | no | <0 | no | 11.1; 6.4 |
| Exposure assessment | | | |  | |  |  | |  |  | |  |  |  |  |
| interview | 2 | | 443 | 0.96 (0.64, 1.45) | | | 0.96 (0.64, 1.45) | | | 0.6 | | no | <0 | no | 25.1; 6.4 |
| Adjustment of risk estimate | | | |  | |  |  | |  |  | |  |  |  |  |
| no | 2 | | 157 | 1.04 (0.66, 1.63) | | | 1.04 (0.66, 1.63) | | | 0.8 | | no | <0 | no | 11.1; 6.4 |

#### Table J. Subgroup analyses of risk for germ cell tumors in early life due to maternal smoking

|  | | Number of studies | Included cases | | Fixed effect model  (95%CI) | | | Random effect model  (95%CI) | | | Heterogeneity | | | | | Included datasets |
| --- | --- | --- | --- | --- | --- | --- | --- | --- | --- | --- | --- | --- | --- | --- | --- | --- |
|  |  |  |  |  |  |  |  |  |  |  | Q (p-value) | Inter- pretation | | I^2^ | Inter- pretation |  |
| Time of exposure |  | |  |  | |  |  | |  |  | | |  |  |  |  |
| During pregnancy | 4 | | 641 | 0.97 (0.78, 1.19) | | | 0.92 (0.68, 1.24) | | | 0.1 | | | yes | 0.5 | moderate | 60.1; 44.1; 9.9; 56.4 |
| 3 months before pregnancy | 2 | | 393 | 0.85 (0.65, 1.12) | | | 0.80 (0.48, 1.31) | | | 0.1 | | | yes | 0.6 | substantial | 9.1; 56.1 |
| Risk estimate |  | |  |  | |  |  | |  |  | | |  |  | moderate |  |
| OR | 4 | | 641 | 0.97 (0.78, 1.19) | | | 0.92 (0.68, 1.24) | | | 0.1 | | | yes | 0.5 | moderate | 60.1; 44.1; 9.9; 56.4 |
| Study design |  | |  |  | |  |  | |  |  | | |  |  |  |  |
| case-control | 4 | | 641 | 0.97 (0.78, 1.19) | | | 0.92 (0.68, 1.24) | | | 0.1 | | | yes | 0.5 | moderate | 60.1; 44.1; 9.9; 56.4 |
| Number of exposed cases | | | |  | |  |  | |  |  | | |  |  |  |  |
| <50 | 2 | | 175 | 0.67 (0.44, 1.00) | | | 0.67 (0.37, 1.19) | | | 0.5 | | | no | <0 | no | 60.1; 56.4 |
| <100 | 2 | | 466 | 1.10 (0.87, 1.40) | | | 1.10 (0.81, 1.51) | | | 0.1 | | | yes | 0.6 | substantial | 44.1; 9.9 |
| Decade of cancer diagnosis | | | |  | |  |  | |  |  | | |  |  |  |  |
| 80-89 | 2 | | 297 | 0.80 (0.60, 1.07) | | | 0.75 (0.45, 1.27) | | | 0.2 | | | no | 0.4 | moderate | 44.1; 56.4 |
| 90-99 | 2 | | 344 | 1.17 (0.87, 1.58) | | | 1.14 (0.80, 1.63) | | | 0.1 | | | yes | 0.5 | moderate | 60.1; 9.9 |
| Study region |  | |  |  | |  |  | |  |  | | |  |  |  |  |
| Europe | 2 | | 262 | 0.87 (0.65, 1.17) | | | 0.87 (0.65, 1.17) | | | 0.7 | | | no | <0 | no | 60.1; 44.1 |
| Study country |  | |  |  | |  |  | |  |  | | |  |  |  |  |
| Multi | 2 | | 175 | 0.67 (0.44, 1.00) | | | 0.67 (0.37, 1.19) | | | 0.5 | | | no | <0 | no | 60.1; 56.4 |
| Age at cancer diagnosis | | | |  | |  |  | |  |  | | |  |  |  |  |
| <15 | 3 | | 571 | 0.99 (0.80, 1.24) | | | 0.94 (0.66, 1.36) | | | 0.03 | | | yes | 0.7 | substantial | 44.1; 9.9; 56.4 |
| Adjustment of risk estimate | | | |  | |  |  | |  |  | | |  |  |  |  |
| yes | 3 | | 571 | 0.99 (0.80, 1.24) | | | 0.94 (0.66, 1.36) | | | 0.03 | | | yes | 0.7 | substantial | 44.1; 9.9; 56.4 |

#### Table K. Subgroup analyses of risk for leukemia in early life due to maternal smoking

|  | Number of studies | | Included cases | Fixed effect model  (95%CI) | | Random effect model  (95%CI) | | Heterogeneity | | | | | Included datasets  (numbers refer to table S2) |
| --- | --- | --- | --- | --- | --- | --- | --- | --- | --- | --- | --- | --- | --- |
|  |  |  |  |  |  |  |  | Q (p-value) | | Inter- pretation | I^2^ | Inter- pretation |  |
| Time of exposure | | |  |  |  |  |  | |  |  |  |  |  |
| During pregnancy | 22 | | 14,711 | 0.92 (0.86, 0.97) | | 0.92 (0.82, 1.03) | | 0.0002 | | yes | 0.5 | moderate | 35.1; 59.1; 49.7; 37.7; 58.3; 51.4; 33.1; 27.1; 42.1; 13.45; 48.1; 1.1; 61.1; 46.1; 41.1; 55.1; 2.1; 29.5; 50.1; 39.3; 8.1; 57.2 |
| 3 months before pregnancy | 4 | | 418 | 0.96 (0.85, 1.08) | | 0.95 (0.83, 1.09) | | 0.3 | | no | 0.1 | no | 13.46; 3.1; 3.6; 57.1 |
| 1st trimester | 8 | | 2130 | 0.97 (0.88, 1.07) | | 0.97 (0.87, 1.08) | | 0.3 | | no | 0.1 | no | 12.3; 23.3; 13.47; 41.6; 31.1; 3.2; 3.7; 57.3 |
| 2nd trimester | 6 | | 1626 | 0.97 (0.87, 1.08) | | 0.96 (0.84, 1.09) | | 0.2 | | no | 0.2 | no | 23.6; 13.48; 41.7; 3.3; 3.8; 57.4 |
| 3rd trimester | 6 | | 1622 | 0.97 (0.87, 1.08) | | 0.97 (0.86, 1.09) | | 0.3 | | no | 0.2 | no | 23.9; 13.49; 41.8; 3.4; 3.9; 57.5 |
| breastfeeding (3 months after pregnancy) | 4 | | 418 | 0.82 (0.69, 0.99) | | 0.74 (0.52, 1.05) | | 0.1 | | yes | 0.5 | moderate | 13.50; 3.5; 3.10; 57.6 |
| Amount of smoking | | |  |  |  |  |  | |  |  |  |  |  |
| <11 CPD | 7 | | 3946 | 0.78 (0.70, 0.87) | | 0.80 (0.60, 1.05) | | 0.0002 | | yes | 0.7 | substantial | 35.2; 49.8; 37.8; 51.1; 33.2; 41.4; 57.7 |
| 11-20 CPD | 2 | | 1119 | 0.56 (0.39, 0.81) | | 0.56 (0.25, 1.26) | | 0.5 | | no | <0 | no | 51.2; 57.8 |
| >10 CPD | 4 | | 2043 | 0.56 (0.47, 0.68) | | 0.65 (0.34, 1.24) | | 0.0004 | | yes | 0.8 | considerable | 35.3; 49.9; 37.9; 41.5 |
| >20 CPD | 2 | | 1100 | 0.84 (0.37, 1.89) | | 0.84 (0.37, 1.89) | | 0.4 | | no | <0 | no | 51.3; 57.9 |
| Risk estimate |  | |  |  |  |  |  | |  |  |  |  |  |
| OR | 21 | | 14,158 | 0.92 (0.86, 0.98) | | 0.92 (0.82, 1.04) | | 0.0001 | | yes | 0.6 | substantial | 35.1; 59.1; 22.1; 58.3; 51.4; 33.1; 27.1; 42.1; 13.45; 48.1; 1.1; 61.1; 46.1; 41.1; 55.1; 2.1; 29.5; 50.1; 39.3; 8.1; 57.2 |
| Study design |  | |  |  |  |  |  | |  |  |  |  |  |
| case-control | 21 | | 14,158 | 0.92 (0.86, 0.98) | | 0.92 (0.82, 1.04) | | 0.0001 | | yes | 0.6 | substantial | 35.1; 59.1; 22.1; 58.3; 51.4; 33.1; 27.1; 42.1; 13.45; 48.1; 1.1; 61.1; 46.1; 41.1; 55.1; 2.1; 29.5; 50.1; 39.3; 8.1; 57.2 |
| Number of exposed cases | | | |  |  |  |  | |  |  |  |  |  |
| <50 | | 7 | 923 | 1.06 (0.85, 1.34) | | 1.07 (0.77, 1.50) | | 0.04 | | yes | 0.5 | moderate | 27.1; 13.45; 1.1; 61.1; 2.1; 50.1; 8.1 |
| <100 | | 8 | 8256 | 0.91 (0.81, 1.02) | | 0.91 (0.78, 1.07) | | 0.1 | | yes | 0.4 | moderate | 35.1; 59.1; 33.1; 42.1; 48.1; 46.1; 29.5; 39.3 |
| <200 | | 3 | 2477 | 0.80 (0.71, 0.90) | | 0.81 (0.59, 1.11) | | 0.01 | | yes | 0.7 | substantial | 49.7; 51.4; 41.1 |
| Decade of cancer diagnosis | | | |  |  |  |  | |  |  |  |  |  |
| 70-79 | | 2 | 224 | 0.77 (0.55, 1.07) | | 0.74 (0.44, -1.24) | | 0.3 | | no | 0.1 | no | 61.1; 29.5 |
| 80-89 | | 3 | 557 | 0.85 (0.69, 1.04) | | 0.86 (0.63, 1.18) | | 0.1 | | yes | 0.5 | moderate | 48.1; 55.1; 57.2 |
| 90-99 | | 8 | 10,842 | 0.96 (0.88, 1.04) | | 0.94 (0.79, 1.11) | | 0.003 | | yes | 0.6 | substantial | 54.1; 58.3; 51.4; 33.1; 1.1; 46.1; 50.1; 39.3 |
| 00-09 | | 6 | 2694 | 0.84 (0.75, 0.94) | | 0.88 (0.70, 1.10) | | 0.01 | | yes | 0.6 | substantial | 35.1; 59.1; 22.1; 27.1; 13.45; 41.1 |
| Study region | |  |  |  |  |  |  | |  |  |  |  |  |
| Europe | | 9 | 4149 | 0.88 (0.80, 0.96) | | 0.92 (0.77, 1.10) | | 0.002 | | yes | 0.6 | substantial | 49.7; 37.7; 51.4; 33.1; 27.1; 42.1; 61.1; 41.1; 29.5 |
| North America | | 6 | 7223 | 0.84 (0.73, 0.97) | | 0.90 (0.69, 1.17) | | 0.04 | | yes | 0.5 | moderate | 59.1; 48.1; 46.1; 55.1; 50.1; 39.3 |
| South America | | 2 | 201 | 0.79 (0.49, 1.27) | | 0.79 (0.49, 1.27) | | 0.5 | | no | <0 | no | 13.45; 8.1 |
| Multi | | 3 | 2638 | 1.03 (0.92, 1.16) | | 0.98 (0.70, 1.37) | | 0.02 | | yes | 0.7 | substantial | 58.3; 2.1; 57.2 |
| Study country | |  |  |  |  |  |  | |  |  |  |  |  |
| France | | 3 | 1965 | 0.85 (0.75, 0.97) | | 0.92 (0.66, 1.30) | | 0.0002 | | yes | 0.8 | considerable | 49.7; 33.1; 41.1 |
| Greece | | 2 | 405 | 1.24 (0.92, 1.66) | | 1.24 (0.90, 1.71) | | 0.7 | | no | <0 | no | 27.1; 42.1 |
| US | | 4 | 6780 | 0.81 (0.68, 0.96) | | 0.89 (0.62, 1.29) | | 0.03 | | yes | 0.6 | substantial | 48.1; 46.1; 50.1; 39.3 |
| Multi | | 4 | 2638 | 1.04 (0.93, 1.17) | | 1.02 (0.79, 1.33) | | 0.03 | | yes | 0.6 | substantial | 58.3; 55.1; 2.1; 57.2 |
| Age at cancer diagnosis | | | |  |  |  |  | |  |  |  |  |  |
| <6 | | 5 | 1550 | 0.84 (0.73, 0.98) | | 0.85 (0.66, 1.09) | | 0.1 | | yes | 0.4 | moderate | 59.1; 37.7; 13.45; 2.1; 57.2 |
| <15 | | 13 | 6666 | 0.96 (0.89, 1.03) | | 0.97 (0.84, 1.12) | | 0.0003 | | yes | 0.6 | substantial | 35.1; 49.7; 58.3; 51.4; 33.1; 27.1; 42.1; 48.1; 61.1; 41.1; 29.5; 50.1; 8.1 |
| >14 | | 3 | 6383 | 0.80 (0.67, 0.97) | | 0.83 (0.59, 1.17) | | 0.1 | | yes | 0.5 | moderate | 46.1; 55.1; 39.3 |
| Exposure assessment | | | |  |  |  |  | |  |  |  |  |  |
| birth register | | 3 | 6936 | 0.80 (0.70, 0.93) | | 0.78 (0.58, 1.05) | | 0.4 | | no | 0.0 | no | 54.1; 46.1; 39.3 |
| interview | | 14 | 5600 | 0.95 (0.87, 1.03) | | 0.97 (0.82, 1.13) | | 0.0003 | | yes | 0.6 | substantial | 59.1; 49.7; 58.3; 27.1; 42.1; 13.45; 1.1; 41.1; 55.1; 2.1; 29.5; 50.1; 8.1; 57.2 |
| questionnaire | | 5 | 2175 | 0.93 (0.82, 1.07) | | 0.93 (0.77, 1.13) | | 0.1 | | yes | 0.5 | moderate | 35.1; 51.4; 33.1; 48.1; 61.1 |
| Adjustment of risk estimate | | | |  |  |  |  | |  |  |  |  |  |
| yes | | 12 | 4759 | 0.85 (0.78, 0.93) | | 0.86 (0.74, 1.00) | | 0.01 | | yes | 0.5 | moderate | 35.1; 59.1; 49.7; 37.7; 33.1; 42.1; 13.45; 61.1; 46.1; 41.1; 29.5; 57.2 |
| no | | 10 | 9952 | 1.00 (0.91, 1.10) | | 1.01 (0.85, 1.20) | | 0.02 | | yes | 0.5 | moderate | 58.3; 51.4; 27.1; 48.1; 1.1; 55.1; 2.1; 50.1; 39.3; 8.1 |

#### Table K.a. Subgroup analyses of risk for acute lymphoblastic leukemia in early life due to maternal smoking

|  | Number of studies | Included cases | Fixed effect model  (95%CI) | | Random effect model  (95%CI) | Heterogeneity | | | | | | | Included datasets |
| --- | --- | --- | --- | --- | --- | --- | --- | --- | --- | --- | --- | --- | --- |
|  |  |  |  |  |  | Q (p-value) | | | | Inter- pretation | I^2^ | Inter- pretation |  |
| Time of exposure | |  |  |  |  | | |  |  |  |  |  |  |
| During pregnancy | 19 | 11,891 | 0.99 (0.92, 1.06) | | 0.99 (0.87, 1.13) | | 0.0003 | | | yes | 0.6 | substantial | 35.1; 49.1; 37.1; 58.2; 33.4; 27.1; 13.1; 13.13; 48.1; 1.1; 46.2; 41.9; 2.2; 24.22; 29.1; 50.1; 39.1; 8.1; 57.11 |
| 3 months before pregnancy | 5 | 435 | 1.00 (0.84, 1.20) | | 1.03 (0.83, 1.27) | | 0.2 | | | no | 0.2 | no | 13.2; 13.14; 24.4; 3.1; 57.10 |
| 1st trimester | 8 | 1948 | 1.03 (0.92, 1.16) | | 1.05 (0.90, 1.21) | | 0.2 | | | no | 0.2 | no | 12.3; 23.3; 13.3; 13.15; 41.14; 24.13; 3.2; 57.12 |
| 2nd trimester | 6 | 1498 | 1.00 (0.88, 1.15) | | 1.00 (0.88, 1.15) | | 0.6 | | | no | <0 | no | 23.6; 13.4; 13.16; 41.15; 3.3; 57.13 |
| 3rd trimester | 6 | 1499 | 1.02 (0.89, 1.16) | | 1.02 (0.89, 1.16) | | 6 | | | no | <0 | no | 23.9; 13.5; 13.17; 41.16; 3.4; 57.14 |
| breastfeeding | 4 | 396 | 0.89 (0.70, 1.13) | | 0.90 (0.58, 1.39) | | 0.1 | | | yes | 0.6 | substantial | 13.6; 13.18; 3.5; 57.15 |
| Amount of smoking |  |  |  |  |  | | |  |  |  |  |  |  |
| <11 CPD | 7 | 2614 | 1.03 (0.91, 1.16) | | 1.03 (0.85, 1.27) | | 0.01 | | | yes | 0.6 | substantial | 35.2; 49.2; 37.2; 33.5; 41.12; 24.23; 57.16 |
| >10 CPD | 6 | 1964 | 0.97 (0.81, 1.17) | | 1.00 (0.80, 1.26) | | 0.2 | | | no | 0.3 | no | 35.3; 49.3; 37.3; 41.13; 24.24; 29.3 |
| Risk estimate |  |  |  |  |  | | |  |  |  |  |  |  |
| OR | 18 | 11,386 | 1.03 (0.95, 1.11) | | 1.02 (0.89, 1.16) | | 0.002 | | | yes | 0.5 | moderate | 35.1; 49.1; 58.2; 33.4; 27.1; 13.1; 13.13; 48.1; 1.1; 46.2; 41.9; 2.2; 24.22; 29.1; 50.1; 39.1; 8.1; 57.11 |
| Study design |  |  |  |  |  | | |  |  |  |  |  |  |
| case-control | 18 | 11,386 | 1.03 (0.95, 1.11) | | 1.02 (0.89, 1.16) | | 0.002 | | | yes | 0.5 | moderate | 35.1; 49.1; 58.2; 33.4; 27.1; 13.1; 13.13; 48.1; 1.1; 46.2; 41.9; 2.2; 24.22; 29.1; 50.1; 39.1; 8.1; 57.11 |
| Number of exposed cases | | |  |  |  | | |  |  |  |  |  |  |
| <50 | 9 | 6733 | 0.94 (0.77, 1.13) | | 1.06 (0.76, 1.48) | | 0.002 | | | yes | 0.6 | moderate | 27.1; 13.1; 13.13; 1.1; 24.22; 29.1; 50.1; 39.1; 8.1 |
| <100 | 4 | 1424 | 0.98 (0.84, 1.14) | | 0.99 (0.80, 1.21) | | 0.1 | | | yes | 0.5 | moderate | 35.1; 33.4; 48.1; 46.2 |
| <150 | 2 | 1140 | 0.85 (0.73, 1.00) | | 0.86 (0.65, 1.14) | | 0.1 | | | yes | 0.6 | substantial | 37.1; 41.9 |
| Decade of cancer diagnosis | | |  |  |  | | |  |  |  |  |  |  |
| 80-89 | 3 | 491 | 0.92 (0.72, 1.17) | | 1.04 (0.65, 1.66) | | 0.003 | | | yes | 0.7 | substantial | 48.1; 24.22; 57.11 |
| 90-99 | 7 | 9058 | 0.95 (0.86, 1.05) | | 0.95 (0.75, 1.20) | | 0.0002 | | | yes | 0.7 | substantial | 37.1; 58.2; 33.4; 1.1; 46.2; 50.1; 39.1 |
| 00-09 | 6 | 2115 | 1.08 (0.95, 1.23) | | 1.08 (0.93, 1.25) | | 0.4 | | | no | 0.1 | no | 35.1; 49.1; 27.1; 13.1; 13.13; 41.9 |
| Study region |  |  |  |  |  | | |  |  |  |  |  |  |
| Europe | 6 | 2586 | 1.00 (0.89, 1.12) | | 1.02 (0.83, 1.24) | | 0.003 | | | yes | 0.7 | substantial | 49.1; 37.1; 33.4; 27.1; 41.9; 29.1 |
| North America | 5 | 6580 | 0.85 (0.72, 1.02) | | 1.05 (0.70, 1.57) | | 0.002 | | | yes | 0.7 | substantial | 48.1; 46.2; 24.22; 50.1; 39.1 |
| Multi | 3 | 1947 | 1.06 (0.93, 1.22) | | 1.04 (0.81, 1.35) | | 0.2 | | | no | 0.4 | moderate | 58.2; 2.2; 57.11 |
| South America | 3 | 278 | 0.96 (0.64, 1.45) | | 0.96 (0.64, 1.45) | | 0.2 | | | no | 0.3 | no | 13.1; 13.13; 8.1 |
| Study country |  |  |  |  |  | | |  |  |  |  |  |  |
| Brazil | 2 | 193 | 0.94 (0.60, 1.45) | | 0.94 (0.55, 1.59) | | 0.1 | | | yes | 0.6 | substantial | 13.1; 13.13 |
| France | 3 | 1687 | 1.15 (1.00, 1.32) | | 1.17 (0.93, 1.47) | | 0.2 | | | no | 0.3 | no | 49.1; 33.4; 41.9 |
| US | 5 | 6580 | 0.85 (0.72, 1.02) | | 1.05 (0.70, 1.57) | | 0.002 | | | yes | 0.7 | substantial | 48.1; 46.2; 24.22; 50.1; 39.1 |
| Multi | 3 | 1947 | 1.06 (0.93, 1.22) | | 1.04 (0.81, 1.35) | | 0.2 | | | no | 0.4 | moderate | 58.2; 2.2; 57.11 |
| Age at cancer diagnosis | | |  |  |  | | |  |  |  |  |  |  |
| <15 | 11 | 4728 | 1.09 (1.00. 1.19) | | 1.12 (0.96, 1.30) | | 0.04 | | | yes | 0.4 | moderate | 35.1; 49.1; 58.2; 33.4; 27.1; 48.1; 41.9; 24.22; 29.1; 50.1; 8.1 |
| <6 | 5 | 901 | 0.82 (0.69, 0.97) | | 0.89 (0.66, 1.20) | | 0.1 | | | yes | 0.4 | moderate | 37.1; 13.1; 13.13; 2.2; 57.11 |
| >14 | 2 | 6150 | 0.72 (0.58, 0.90) | | 0.71 (0.42, 1.18) | | 0.3 | | | no | 0.1 | no | 46.2; 39.1 |
| Exposure assessment |  |  |  |  |  | | |  |  |  |  |  |  |
| birth register | 3 | 6655 | 0.74 (0.63, 0.86) | | 0.72 (0.50, 1.04) | | 0.6 | | | no | <0 | no | 37.1; 46.2; 39.1 |
| interview | 13 | 4188 | 1.08 (0.98, 1.18) | | 1.09 (0.92, 1.28) | | 0.004 | | | yes | 0.4 | moderate | 49.1; 58.2; 27.1; 13.1; 13.13; 1.1; 41.9; 2.2; 24.22; 29.1; 50.1; 8.1; 57.11 |
| questionnaire | 3 | 1048 | 1.06 (0.89, 1.27) | | 1.07 (0.84, 1.35) | | 0.1 | | | yes | 0.5 | moderate | 35.1; 33.4; 48.1 |
| Adjustment of risk estimate | | |  |  |  | | |  |  |  |  |  |  |
| yes | 10 | 3494 | 0.95 (0.86, 1.04) | | 0.94 (0.81, 1.10) | | 0.01 | | | yes | 0.5 | moderate | 35.1; 49.1; 37.1; 33.4; 13.1; 13.13; 46.2; 41.9; 29.1; 57.11 |
| no | 9 | 8392 | 1.05 (0.94, 1.18) | | 1.13 (0.87, 1.45) | | 0.005 | | | yes | 0.6 | substantial | 58.2; 27.1; 48.1; 1.1; 2.2; 24.22; 50.1; 39.1; 8.1 |

#### Table K.b. Subgroup analyses of risk for acute myeloblastic leukemia in early life due to maternal smoking

|  | Number of studies | | Included cases | Fixed effect model  (95%CI) | | | Random effect model  (95%CI) | Heterogeneity | | | | | | Included datasets |
| --- | --- | --- | --- | --- | --- | --- | --- | --- | --- | --- | --- | --- | --- | --- |
|  |  |  |  |  |  |  |  | Q (p-value) | | | Inter- pretation | I^2^ | Inter- pretation |  |
| Time of exposure | | |  | |  |  |  | |  |  |  |  |  |  |
| During pregnancy | 12 | | 6739 | | 0.98 (0.83, 1.14) | | 0.95 (0.79, 1.15) | 0.2 | | | no | 0.2 | no | 49.4; 37.4; 58.1; 33.7; 13.7; 13.19; 46.3; 41.17; 55.1; 2.3; 39.2; 57.20 |
| 3 months before pregnancy | 4 | | 147 | | 0.96 (0.82, 1.12) | | 0.75 (0.47, 1.18) | 0.1 | | | yes | 0.4 | moderate | 13.8; 13.20; 3.6; 57.19 |
| 1st trimester | 5 | | 247 | | 0.90 (0.77, 1.06) | | 0.78 (0.56, 1.09) | 0.3 | | | no | 0.2 | no | 13.9; 13.21; 41.22; 3.7; 57.21 |
| 2nd trimester | 5 | | 243 | | 0.93 (0.78, 1.10) | | 0.81 (0.58, 1.11) | 0.3 | | | no | 0.2 | no | 13.10; 13.22; 41.23; 3.8; 57.22 |
| 3rd trimester | 5 | | 243 | | 0.92 (0.78, 1.09) | | 0.82 (0.61, 1.11) | 0.3 | | | no | 0.1 | no | 13.11; 13.23; 41.24; 3.9; 57.23 |
| breastfeeding | 4 | | 147 | | 0.89 (0.70, 1.15) | | 0.89 (0.70, 1.15) | 0.5 | | | no | <0 | no | 13.12; 13.24; 3.10; 57.24 |
| Amount of smoking |  | |  | |  |  |  | |  |  |  |  |  |  |
| <11 CPD | 6 | | 469 | | 0.92 (0.69, 1.23) | | 0.83 (0.55, 1.26) | 0.1 | | | yes | 0.4 | moderate | 49.5; 37.5; 33.8; 41.18; 41.20; 57.25 |
| >10 CPD | 3 | | 209 | | 1.42 (0.84, 2.40) | | 1.40 (0.80, 2.46) | 0.3 | | | no | 0.2 | no | 49.6; 37.6; 41.21 |
| Risk estimate |  | |  | |  |  |  | |  |  |  |  |  |  |
| OR | 11 | | 6691 | | 0.96 (0.81, 1.13) | | 0.92 (0.76, 1.13) | 0.2 | | | no | 0.2 | no | 49.4; 58.1; 33.7; 13.7; 13.19; 46.3; 41.17; 55.1; 2.3; 39.2; 57.20 |
| Study design |  | |  | |  |  |  | |  |  |  |  |  |  |
| case-control | 11 | | 6691 | | 0.96 (0.81, 1.13) | | 0.92 (0.76, 1.13) | 0.2 | | | no | 0.2 | no | 49.4; 58.1; 33.7; 13.7; 13.19; 46.3; 41.17; 55.1; 2.3; 39.2; 57.20 |
| Number of exposed cases | | | | |  |  |  | |  |  |  |  |  |  |
| <50 | | 8 | 6195 | | 0.92 (0.73, 1.16) | | 0.91 (0.71, 1.16) | 0.3 | | | no | 0.1 | no | 49.4; 37.4; 33.7; 13.7; 13.19; 46.3; 41.17; 39.2 |
| Decade of cancer diagnosis | | | | |  |  |  | |  |  |  |  |  |  |
| 80-89 | | 2 | 88 | | 0.93 (0.64, 1.37) | | 0.79 (0.35, 1.74) | 0.03 | | | yes | 0.7 | substantial | 55.1; 57.20 |
| 90-99 | | 5 | 6390 | | 0.97 (0.79, 1.21) | | 0.97 (0.79, 1.21) | 0.5 | | | no | <0 | no | 37.4; 58.1; 33.7; 46.3; 39.2 |
| 00-09 | | 4 | 261 | | 0.95 (0.69, 1.32) | | 0.88 (0.57, 1.36) | 0.1 | | | yes | 0.4 | moderate | 49.4; 13.7; 13.19; 41.17 |
| Study region | |  |  | |  |  |  | |  |  |  |  |  |  |
| Europe | | 4 | 312 | | 1.05 (0.79, 1.39) | | 1.05 (0.79, 1.39) | 0.3 | | | no | 0.2 | no | 49.4; 37.4; 33.7; 41.17 |
| North America | | 3 | 5824 | | 1.00 (0.73, 1.37) | | 1.00 (0.73, 1.37) | 0.5 | | | no | <0 | no | 46.3; 55.1; 39.2 |
| South America | | 2 | 59 | | 0.42 (0.18, 0.98) | | 0.42 (0.13, 1.32) | 0.9 | | | no | <0 | no | 13.7; 13.19 |
| Multi | | 3 | 544 | | 0.98 (0.77, 1.26) | | 0.90 (0.55, 1.47) | 0.1 | | | yes | 0.5 | moderate | 58.1; 2.3; 57.20 |
| Study country | |  |  | |  |  |  | |  |  |  |  |  |  |
| Brazil | | 2 | 59 | | 0.42 (0.18, 0.98) | | 0.42 (0.13, 1.32) | 0.9 | | | no | <0 | no | 13.7; 13.19 |
| France | | 3 | 264 | | 0.99 (0.72, 1.37) | | 0.99 (0.71, 1.38) | 0.2 | | | no | 0.3 | no | 49.4; 33.7; 41.17 |
| US | | 2 | 5824 | | 0.82 (0.52, 1.30) | | 0.82 (0.52, 1.30) | 0.9 | | | no | <0 | no | 46.3; 39.2; |
| Multi | | 4 | 544 | | 1.03 (0.83, 1.28) | | 1.00 (0.72, 1.38) | 0.1 | | | yes | 0.4 | moderate | 58.1; 55.1; 2.3; 57.20 |
| Age at cancer diagnosis | | | | |  |  |  | |  |  |  |  |  |  |
| <6 | | 5 | 195 | | 0.82 (0.57, 1.18) | | 0.76 (0.44, 1.29) | 0.1 | | | yes | 0.5 | moderate | 37.4; 13.7; 13.19; 2.3; 57.20 |
| <15 | | 4 | 720 | | 1.02 (0.83, 1.26) | | 1.02 (0.83, 1.26) | 0.4 | | | no | 0.0 | no | 49.4; 58.1; 33.7; 41.17 |
| >14 | | 3 | 5824 | | 1.00 (0.73, 1.37) | | 1.00 (0.73, 1.37) | 0.5 | | | no | <0 | no | 46.3; 55.1; 39.2 |
| Exposure assessment | |  |  | |  |  |  | |  |  |  |  |  |  |
| birth register | | 3 | 5872 | | 0.96 (0.67, 1.39) | | 0.96 (0.67, 1.39) | 0.5 | | | no | <0 | no | 37.4; 46.3; 39.2 |
| interview | | 8 | 805 | | 1.00 (0.84, 1.20) | | 0.96 (0.75, 1.23) | 0.1 | | | yes | 0.3 | no | 49.4; 58.1; 13.7; 13.19; 41.17; 55.1; 2.3; 57.20 |
| Adjustment of risk estimate | | | | |  |  |  | |  |  |  |  |  |  |
| yes | | 8 | 544 | | 0.86 (0.68, 1.09) | | 0.81 (0.59, 1.10) | 0.1 | | | yes | 0.3 | no | 49.4; 37.4; 33.7; 13.7; 13.19; 46.3; 41.17; 57.20 |
| no | | 4 | 6195 | | 1.08 (0.87, 1.34) | | 1.08 (0.87, 1.34) | 0.8 | | | no | <0 | no | 58.1; 55.1; 2.3; 39.2 |

## Risk of Publication bias

Publication bias was assessed by funnel plots based on Egger’s regression (Figures S1 –S9.b.) [90]. For many of the cancer types the number of included studies was low and therefore evaluating publication bias based on funnel plots complicated.

Figure A. Egger’s plot for lymphoma risk due to any smoking during pregnancy

Figure B. Egger’s plot for Non Hodgkins’ lymphoma risk due to any smoking during pregnancy

Figure C. Egger’s plot for nervous system tumors risk due to any smoking during pregnancy

Figure D. Egger’s plot for neuroblastoma risk due to any smoking during pregnancy

Figure E. Egger’s plot for bone cancer risk due to any smoking during pregnancy

Figure F. Egger’s plot for soft tissue cancer risk due to any smoking during pregnancy

Figure G. Egger’s plot renal cancer risk due to any smoking during pregnancy

Figure H. Egger’s plot for hepatic cancer risk due to any smoking during pregnancy

Figure I. Egger’s plot for germ cell tumors risk due to any smoking during pregnancy

Figure J. Egger’s plot for leukemia risk due to any smoking during pregnancy

Figure K. Egger’s plot for acute lymphoblastic leukemia risk due to any smoking during pregnancy

Figure L. Egger’s plot for acute myeloblastic leukemia risk due to any smoking during pregnancy

# S1.d. References

1. John EM, Savitz DA, Sandler DP. 1991. Prenatal exposure to parents' smoking and childhood cancer. Am J Epidemiol 133(2):123-132.
2. Magnani C, Pastore G, Luzzatto L, Terracini B. 1990. Parental occupation and other environmental factors in the etiology of leukemias and non-hodgkin's lymphomas in childhood: A case-control study. Tumori 76(5):413-419.
3. Mucci LA, Granath F, Cnattingius S. 2004. Maternal smoking and childhood leukemia and lymphoma risk among 1,440,542 swedish children. Cancer Epidemiol Biomarkers Prev 13(9):1528-1533.
4. Petridou ET, Sergentanis TN, Skalkidou A, Antonopoulos CN, Dessypris N, Svensson T et al. 2015. Maternal and birth anthropometric characteristics in relation to the risk of childhood lymphomas: A swedish nationwide cohort study. Eur J Cancer Prev; doi: 10.1097/CEJ.0000000000000122.
5. Rudant J, Menegaux F, Leverger G, Baruchel A, Lambilliotte A, Bertrand Y et al. 2008. Childhood hematopoietic malignancies and parental use of tobacco and alcohol: The ESCALE study (SFCE). Cancer Causes Control 19(10):1277-1290.
6. Schuz J, Kaatsch P, Kaletsch U, Meinert R, Michaelis J. 1999. Association of childhood cancer with factors related to pregnancy and birth. Int J Epidemiol 28(4):631-639.
7. Brooks DR, Mucci LA, Hatch EE, Cnattingius S. 2004. Maternal smoking during pregnancy and risk of brain tumors in the offspring. A prospective study of 1.4 million swedish births. Cancer Causes Control 15(10):997-1005.
8. Buck GM, Michalek AM, Chen CJ, Nasca PC, Baptiste MS. 2001. Perinatal factors and risk of neuroblastoma. Paediatr Perinat Epidemiol 15(1):47-53.
9. Bunin GR, Buckley JD, Boesel CP, Rorke LB, Meadows AT. 1994. Risk factors for astrocytic glioma and primitive neuroectodermal tumor of the brain in young children: A report from the children's cancer group. Cancer Epidemiol Biomarkers Prev 3(3):197-204.
10. Cordier S, Iglesias MJ, Le Goaster C, Guyot MM, Mandereau L, Hemon D. 1994. Incidence and risk factors for childhood brain tumors in the ile de france. Int J Cancer 59(6):776-782.
11. Filippini G, Farinotti M, Ferrarini M. 2000. Active and passive smoking during pregnancy and risk of central nervous system tumours in children. Paediatr Perinat Epidemiol 14(1):78-84.
12. Filippini G, Farinotti M, Lovicu G, Maisonneuve P, Boyle P. 1994. Mothers' active and passive smoking during pregnancy and risk of brain tumours in children. Int J Cancer 57(6):769-774.
13. Filippini G, Maisonneuve P, McCredie M, Peris-Bonet R, Modan B, Preston-Martin S et al. 2002. Relation of childhood brain tumors to exposure of parents and children to tobacco smoke: The SEARCH international case-control study. surveillance of environmental aspects related to cancer in humans. Int J Cancer 100(2):206-213.
14. Gold EB, Leviton A, Lopez R, Gilles FH, Hedley-Whyte ET, Kolonel LN et al. 1993. Parental smoking and risk of childhood brain tumors. Am J Epidemiol 137(6):620-628.
15. Howe GR, Burch JD, Chiarelli AM, Risch HA, Choi BC. 1989. An exploratory case-control study of brain tumors in children. Cancer Res 49(15):4349-4352.
16. Hu J, Mao Y, Ugnat AM. 2000. Parental cigarette smoking, hard liquor consumption and the risk of childhood brain tumors--a case-control study in northeast china. Acta Oncol 39(8):979-984.
17. Kramer S, Ward E, Meadows AT, Malone KE. 1987. Medical and drug risk factors associated with neuroblastoma: A case-control study. J Natl Cancer Inst 78(5):797-804.
18. Linet MS, Gridley G, Cnattingius S, Nicholson HS, Martinsson U, Glimelius B et al. 1996. Maternal and perinatal risk factors for childhood brain tumors (sweden). Cancer Causes Control 7(4):437-448.
19. McCredie M, Maisonneuve P, Boyle P. 1994. Antenatal risk factors for malignant brain tumours in new south wales children. Int J Cancer 56(1):6-10.
20. Michaelis J, Kaletsch U, Kaatsch P. 2000. Epidemiology of childhood brain tumors. Zentralbl Neurochir 61(2):80-87.
21. Milne E, Greenop KR, Scott RJ, Ashton LJ, Cohn RJ, de Klerk NH et al. 2013. Parental smoking and risk of childhood brain tumors. Int J Cancer 133(1):253-259; doi: 10.1002/ijc.28004 [doi].
22. Norman MA, Holly EA, Ahn DK, PrestonMartin S, Mueller BA, Bracci PM. 1996. Prenatal exposure to tobacco smoke and childhood brain tumors: Results from the united states west coast childhood brain tumor study. Cancer Epidemiology Biomarkers & Prevention 5(2):127-133.
23. Plichart M, Menegaux F, Lacour B, Hartmann O, Frappaz D, Doz F et al. 2008. Parental smoking, maternal alcohol, coffee and tea consumption during pregnancy and childhood malignant central nervous system tumours: The ESCALE study (SFCE). Eur J Cancer Prev 17(4):376-383.
24. Preston-Martin S, Yu MC, Benton B, Henderson BE. 1982. N-nitroso compounds and childhood brain tumors: A case-control study. Cancer Res 42(12):5240-5245.
25. Schuz J, Kaletsch U, Kaatsch P, Meinert R, Michaelis J. 2001a. Risk factors for pediatric tumors of the central nervous system: Results from a german population-based case-control study. Med Pediatr Oncol 36(2):274-282.
26. Schuz J, Kaletsch U, Meinert R, Kaatsch P, Spix C, Michaelis J. 2001c. Risk factors for neuroblastoma at different stages of disease. results from a population-based case-control study in germany. J Clin Epidemiol 54(7):702-709.
27. Yang Q, Olshan AF, Bondy ML, Shah NR, Pollock BH, Seeger RC et al. 2000. Parental smoking and alcohol consumption and risk of neuroblastoma. Cancer Epidemiol Biomarkers Prev 9(9):967-972.
28. Holly EA, Aston DA, Ahn DK, Kristiansen JJ. 1992. Ewing's bone sarcoma, paternal occupational exposure, and other factors. Am J Epidemiol 135(2):122-129.
29. Grufferman S, Schwartz AG, Ruymann FB, Maurer HM. 1993. Parents' use of cocaine and marijuana and increased risk of rhabdomyosarcoma in their children. Cancer Causes Control 4(3):217-224.
30. Grufferman S, Wang HH, DeLong ER, Kimm SY, Delzell ES, Falletta JM. 1982. Environmental factors in the etiology of rhabdomyosarcoma in childhood. J Natl Cancer Inst 68(1):107-113.
31. Magnani C, Pastore G, Luzzatto L, Carli M, Lubrano P, Terracini B. 1989. Risk factors for soft tissue sarcomas in childhood: A case-control study. Tumori 75(4):396-400.
32. Olshan AF, Breslow NE, Falletta JM, Grufferman S, Pendergrass T, Robison LL et al. 1993. Risk factors for Wilms’ tumor. report from the national Wilms’ tumor study. Cancer 72(3):938-944.
33. Schuz J, Kaletsch U, Meinert R, Kaatsch P, Michaelis J. 2001b. High birth weight and other risk factors for Wilms’ tumour: Results of a population-based case-central study. Eur J Pediatr 160(6):333-338.
34. Buckley JD, Sather H, Ruccione K, Rogers PC, Haas JE, Henderson BE et al. 1989. A case-control study of risk factors for hepatoblastoma. A report from the childrens cancer study group. Cancer 64(5):1169-1176.
35. de Fine Licht S, Schmidt LS, Rod NH, Schmiegelow K, Lahteenmaki PM, Kogner P et al. 2012. Hepatoblastoma in the nordic countries. Int J Cancer 131(4):E555-61.
36. Johnson KJ, Williams KS, Ross JA, Krailo MD, Tomlinson GE, Malogolowkin MH et al. 2013. Parental tobacco and alcohol use and risk of hepatoblastoma in offspring: A report from the children's oncology group. Cancer Epidemiol Biomarkers Prev 22(10):1837-1843.
37. Chen Z, Robison L, Giller R, Krailo M, Davis M, Gardner K et al. 2005. Risk of childhood germ cell tumors in association with parental smoking and drinking. Cancer 103(5):1064-1071.
38. Pettersson A, Akre O, Richiardi L, Ekbom A, Kaijser M. 2007. Maternal smoking and the epidemic of testicular cancer--a nested case-control study. Int J Cancer 120(9):2044-2046.
39. Shu XO, Nesbit ME, Buckley JD, Krailo MD, Robinson LL. 1995. An exploratory analysis of risk factors for childhood malignant germ-cell tumors: Report from the childrens cancer group (canada, united states). Cancer Causes Control 6(3):187-198.
40. Tuomisto J, Holl K, Rantakokko P, Koskela P, Hallmans G, Wadell G et al. 2009. Maternal smoking during pregnancy and testicular cancer in the sons: A nested case-control study and a meta-analysis. Eur J Cancer 45(9):1640-1648;.
41. Abadi-Korek I, Stark B, FAU - Zaizov R, Zaizov R, FAU - Shaham J, Shaham J. Parental occupational exposure and the risk of acute lymphoblastic leukemia in offspring in israel. - J Occup Environ Med.2006 Feb;48(2):165-74.
42. Alexander FE, Patheal SL, Biondi A, Brandalise S, Cabrera ME, Chan LC et al. 2001. Transplacental chemical exposure and risk of infant leukemia with MLL gene fusion. Cancer Res 61(6):2542-2546.
43. Brondum J, Shu XO, Steinbuch M, Severson RK, Potter JD, Robison LL. 1999. Parental cigarette smoking and the risk of acute leukemia in children. Cancer 85(6):1380-1388.
44. Castro-Jimenez MA, Orozco-Vargas LC. 2011. Parental exposure to carcinogens and risk for childhood acute lymphoblastic leukemia, colombia, 2000-2005. Prev Chronic Dis 8(5):A106.
45. Farioli A, Legittimo P, Mattioli S, Miligi L, Benvenuti A, Ranucci A et al. 2014. Tobacco smoke and risk of childhood acute lymphoblastic leukemia: Findings from the SETIL case-control study. Cancer Causes Control 25(6):683-692.
46. Ferreira JD, Couto AC, Pombo-de-Oliveira MS, Koifman S, Brazilian Collaborative Study Group of Infant Acute Leukemia. 2012. Pregnancy, maternal tobacco smoking, and early age leukemia in brazil. Front Oncol 2:151.
47. Infante-Rivard C, Krajinovic M, Labuda D, Sinnett D. 2000. Parental smoking, CYP1A1 genetic polymorphisms and childhood leukemia (quebec, canada). Cancer Causes Control 11(6):547-553.
48. Lariou MS, Dikalioti SK, Dessypris N, Baka M, Polychronopoulou S, Athanasiadou-Piperopoulou F et al. 2013. Allergy and risk of acute lymphoblastic leukemia among children: A nationwide case control study in greece. Cancer Epidemiol 37(2):146-151.
49. Mattioli S, Farioli A, Legittimo P, Miligi L, Benvenuti A, Ranucci A et al. 2014. Tobacco smoke and risk of childhood acute non-lymphocytic leukemia: Findings from the SETIL study. PLoS One 9(11):e111028.
50. Menegaux F, Ripert M, Hemon D, Clavel J. 2007. Maternal alcohol and coffee drinking, parental smoking and childhood leukaemia: A french population-based case-control study. Paediatr Perinat Epidemiol 21(4):293-299.
51. Milne E, Greenop KR, Scott RJ, Bailey HD, Attia J, Dalla-Pozza L et al. 2012. Parental prenatal smoking and risk of childhood acute lymphoblastic leukemia. Am J Epidemiol 175(1):43-53.
52. Oksuzyan S, Crespi CM, Cockburn M, Mezei G, Kheifets L. 2012. Birth weight and other perinatal characteristics and childhood leukemia in california. Cancer Epidemiol 36(6):e359-65.
53. Orsi L, Rudant J, Ajrouche R, Leverger G, Baruchel A, Nelken B et al. 2015. Parental smoking, maternal alcohol, coffee and tea consumption during pregnancy, and childhood acute leukemia: The ESTELLE study. Cancer Causes Control; doi: 10.1007/s10552-015-0593-5.
54. Petridou E, Trichopoulos D, Kalapothaki V, Pourtsidis A, Kogevinas M, Kalmanti M et al. 1997. The risk profile of childhood leukaemia in greece: A nationwide case-control study. Br J Cancer 76(9):1241-1247.
55. Podvin D, Kuehn CM, Mueller BA, Williams M. 2006. Maternal and birth characteristics in relation to childhood leukaemia. Paediatr Perinat Epidemiol 20(4):312-322.
56. Rosenbaum PF, Buck GM, Brecher ML. 2005. Allergy and infectious disease histories and the risk of childhood acute lymphoblastic leukaemia. Paediatr Perinat Epidemiol 19(2):152-164.
57. Schraw JM, Dong YQ, Okcu MF, Scheurer ME, Forman MR. 2014. Do longer formula feeding and later introduction of solids increase risk for pediatric acute lymphoblastic leukemia? Cancer Causes Control 25(1):73-80.
58. Severson RK, Buckley JD, Woods WG, Benjamin D, Robison LL. 1993. Cigarette smoking and alcohol consumption by parents of children with acute myeloid leukemia: An analysis within morphological subgroups--a report from the childrens cancer group. Cancer Epidemiol Biomarkers Prev 2(5):433-439.
59. Shu XO, Ross JA, Pendergrass TW, Reaman GH, Lampkin B, Robison LL. 1996. Parental alcohol consumption, cigarette smoking, and risk of infant leukemia: A childrens cancer group study. J Natl Cancer Inst 88(1):24-31.
60. Shu XO, Linet MS, Steinbuch M, Wen WQ, Buckley JD, Neglia JP et al. 1999. Breast-feeding and risk of childhood acute leukemia. J Natl Cancer Inst 91(20):1765-1772.
61. Slater ME, Linabery AM, Blair CK, Spector LG, Heerema NA, Robison LL et al. 2011. Maternal prenatal cigarette, alcohol and illicit drug use and risk of infant leukaemia: A report from the children's oncology group. Paediatr Perinat Epidemiol 25(6):559-565.
62. van Duijn CM, van Steensel-Moll HA, Coebergh JW, van Zanen GE. 1994. Risk factors for childhood acute non-lymphocytic leukemia: An association with maternal alcohol consumption during pregnancy? Cancer Epidemiol Biomarkers Prev 3(6):457-460.
63. Greenop KR, Blair EM, Bower C, Armstrong BK, Milne E. Factors relating to pregnancy and birth and the risk of childhood brain tumors: results from an Australian case-control study. Pediatr Blood Cancer 2014 Mar;61(3):493-498.
64. Milne E, Royle JA, de Klerk NH, Blair E, Bailey H, Cole C, et al. Fetal growth and risk of childhood acute lymphoblastic leukemia: results from an Australian case-control study. Am J Epidemiol 2009 Jul 15;170(2):221-228.
65. Milne E, Royle JA, Bennett LC, de Klerk NH, Bailey HD, Bower C, et al. Maternal consumption of coffee and tea during pregnancy and risk of childhood ALL: results from an Australian case-control study. Cancer Causes & Control 2011 FEB 2011;22(2):207-218.
66. Reid A, Glass DC, Bailey HD, Milne E, de Klerk NH, Downie P, et al. Risk of childhood acute lymphoblastic leukaemia following parental occupational exposure to extremely low frequency electromagnetic fields. Br J Cancer 2011 Oct 25;105(9):1409-1413.
67. Bonaventure A, Goujon-Bellec S, Rudant J, Orsi L, Leverger G, Baruchel A, et al. Maternal smoking during pregnancy, genetic polymorphisms of metabolic enzymes, and childhood acute leukemia: the ESCALE study (SFCE). Cancer Causes Control 2012 Feb;23(2):329-345.
68. Bonaventure A, Rudant J, Goujon-Bellec S, Orsi L, Leverger G, Baruchel A, et al. Childhood acute leukemia, maternal beverage intake during pregnancy, and metabolic polymorphisms. Cancer Causes Control 2013 Apr;24(4):783-793.
69. Clavel J, Bellec S, Rebouissou S, Menegaux F, Feunteun J, Bonaiti-Pellie C, et al. Childhood leukaemia, polymorphisms of metabolism enzyme genes, and interactions with maternal tobacco, coffee and alcohol consumption during pregnancy. Eur J Cancer Prev 2005 Dec;14(6):531-540.
70. Menegaux F, Steffen C, Bellec S, Baruchel A, Lescoeur B, Leverger G, et al. Maternal coffee and alcohol consumption during pregnancy, parental smoking and risk of childhood acute leukaemia. Cancer Detect Prev 2005;29(6):487-493.
71. Metayer C, Zhang L, Wiemels JL, Bartley K, Schiffman J, Ma X, et al. Tobacco smoke exposure and the risk of childhood acute lymphoblastic and myeloid leukemias by cytogenetic subtype. Cancer Epidemiol Biomarkers Prev 2013 Sep;22(9):1600-1611.
72. Chang JS, Selvin S, Metayer C, Crouse V, Golembesky A, Buffler PA. Parental smoking and the risk of childhood leukemia. Am J Epidemiol 2006 Jun 15;163(12):1091-1100.
73. Kwan ML, Jensen CD, Block G, Hudes ML, Chu LW, Buffler PA. Maternal diet and risk of childhood acute lymphoblastic leukemia. Public Health Rep 2009 Jul-Aug;124(4):503-514.
74. Barrington-Trimis JL, Searles Nielsen S, Preston-Martin S, Gauderman WJ, Holly EA, Farin FM, et al. Parental smoking and risk of childhood brain tumors by functional polymorphisms in polycyclic aromatic hydrocarbon metabolism genes. PLoS One 2013 Nov 18;8(11):e79110.
75. Winn DM, Li FP, Robison LL, Mulvihill JJ, Daigle AE, Fraumeni JF. A Case-Control Study of the Etiology of Ewing Sarcoma. Cancer Epidemiology Biomarkers & Prevention 1992 NOV-DEC 1992;1(7):525-532.
76. McLaughlin CC, Baptiste MS, Schymura MJ, Nasca PC, Zdeb MS. Maternal and infant birth characteristics and hepatoblastoma. Am J Epidemiol 2006 May 1;163(9):818-828.
77. MacArthur AC, McBride ML, Spinelli JJ, Tamaro S, Gallagher RP, Theriault G. Risk of childhood leukemia associated with parental smoking and alcohol consumption prior to conception and during pregnancy: the cross-Canada childhood leukemia study. Cancer Causes Control 2008 Apr;19(3):283-295.
78. Infante-Rivard C, Krajinovic M, Labuda D, Sinnett D. Childhood acute lymphoblastic leukemia associated with parental alcohol consumption and polymorphisms of carcinogen-metabolizing genes. Epidemiology 2002 May;13(3):277-281.
79. Cnattingius S, Zack M, Ekbom A, Gunnarskog J, Linet M, Adami HO. Prenatal and neonatal risk factors for childhood myeloid leukemia. Cancer Epidemiol Biomarkers Prev 1995 Jul-Aug;4(5):441-445.
80. Adami J, Glimelius B, Cnattingius S, Ekbom A, Zahm SH, Linet M, et al. Maternal and perinatal factors associated with non-Hodgkin's lymphoma among children. International Journal of Cancer 1996 MAR 15 1996;65(6):774-777.
81. Schuz J, Forman MR. Birthweight by gestational age and childhood cancer. Cancer Causes Control 2007 Aug;18(6):655-663.
82. Kaatsch P, Kaltesch U, Meinert R, Michaelis J. An extended study on childhood malignancies in the vicinity of German nuclear power plants. Cancer Causes Control 1998 Oct; 9(5): 529-533.
83. Petridou E, Ntouvelis E, Dessypris N, Terzidis A, Trichopoulos D, Childhood Hematology-Oncology Group. Maternal diet and acute lymphoblastic leukemia in young children. Cancer Epidemiol Biomarkers Prev 2005 Aug;14(8):1935-1939.
84. Diamantaras AA, Dessypris N, Sergentanis TN, Ntouvelis E, Athanasiadou-Piperopoulou F, Baka M, et al. Nutrition in early life and risk of childhood leukemia: a case-control study in Greece. Cancer Causes Control 2013 Jan;24(1):117-124.
85. van Steensel-Moll HA, Valkenburg HA, Vandenbroucke JP, van Zanen GE. Are maternal fertility problems related to childhood leukaemia? Int J Epidemiol 1985 Dec;14(4):555-559.
86. Stavrou EP, Baker DF, Bishop JF. Maternal smoking during pregnancy and childhood cancer in New South Wales: a record linkage investigation. Cancer Causes Control 2009 Nov;20(9):1551-1558.
87. Pershagen G, Ericson A, Otterblad-Olausson P. Maternal smoking in pregnancy: does it increase the risk of childhood cancer? Int J Epidemiol 1992 Feb;21(1):1-5.
88. Cochrane Collaboration. 2011. 9.5.2 Identifying and measuring heterogeneity. In: Cochrane handbook for systematic reviews of interventions. (Higgins JPT and Green S, eds). Available: <http://handbook.cochrane.org/chapter_9/9_5_2_identifying_and_measuring_heterogeneity.htm> [accessed 3 December 2015].
89. Higgins J, Thompson S, Deeks J, Altman D, 2003. Measuring inconsistency in meta-analyses. BMJ. 2003 Sep 6; 327(7414): 557–560.
90. Egger M, Davey Smith G, Schneider M, Minder C. Bias in meta-analysis detected by a simple, graphical test. BMJ 1997 Sep 13;315(7109):629-634.
